# Iron-catalysed cross-coupling of organolithium compounds with organic halides

**DOI:** 10.1038/ncomms10614

**Published:** 2016-02-05

**Authors:** Zhenhua Jia, Qiang Liu, Xiao-Shui Peng, Henry N. C. Wong

**Affiliations:** 1 Department of Chemistry, State Key Laboratory of Synthetic Chemistry and Centre of Novel Functional Molecules, Chinese University of Hong Kong, Shatin, New Territories, Hong Kong SAR, China; 2 Shenzhen Center of Novel Functional Molecules and Shenzhen Municipal Key Laboratory of Chemical Synthesis of Medicinal Organic Molecules, Shenzhen Research Institute, Chinese University of Hong Kong, No.10, Second Yuexing Road, Shenzhen 518507, China

## Abstract

In past decades, catalytic cross-coupling reactions between organic halides and organometallic reagents to construct carbon–carbon bond have achieved a tremendous progress. However, organolithium reagents have rarely been used in cross-coupling reactions, due mainly to their high reactivity. Another limitation of this transformation using organolithium reagents is how to control reactivity with excellent selectivity. Although palladium catalysis has been applied in this field recently, the development of an approach to replace catalytic systems of noble metals with nonprecious metals is currently in high demand. Herein, we report an efficient synthetic protocol involving iron-catalysed cross-coupling reactions employing organolithium compounds as key coupling partners to unite aryl, alkyl and benzyl fragments and also disclose an efficient iron-catalysed release-capture ethylene coupling with isopropyllithium.

## Abstract (web summary)

Cross coupling reactions of organolithiums are challenging due to their high reactivity, though palladium catalysed versions have been reported. Here, the authors report the cross coupling of organolithiums with aryl halides with inexpensive and widely available iron catalysts.

## Graphical abstract

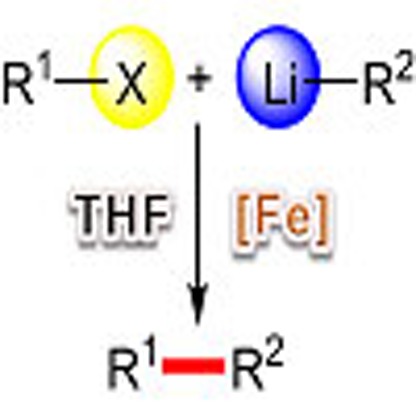

Transition metal-catalysed cross-coupling has emerged as a highly useful, selective and widely applicable method for synthesizing structurally diverse organic compounds via carbon–carbon bond formation^1^,^2^. Since the discoveries of cross-coupling reactions, palladium-catalysed cross-coupling with organic halides and organometallic reagents, has dominated this area as an exceptionally powerful approach to assemble C–C bond (Fig. 1a)^3^. Although Murahashi *et al*. disclosed a palladium-catalysed cross-coupling reaction of alkenyl halides with various organolithium compounds, direct use of organolithium reagents in cross-coupling reactions has been neglected for a long time, mainly due to the high reactivity and low stability of organolithium reagents^4^,^5^,^6^,^7^. Recently, Feringa and co-workers developed palladium-based catalytic systems to directly generate C–C bond using organolithium compounds as cross-coupling partners (Fig. 1b)^8^,^9^,^10^,^11^,^12^,^13^. Although palladium-based catalysts typically mediated such reactions, there are increasing concerns about their long-term sustainability in the synthetic community because of its high cost, low natural abundance, environmentally deleterious extraction, toxicity and competition for its use from the automotive and consumer electronics sectors^14^. Therefore, there is a growing interest in replacing palladium-based catalysts with those more Earth-abundant elements. With its low cost, high natural abundance and low toxicity, iron is indeed a particularly appealing alternative, and accordingly, the development of iron-catalysed cross-coupling is undergoing an explosive growth^15^,^16^,^17^,^18^,^19^,^20^. Herein, we develop an iron-catalysed cross-coupling strategy of organolithium reagents with organic halides to form C–C bonds, examples including C(*sp*^2^)-C(*sp*^3^) bonds, C(*sp*^3^)-C(*sp*^3^) bonds and a rare method to form a novel C(*sp*^2^)-C(*sp*^3^) bond via *in-situ* generation of ethylene from tetrahydrofuran (THF).

## Results

### Serendipity

**Figure 2:**
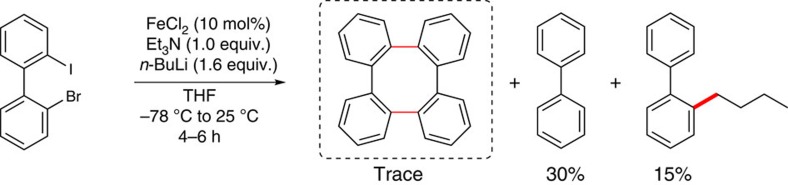
Serendipity induced the discovery of iron-catalysed cross-coupling of organolithium compounds. Dimerization of 2-bromo-2′-iodo-1,1′-biphenyl to synthesize tetraphenylene via a one-pot iron-catalysed intramolecular cross-coupling protocol.

Previously, we demonstrated that the rigid tetraphenylene (tetrabenzo[*a,c,e,g*]cyclooctatetraene) is a structurally and functionally exceptional molecule^21^,^22^. To improve the efficiency of the coupling step, we proposed to synthesize tetraphenylene derivatives through a one-pot iron-catalysed intramolecular cross-coupling protocol^23^. Although we obtained a trace amount of tetraphenylene, the serendipity is that 2-*n*-butylbiphenyl was observed (Fig. 2). Therefore, we recognized the potential use of alkyllithum reagents in iron-catalysed cross-coupling reactions.

### Optimization

**Figure 3:**
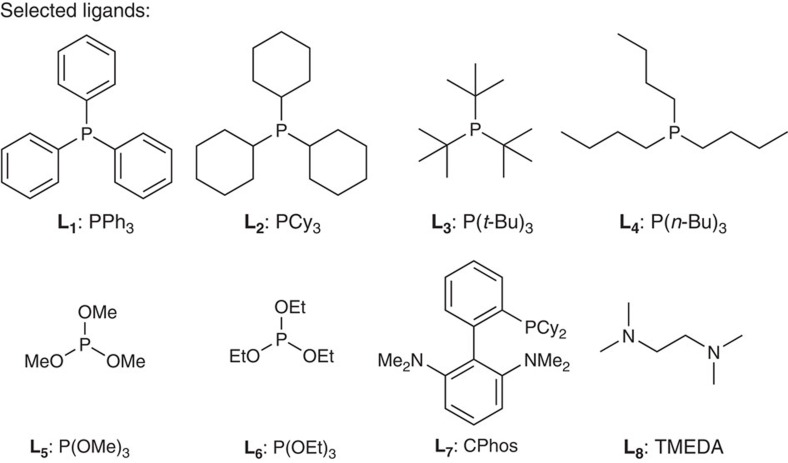
Selected ligands. Monodentate and bidentate ligands were screened, also see Supplementary Information.

Encouraged by the reaction shown in Fig. 2, we attempted to couple 4-methoxybromobenzene (**1a**) with *n*-BuLi (**2a**) under the same condition. As expected, the target product *p*-methoxybutylbenzene (**3a**), together with a trace amount of the isomerized cross-coupling product (**3a'**), were detected by gas chromatography-mass spectrometry (GC–MS), although the homo-coupling product (**4**) is the major product, together with dehalogenated product (**5**; Table 1, entry 1). In the absence of triethylamine (Et_3_N) and at 22 °C, the ratio of the desired product was almost the same (Table 1, entries 2–3). Then, in the presence of FeCl_2_ (10 mol%), ligands (20 mol%) and **1a** (0.2 mmol) in THF (1.0 ml), several traditional bidentate ligands with different bite angles (for expanded screening results, see Supplementary Table 1) and monodentate electron-rich phosphine ligands (Fig. 3) were examined through a slow addition of dilute organolithium reagent **2a** (0.3 mmol, 0.35 M) within 1 h at 22 °C using a syringe pump (Table 1, entries 4–11). To our delight, a distinct improvement was displayed by GC–MS. When trimethyl phosphite (L_5_) was used as a ligand, the desired product was isolated in 68% yield (Table 1, entry 8). Further screening of iron salts revealed that iron(II) chloride gave a promising yield with trimethyl phosphite as the ligand (Table 1, entries 12–15). Recently, Fürstner pioneered the use of iron(III) acetylactonate-catalysed cross-coupling reactions with Grignard reagents^24^,^25^,^26^,^27^, however, this iron catalyst demonstrated lower reactivity with lithium reagents (Table 1, entry 16). Surprisingly, upon addition of tetramethylethylenediamine (TMEDA, L_8_) into the solution of iron(III) chloride in THF, the homo-coupling by-product (**4**) was suppressed dramatically (Table 1, entry 17). This catalytic system developed by Nakamura had been employed in cross-coupling reactions with Grignard reagents and arylzinc reagents^28^,^29^,^30^,^31^. Therefore, the complex of TMEDA with iron(III) chloride was prepared according to Nakamura's procedure for use in our next stage of optimization (Table 1, entries 18–24). When the cross-coupling reaction was conducted at 0 °C, the generation of the dehalogenated by-product (**5**) was reduced and the expected product was isolated in 85% (0.2 mmol scale; Table 1, entry 19). To further improve this procedure, we also screened the reaction media. A comparison of results obtained in THF revealed that the ratios of desired product were decreased in toluene and diethyl ether (Table 1, entries 20–21). When the catalyst loading was reduced to 3 and 1 mol%, respectively, the overall efficiency was not reduced in an obvious manner (Table 1, entries 22–23).

### C(*sp* ^2^)-C(*sp* ^3^) cross-coupling of aryl halides with alkyllithiums

To expand the scope of the iron-catalysed reactions, C(*sp*^2^)-C(*sp*^3^) cross-coupling of aryl halides with alkyllithium reagents was further investigated. Initially, we compared the reactivity of different aryl halides (Table 2, **3a**). 4-Methoxychlorobenzene (5% conversion) was more inert than 4-methoxybromobenzene (**1a**). The target product was generated exclusively, when 4-methoxyiodobenzene was used as a starting substrate. Unexpectedly, 4-methoxyphenyltrifluoromethanesulfonyl triflate (an aryl triflate) decomposed to the corresponding phenol (see note in Table 2). In consideration of their commercial availability, we made use of aryl bromides for further investigation (Table 2, 3**b**-**3**r). It was uncovered that varying the position of the methoxy group on the benzene ring led to a pronounced effect on the reaction outcome, presumably due to chelation of oxygen with lithium (Table 2, 3**b**-**3c**). In the case of bromobenzene, the GC yield was given due to the volatility issue (Table 2, **3d**). Electron-donating and bulky functional groups facilitated cross-coupling reaction without sacrificing the yield of the corresponding products (Table 2, **3e**-**3g**). However, a strongly electron-withdrawing substituent was found to lead to halogen-metal exchange (Table 2, **3h**). Remarkably, a series of alkyllithiums were freshly prepared and were found to be compatible with this protocol, being able to couple with 4-bromo-*N,N*-dimethylaniline (Table 2, **3i**-**3o**). Polyaromatic compounds were found to undergo alkylation in moderate yields (Table 2, **3p**-**3q**). In addition, a double alkylation product was obtained in 65% yield (Table 2, **3r**).

### Release-capture ethylene coupling with isopropyllithium

**Figure 4:**
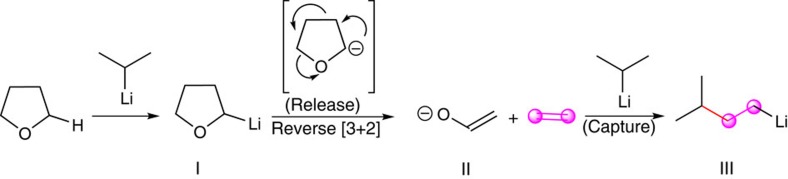
Possible pathway of release-capture ethylene. (**I**) 2-LithioTHF. (**II**) Lithium enolate. (**III**) Doubly homologated of isopropyllithium to generate the isopentyllithium *in situ*.

When isopropyllithium, a typical secondary organolithium, was utilized in the iron catalysis system with 4-methoxybromobenzene (**1a**), 1-isopentyl-4-methoxybenzene (**3a**_**THF**_) was obtained together with a trace amount of cross-coupling product 1-isopropyl-4-methoxybenzene. After prolonging the reaction time to overnight at 22 °C, the yield of **3a**_**THF**_ was optimized up to 71% (Table 3, **3a**_**THF**_). To our best knowledge, this is an unusual example of transition metal-catalysed cross-coupling reaction involving freshly prepared ethylene generated by decomposing THF with isopropyllithium. Several aryl bromides were then investigated to explore the substituent effect at various positions of the benzene ring. Possible chelation effect and steric effect were demonstrated when the benzene *ortho*-position was occupied by a methoxy group or a bulky group (Table 3, **3b**_**THF**_ **and 3c**_**THF**_). It is noted that when FeCl_2_ with P(OMe)_3_ was used as the catalyst in place of [(FeCl_3_)_2_(TMEDA)_3_], the yield of **3c**_**THF**_ could be improved. Remote substituents could be tolerated, leading to the formation of the corresponding products in 37–77% yield (Table 3, **3d**_**THF**_-**3f**_**THF**_). Moreover, a naphthyl compound was found to participate efficiently (Table 3, **3g**_**THF**_). On the basis of the previously reported reactions^32^, we would like to propose a plausible pathway for this release-capture ethylene process. Thus, as shown in Fig. 4, THF is deprotonated at its 2-position by isopropyllithium to form 2-lithioTHF (**I**). Then, a subsequent intramolecular reverse [3+2] cycloaddition of the anion would release ethylene and generate the lithium enolate (**II**). Finally, the resulting ethylene could be caught *in situ* to give the doubly homologated lithium product (**III**). Moreover, further evidence for our proposed pathway was obtained from a relevant deuterium-labelled crossover experiment utilizing deuterated tetrahydrofuran (THF-d_8_) as solvent. Thus, treatment of 4-methoxybromobenzene (**1a**) with isopropyllithium in THF-d_8_, led to the release of ethylene-d_4_. The expected deuterated product **3a**_**THF-d8**_ (Table 3) was obtained in 61% yield.

### Cross-coupling of alkyl bromides with organolithiums

We next extended the iron catalysis strategy to alkyl bromides with organolithium reagents. Typically, commercially available 1-bromo-3-phenylpropane was assessed with *n*-BuLi to explore the possibility of C(*sp*^3^)-C(*sp*^3^) cross-coupling. Gratifyingly, the reaction proceeded smoothly and the desired product was isolated in 77% yield (Table 4, **3aa**). Other organolithium reagents, such as cyclopropyllithium, 9*H*-fluoren-9-yllithium and (trimethylsilyl)methyllithium, were allowed to couple with 1-bromo-3-phenylpropane to provide the corresponding C(*sp*^3^)-C(*sp*^3^) cross-coupling products in good to excellent yields (Table 4, **3ab-3ad**). Benzylic compounds, possessing a typical C(*sp*^3^)-Br bonds, were also used as coupling partners. As expected, the cross-coupling products with yields ranging from 11 to 71% were generated, when *n*-BuLi and (trimethylsilyl)methyllithium were used as coupling partners (Table 4, **3ae-3am**). Under the same condition, 2-(3-bromopropyl)naphthalene was also alkylated (Table 4, **3an**). Subsequently, bromocyclohexane successfully underwent a similar reaction to form the relevant coupling product in 44% yield (Table 4, **3ao**).

In summary, we have disclosed iron-catalysed cross-coupling of organolithium compounds to form diverse carbon–carbon bonds efficiently. These results are expected to expand the scope of iron catalysis as well as the use of organolithium reagents. We trust that these reactions would provide milder, cheaper and more environmentally friendly approaches towards cross-coupling products. An extension of this catalytic system to broaden its scope, and to investigate its mechanistic nature is underway in our laboratory.

## Discussion

To provide a support against the involvement of trace amounts of other metal species, such as Pd, Pt, Co and Ni in our iron catalysts that would catalyse C–C bond formation, inductively coupled plasma mass spectrometry was performed on samples of FeCl_3_ to detect the trace quantities of these metals (see Supplementary Information for details). Moreover, we conducted experiments to mimic the catalyst system to prove that relevant products were not isolated when the concentration of Co and Ni were as low as those present in the iron salts (see Supplementary Information for details). We also performed preliminary mechanistic analysis of this transformation utilizing several control experiments (see Supplementary Information for details). It was likely that the reaction involved radical species.

**Figure 5:**
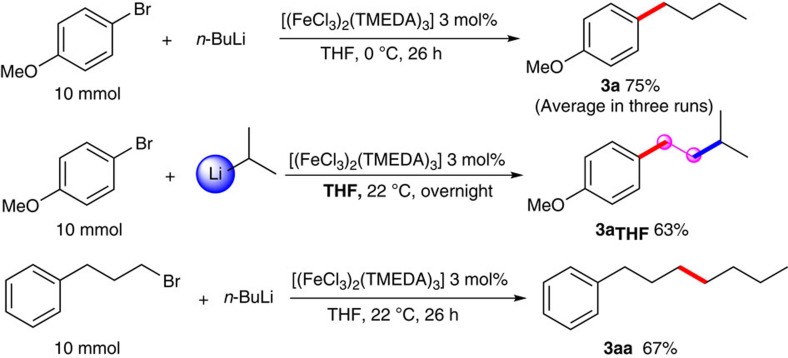
Gram scale reactions. Three model substrates were selected to scale up to 10 mmol scale and the corresponding target products were isolated in satisfied yields.

Noteworthy, the capability to procure useful product quantities for laboratory and industry usage through scalable routes is emerging as a very essential goal in catalytic reactions today. Therefore, we also confirmed the scalable feasibility of these iron-catalysed reactions, as shown in Fig. 5. As can be seen, several typical scale-up reactions in multi-gram scales provided relevant desired products in satisfied yields.

## Methods

### Iron-catalysed cross-coupling of 4-methoxybromobenzene (**1a**) and *n*-BuLi (**2a**)

To an oven-dried vial, equipped with a magnetic stirring bar, was charged with [(FeCl_3_)_2_(TMEDA)_3_] (3.96 mg, 0.006 mmol, 3 mol%) in a glove box, following by the subsequent addition of 4-methoxybromobenzene (**1a**; 0.2 mmol) and THF (1.0 ml). Then, after the sealed vial with a rubber stopper was taken out from the glove box, the reaction mixture was cooled to 0 °C, *n*-BuLi (**2a;** 0.30 mmol, 1.6 M or 2.4 M in hexane, diluted with THF to a final concentration of 0.35 M) was added to the mixture using a syringe pump in 1 h. After the addition was completed, the reaction mixture was stirred at 0 °C for 1 h. Then, after quenching with a saturated solution of aqueous NH_4_Cl, the reaction mixture was extracted with CH_2_Cl_2_ three times. The combined organic solvent was evaporated under reduced pressure to afford the crude product, which was then purified by column chromatography on silica gel or preparative thin-layer chromatography.

## Additional information

**How to cite this article**: Jia, Z. *et al*. Iron-catalysed cross-coupling of organolithium compounds with organic halides. *Nat. Commun.* 7:10614 doi: 10.1038/ncomms10614 (2016).

## Supplementary Material

Supplementary Information Supplementary Figures 1-72, Supplementary Tables 1-2, Supplementary Methods and Supplementary References

## Figures and Tables

**Figure 1:**
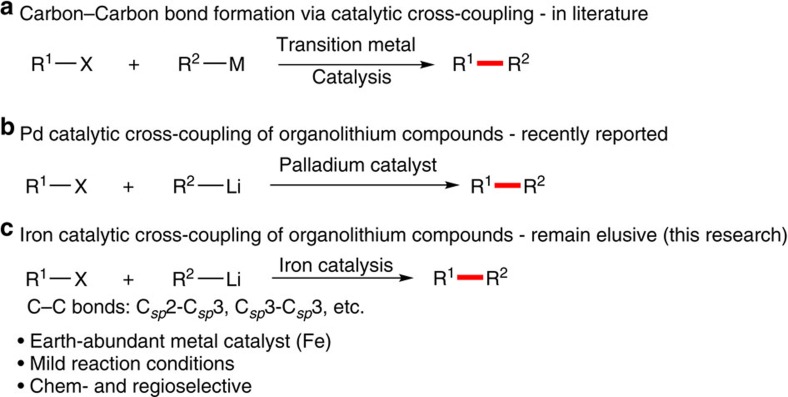
Transition metal-catalysed cross-coupling to form carbon–carbon bonds. (**a**) C–C bond formation via catalytic cross-coupling. (**b**) Palladium-catalysed cross-coupling of organolithium compounds. (**c**) Iron-catalysed cross-coupling of organolithium reagents.

**Table 1:**
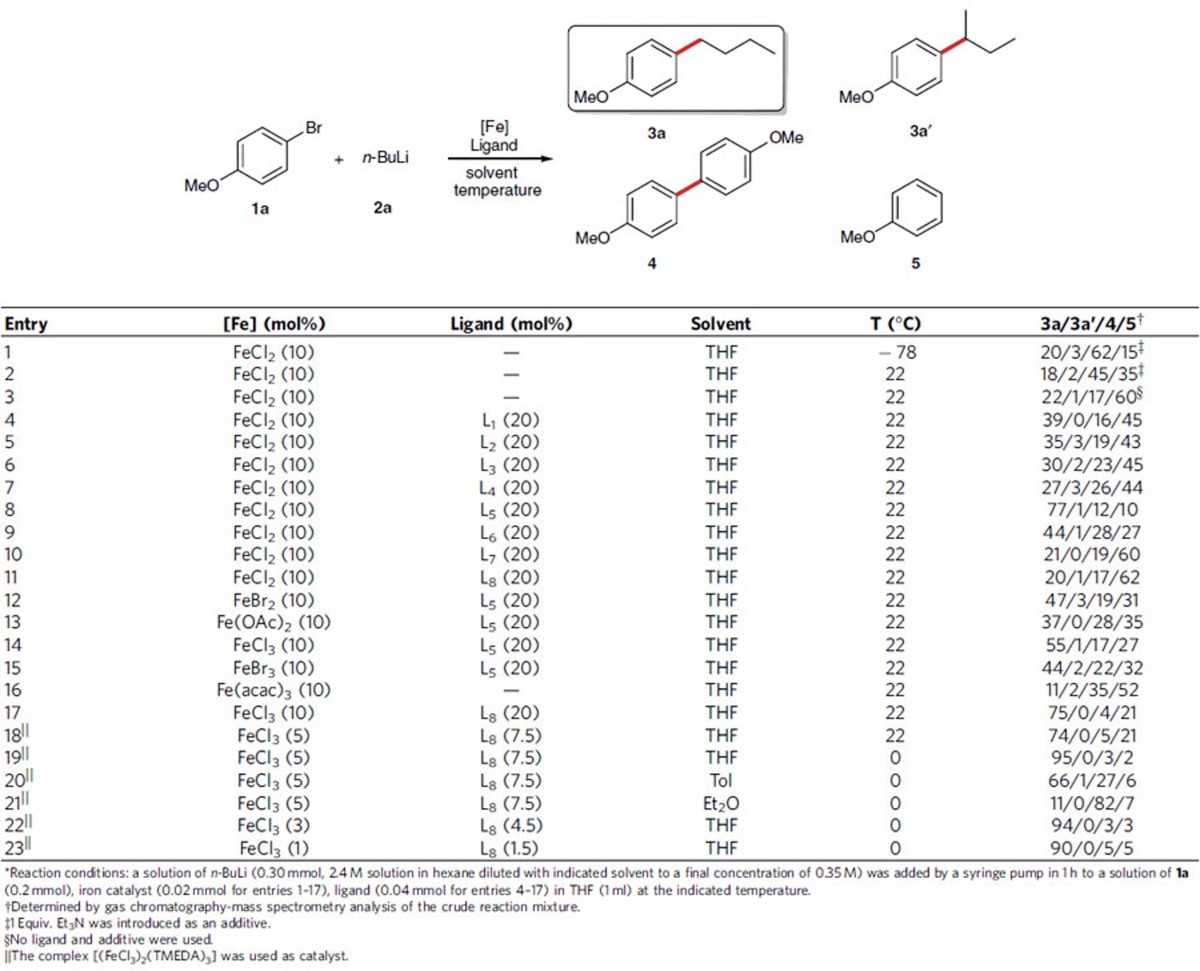
Selected optimization results for C(*sp*^2^)-C(*sp*^3^) cross-coupling of 4-methoxybromobenzene (1a) and *n*-BuLi (2a)*.

**Table 2:**
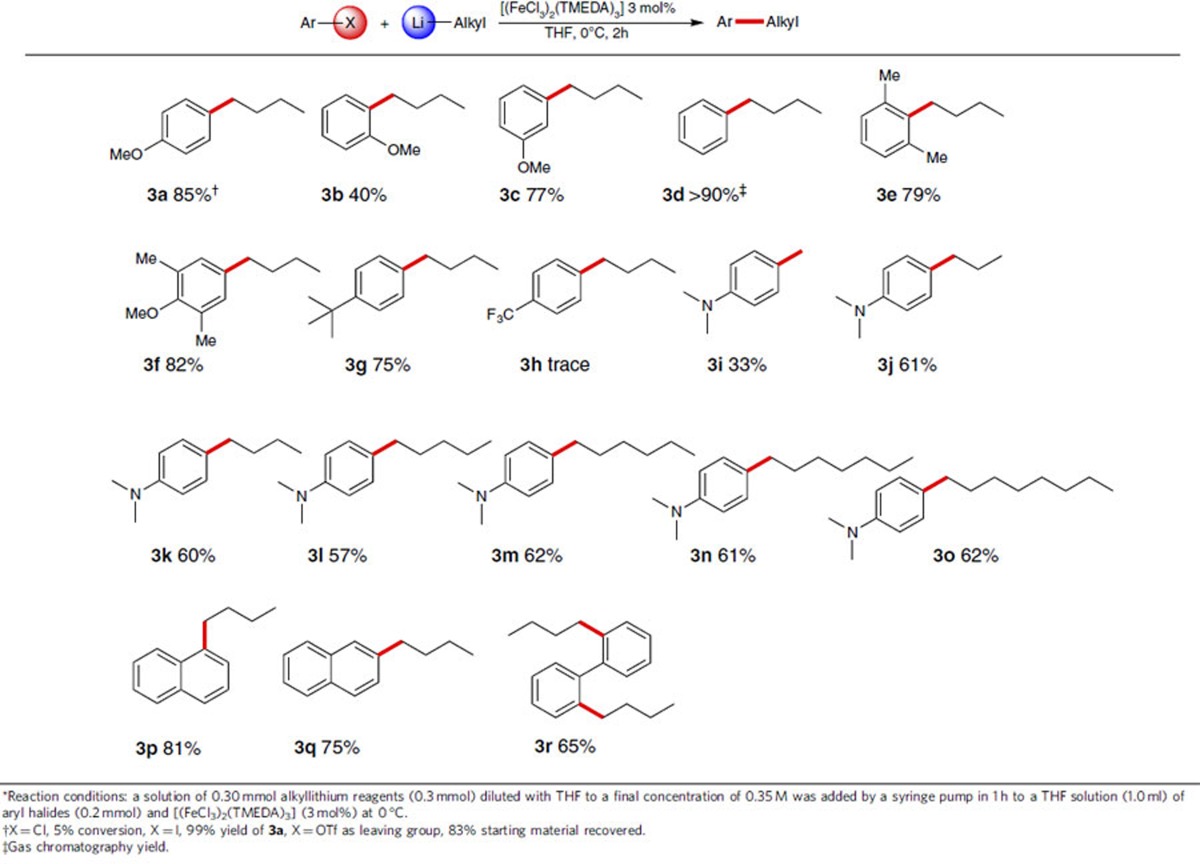
Iron-catalysed cross-coupling of aryl halides with alkyllithium reagents*.

^*^Reaction conditions: a solution of 0.30 mmol alkyllithium reagents (0.3 mmol) diluted with THF to a final concentration of 0.35 M was added by a syringe pump in 1 h to a THF solution (1.0 ml) of aryl halides (0.2 mmol) and [(FeCl_3_)_2_(TMEDA)_3_] (3 mol%) at 0 °C.

^†^X=Cl, 5% conversion, X=I, 99% yield of **3a**, X=OTf as leaving group, 83% starting material recovered.

^‡^Gas chromatography yield.

**Table 3:**
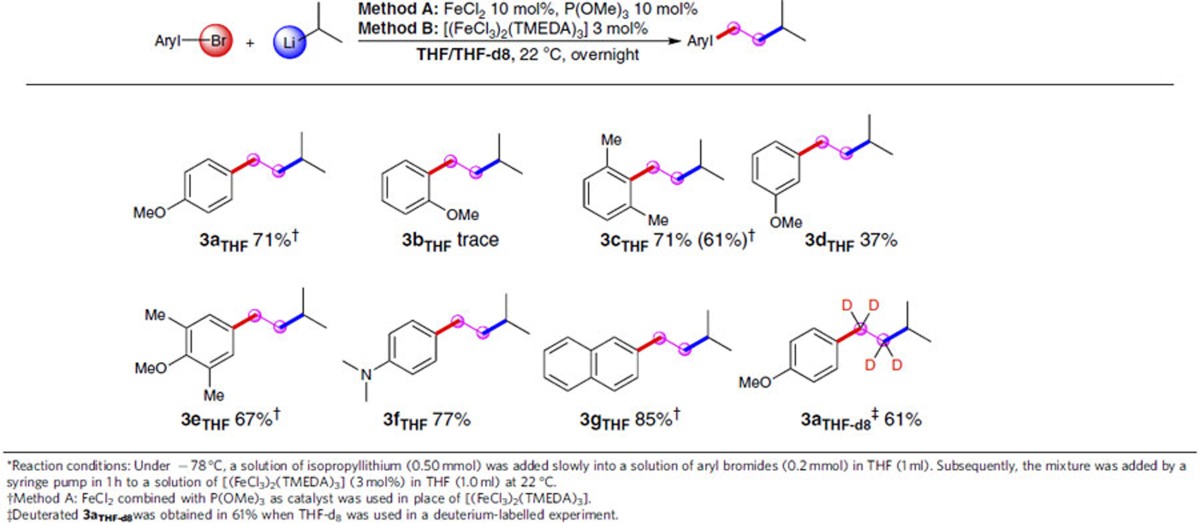
Iron-catalysed release-capture ethylene coupling with isopropyllithium*.

^*^Reaction conditions: Under −78 °C, a solution of isopropyllithium (0.50 mmol) was added slowly into a solution of aryl bromides (0.2 mmol) in THF (1 ml). Subsequently, the mixture was added by a syringe pump in 1 h to a solution of [(FeCl_3_)_2_(TMEDA)_3_] (3 mol%) in THF (1.0 ml) at 22 °C.

^†^Method A: FeCl_2_ combined with P(OMe)_3_ as catalyst was used in place of [(FeCl_3_)_2_(TMEDA)_3_].

^‡^Deuterated **3a**_**THF-d8**_was obtained in 61% when THF-d_8_ was used in a deuterium-labelled experiment.

**Table 4:**
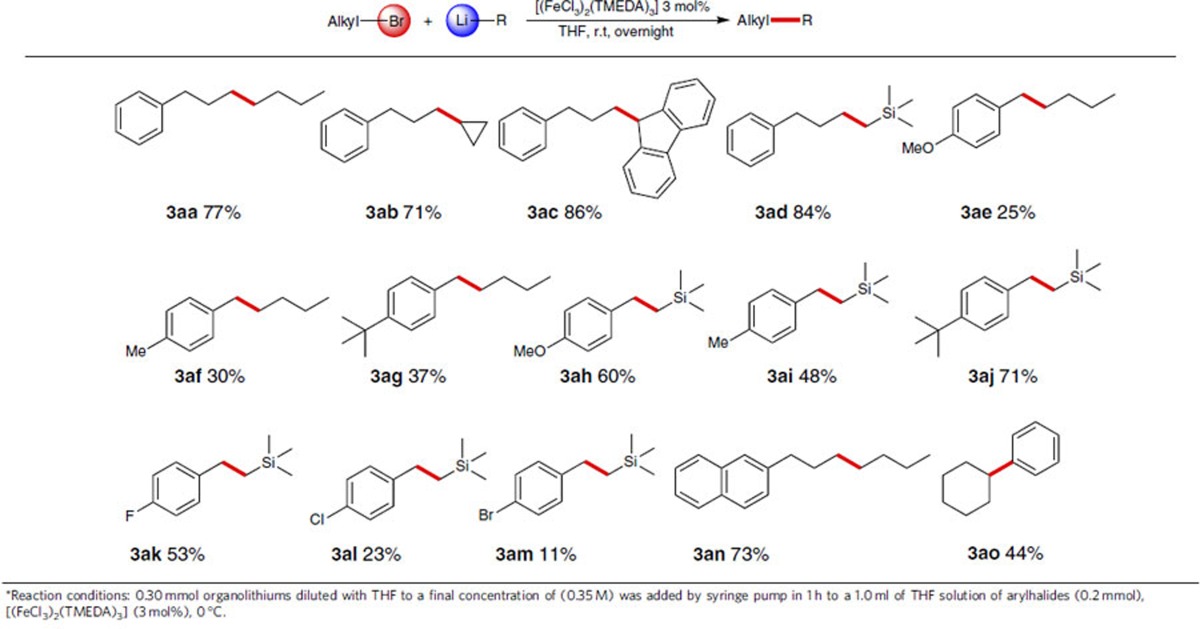
Iron-catalysed cross-coupling of alkyl bromides with organolithium reagents*.

^*^Reaction conditions: 0.30 mmol organolithiums diluted with THF to a final concentration of (0.35 M) was added by syringe pump in 1 h to a 1.0 ml of THF solution of arylhalides (0.2 mmol), [(FeCl_3_)_2_(TMEDA)_3_] (3 mol%), 0 °C.
