# Corrigendum: Iron-catalysed cross-coupling of organolithium compounds with organic halides

**DOI:** 10.1038/ncomms11955

**Published:** 2016-06-14

**Authors:** Zhenhua Jia, Qiang Liu, Xiao-Shui Peng, Henry N. C. Wong

*Nature Communications* 7: Article number:1061410.1038/ncomms10614 (2016); Published 02052016; Updated 06142016

Previous work by Cahiez *et al.* 2007 describing iron-catalysed cross-coupling of Grignard reagents was inadvertently omitted from the reference list of this Article. This work should have been cited following the sentence ‘This catalytic system developed by Nakamura had been employed in cross-coupling reactions with Grignard reagents and arylzinc reagents'. Additionally, the formation of the TMEDA/iron(III) chloride complex was incorrectly attributed; the statement ‘Therefore, the complex of TMEDA with iron(III) chloride was prepared according to Nakamura's procedure' should read ‘Therefore, the complex of TMEDA with iron(III) chloride was prepared according to Cahiez's procedure (Cahiez *et al.* 2007)'.

Cahiez, G., Habiak, V., Duplais, C. & Moyeux, A. Iron-catalyzed alkylations of aromatic Grignard reagents. *Angew. Chem. Int. Ed.* **46**, 4364–4366 (2007).

