# Supplementary material for: Iron-catalysed cross-coupling of organolithium compounds with organic halides
Source: Nat Commun. 2016 Feb 5;7:10614. doi: 10.1038/ncomms10614 (PMC4748252; doi:10.1038/ncomms10614)
Supplement: Supplementary Information — Supplementary Figures 1-72, Supplementary Tables 1-2, Supplementary Methods and Supplementary References [file ncomms10614-s1.pdf]

Supplementary Figures

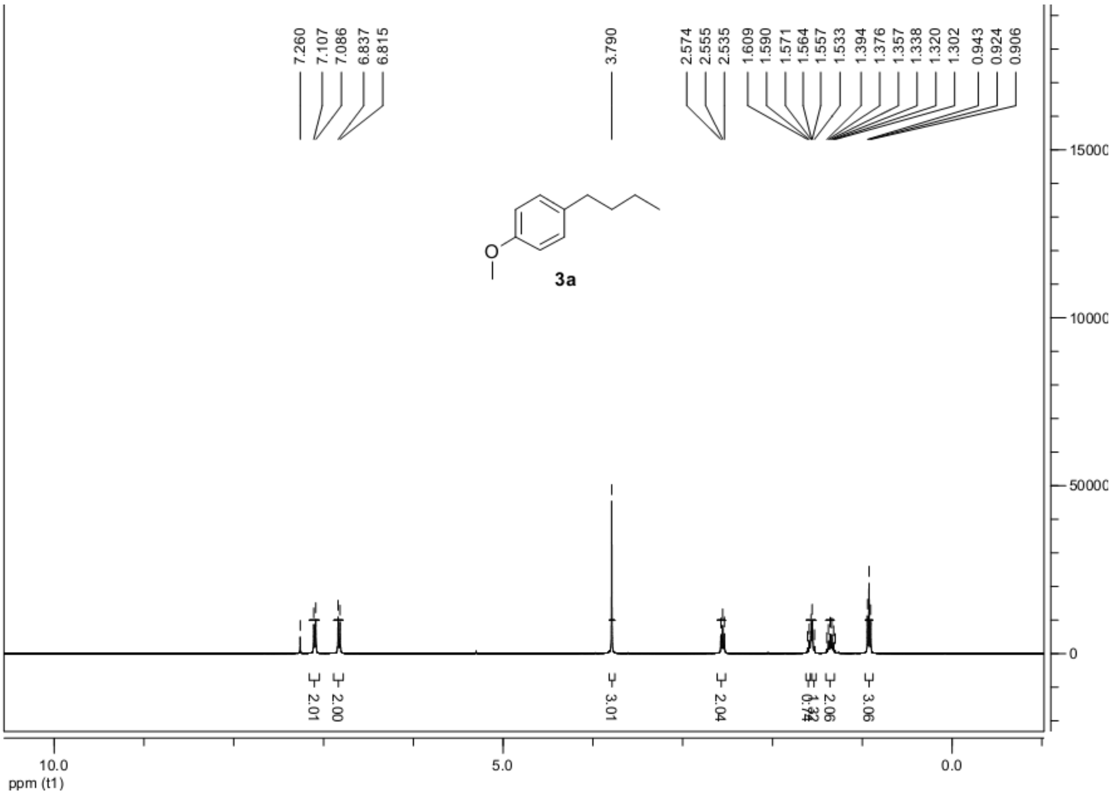

Supplementary Figure 1 | ^{1}H NMR spectrum of 1-butyl-4-methoxybenzene (3a). (400 MHz, $CDCl_3$, 298 K).

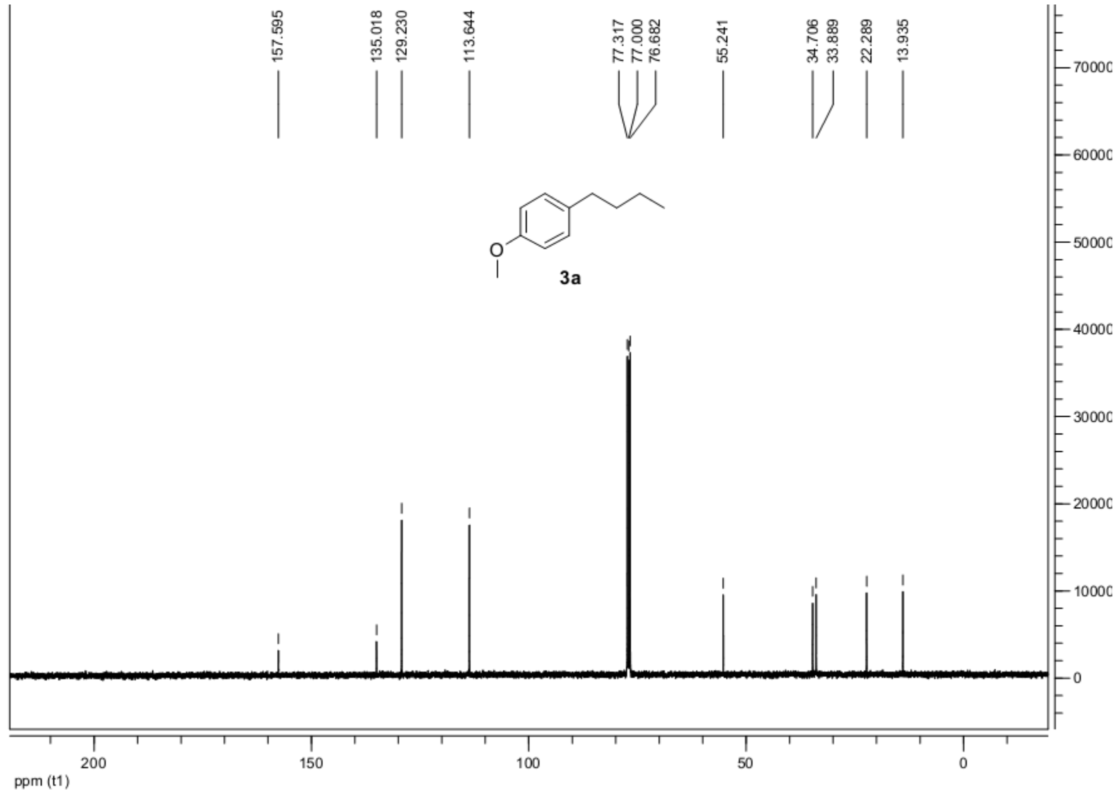

Supplementary Figure 2 | ^{13}C NMR spectrum of 1-butyl-4-methoxybenzene (3a). (100 MHz, $CDCl_3$, 298 K).

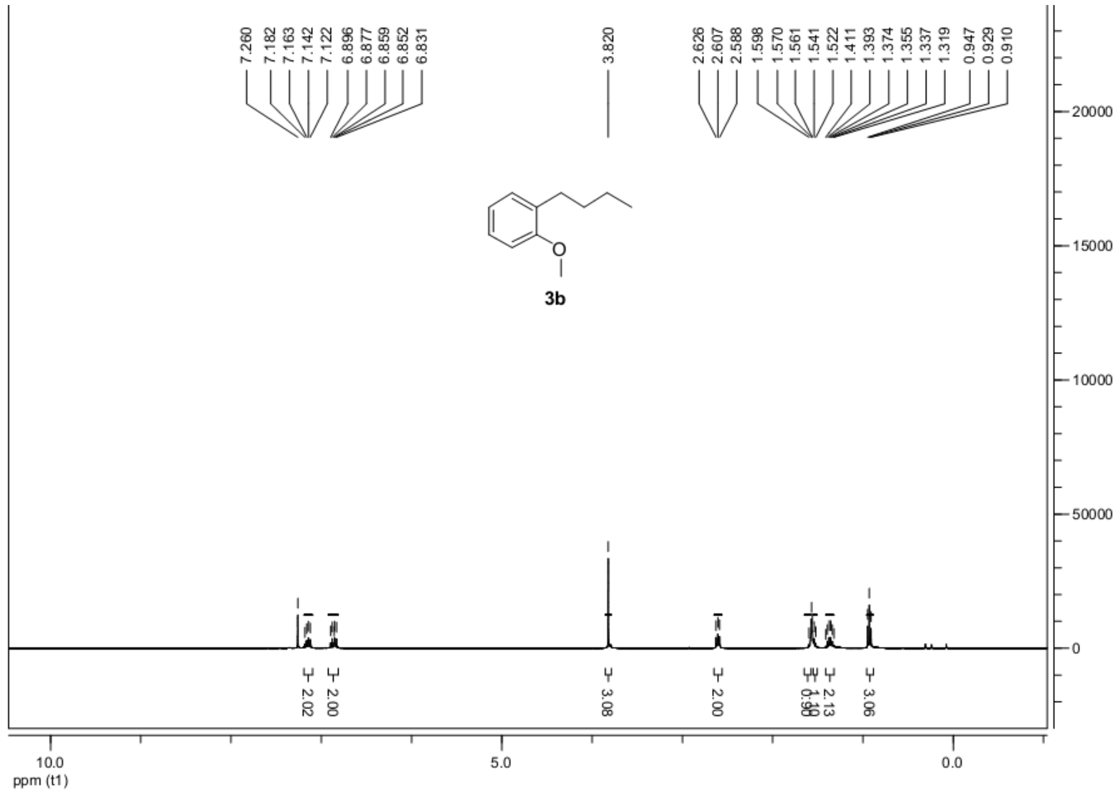

Supplementary Figure 3 | ^{1}H NMR spectrum of 1-butyl-2-methoxybenzene (3b). (400 MHz, $CDCl_3$, 298 K).

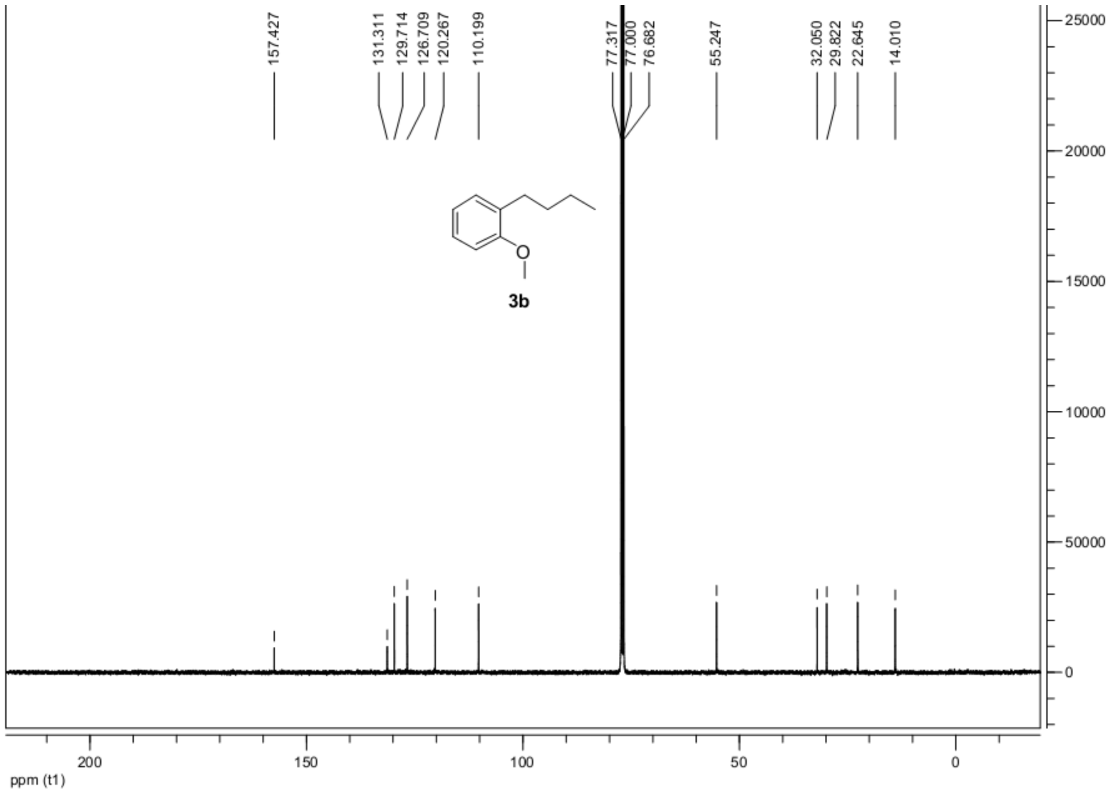

Supplementary Figure 4 | ^{13}C NMR spectrum of 1-butyl-4-methoxybenzene (3b). (100 MHz, $CDCl_3$, 298 K).

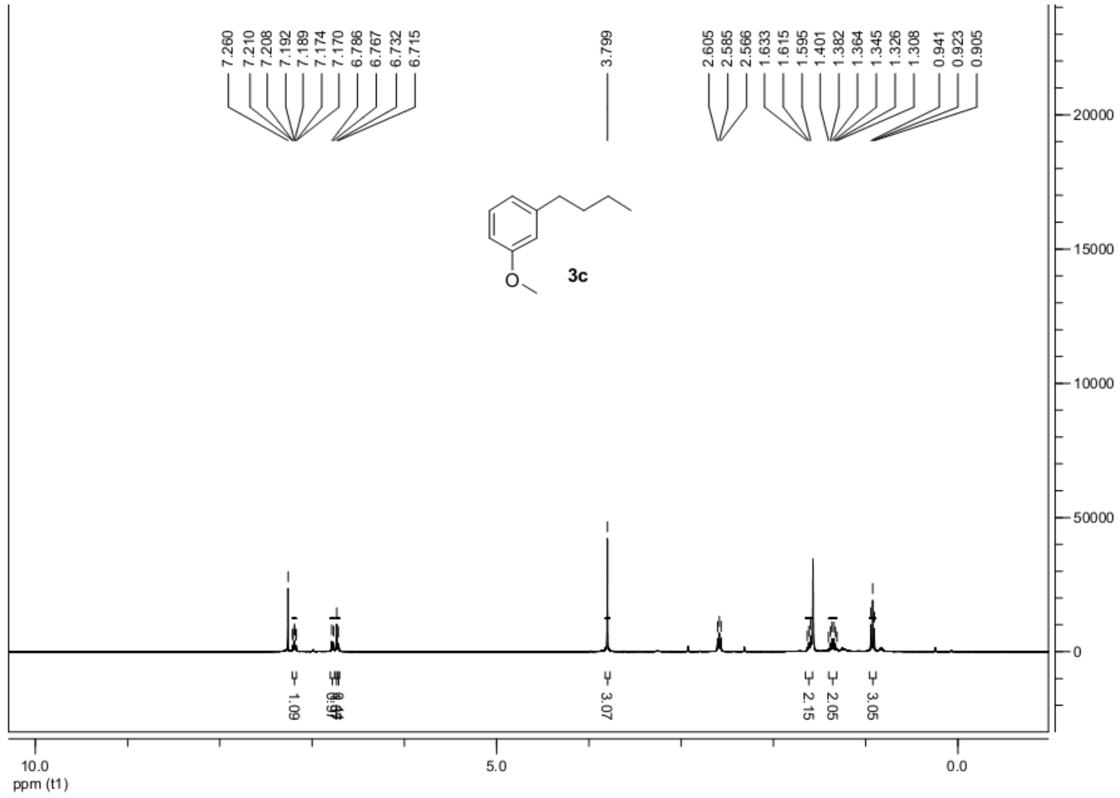

Supplementary Figure 5 | ^{1}H NMR spectrum of 1-butyl-3-methoxybenzene (3c). (400 MHz, $CDCl_3$, 298 K).

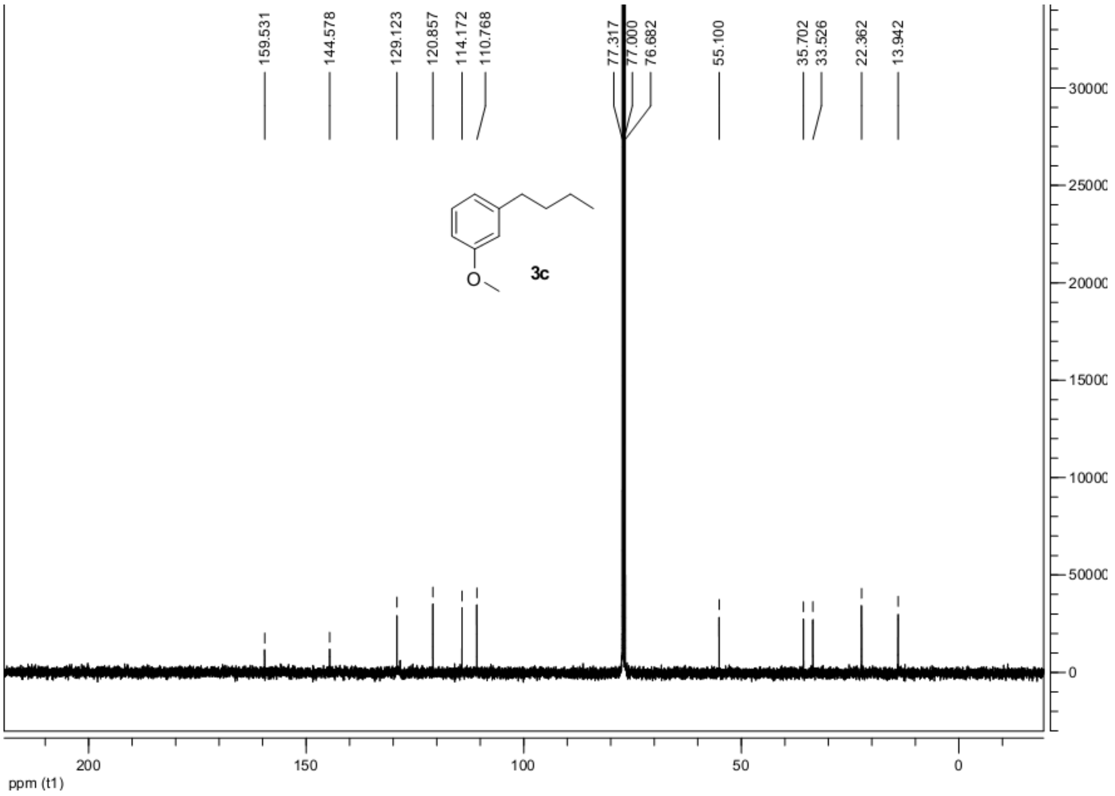

Supplementary Figure 6 | ^{13}C NMR spectrum of 1-butyl-3-methoxybenzene (3c). (100 MHz, $CDCl_3$, 298 K).

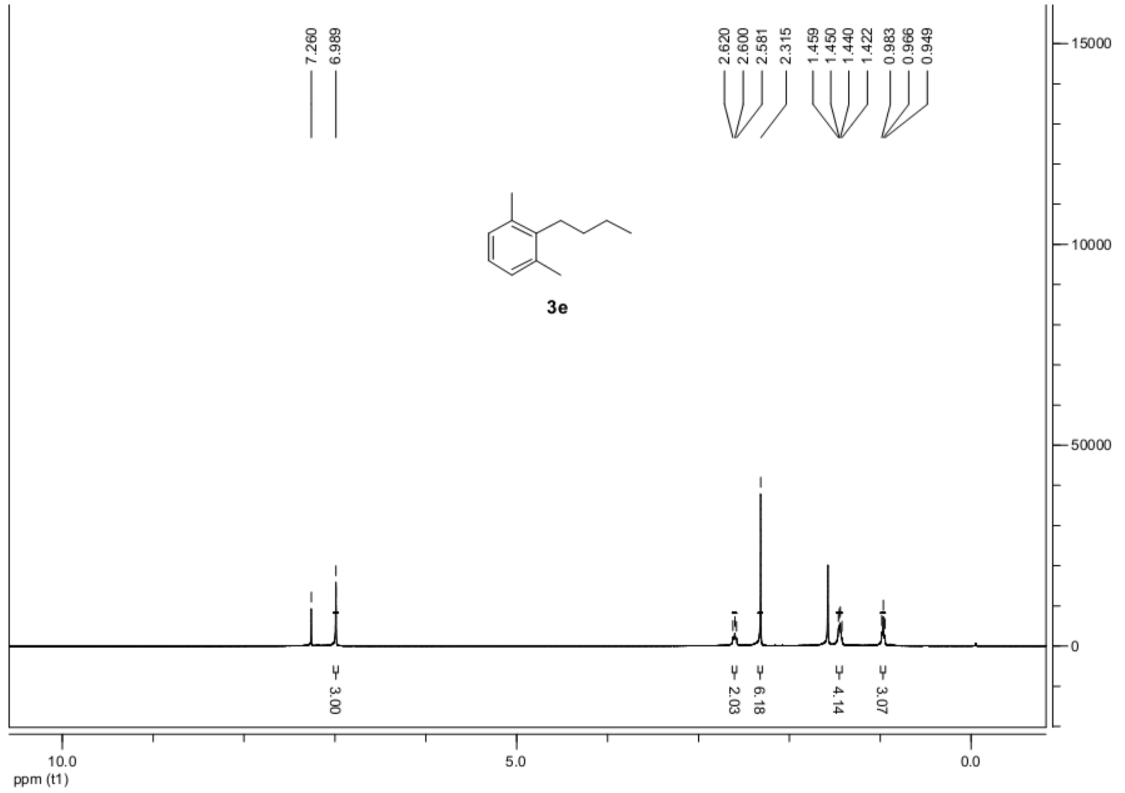

Supplementary Figure 7 | ^{1}H NMR spectrum of 2-butyl-1,3-dimethylbenzene (3e). (400 MHz, $CDCl_3$, 298 K).

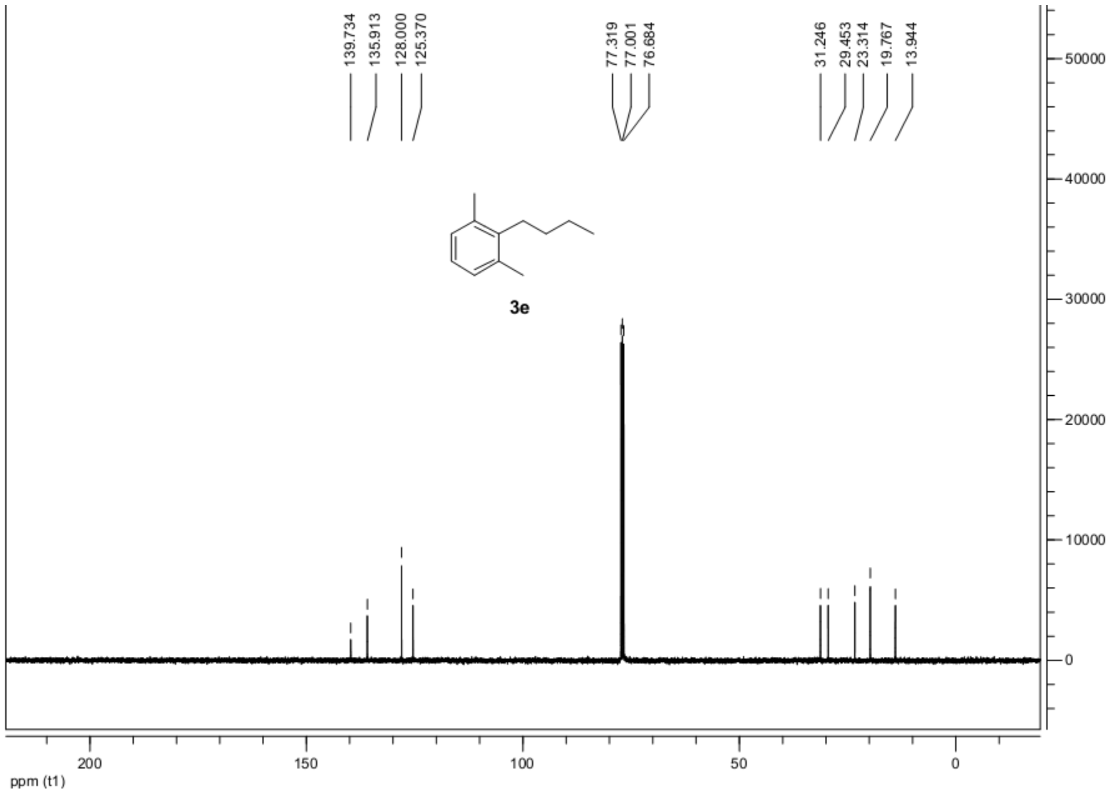

Supplementary Figure 8 | ^{13}C NMR spectrum of 2-butyl-1,3-dimethylbenzene (3e). (100 MHz, $CDCl_3$, 298 K).

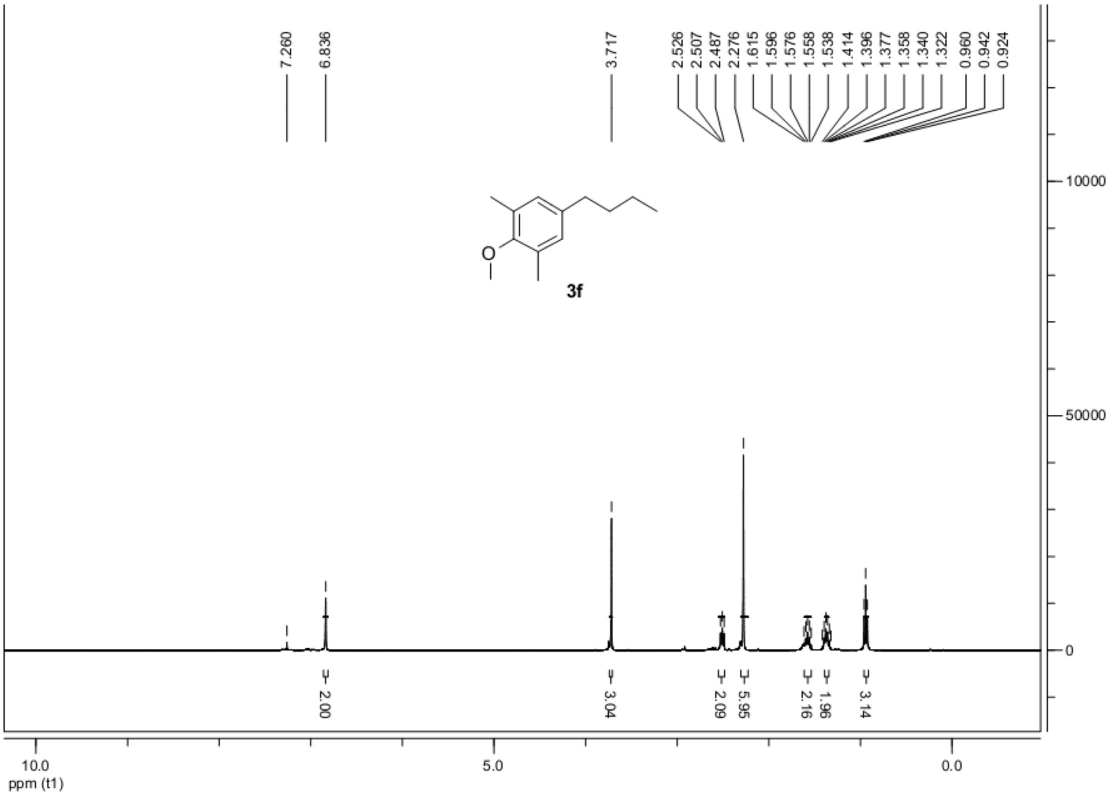

Supplementary Figure 9 | ^{1}H NMR spectrum of 5-butyl-2-methoxy-1,3-dimethylbenzene (3f). (400 MHz, $CDCl_3$, 298 K).

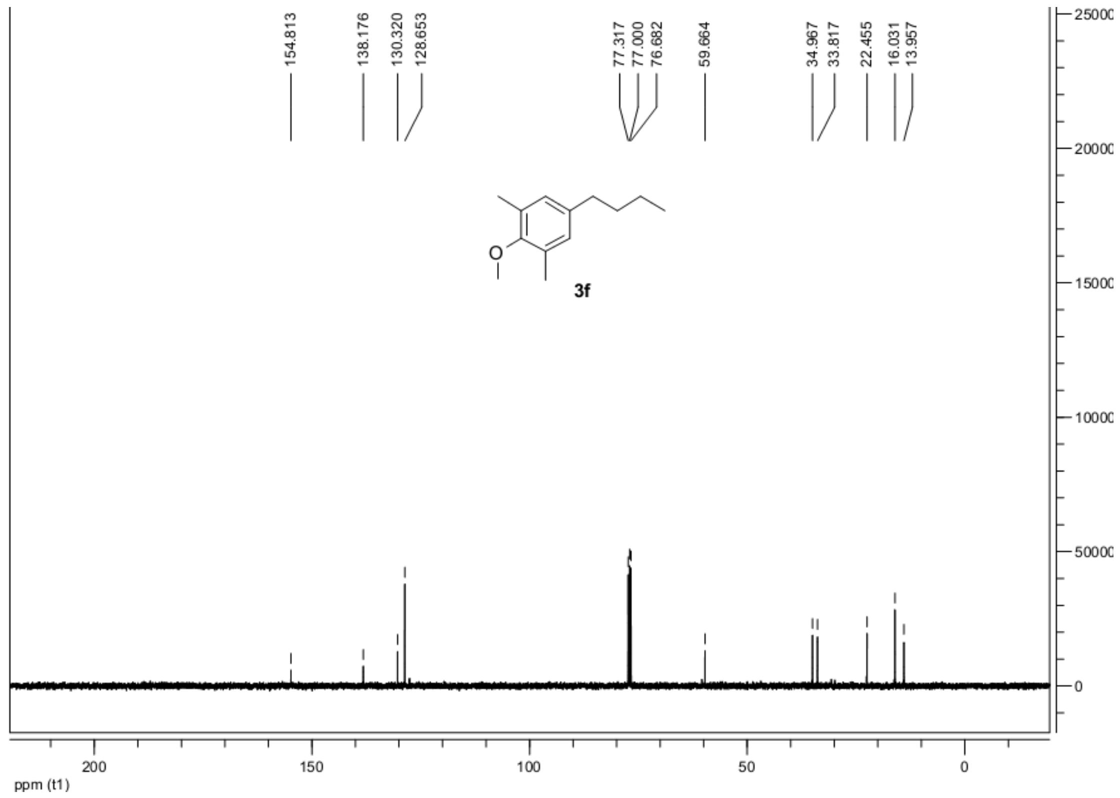

Supplementary Figure 10 | ^{13}C NMR spectrum of 5-butyl-2-methoxy-1,3-dimethylbenzene (3f). (100 MHz, $CDCl_3$, 298 K).

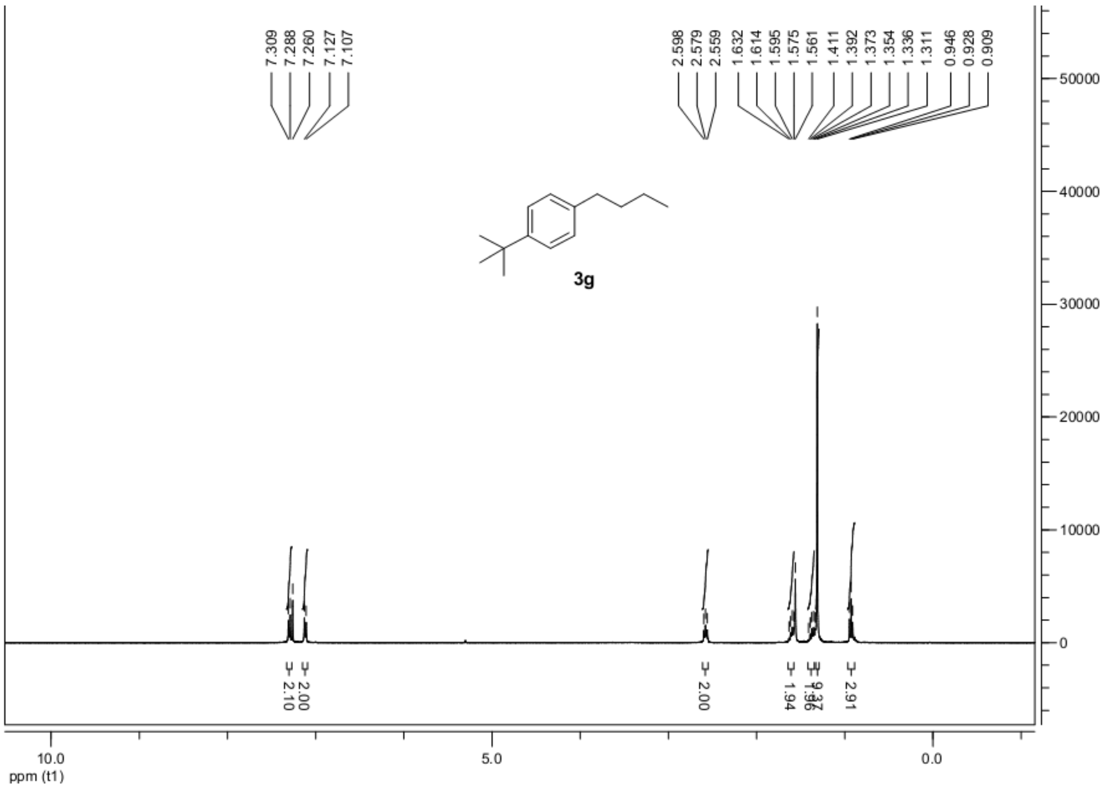

Supplementary Figure 11 | ^{1}H NMR spectrum of 1-*tert*-butyl-4-butylbenzene (3g). (400 MHz, $CDCl_3$, 298 K).

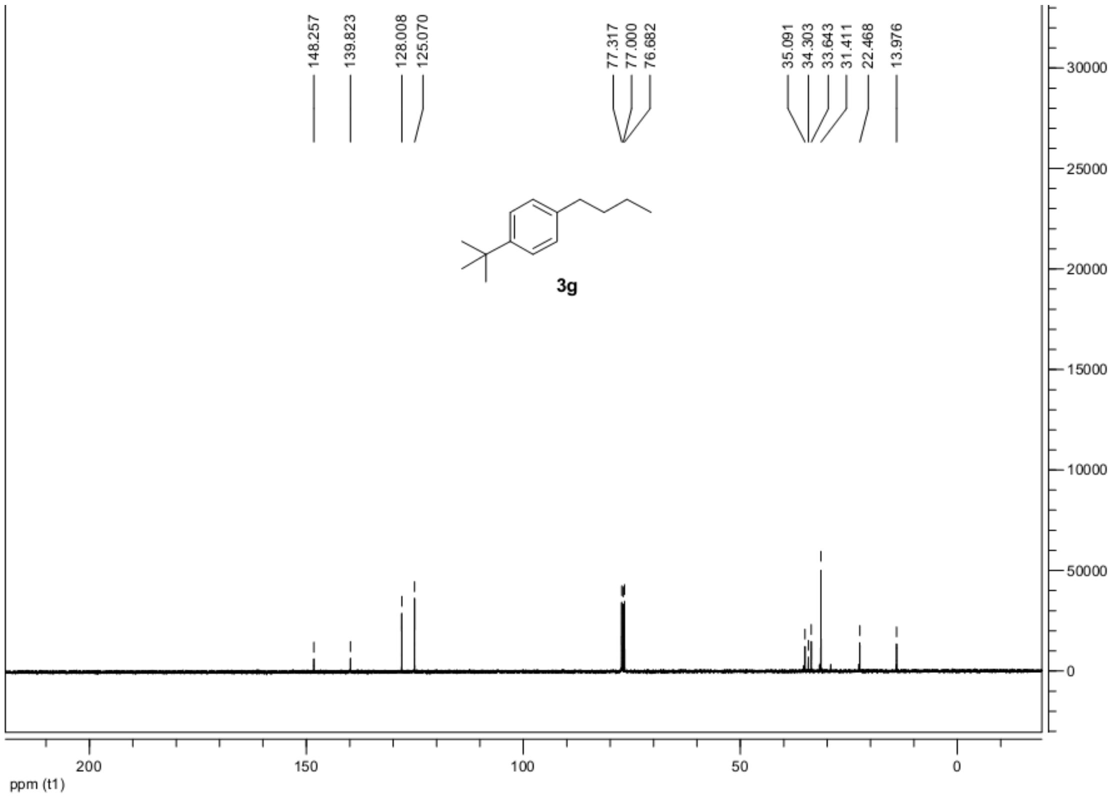

Supplementary Figure 12 | ^{13}C NMR spectrum of 1-*tert*-butyl-4-butylbenzene (3g). (100 MHz, $CDCl_3$, 298 K).

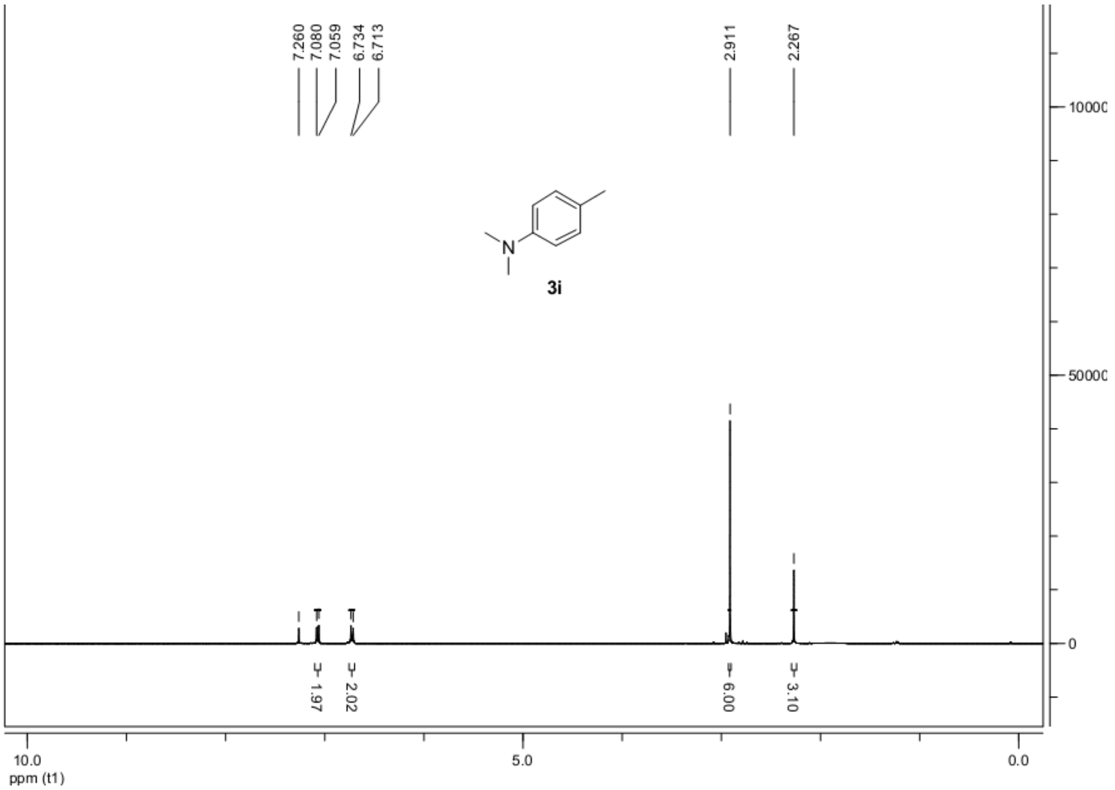

Supplementary Figure 13 | ^{1}H NMR spectrum of dimethyl-*p*-toluidine (3i). (400 MHz, $CDCl_3$, 298 K).

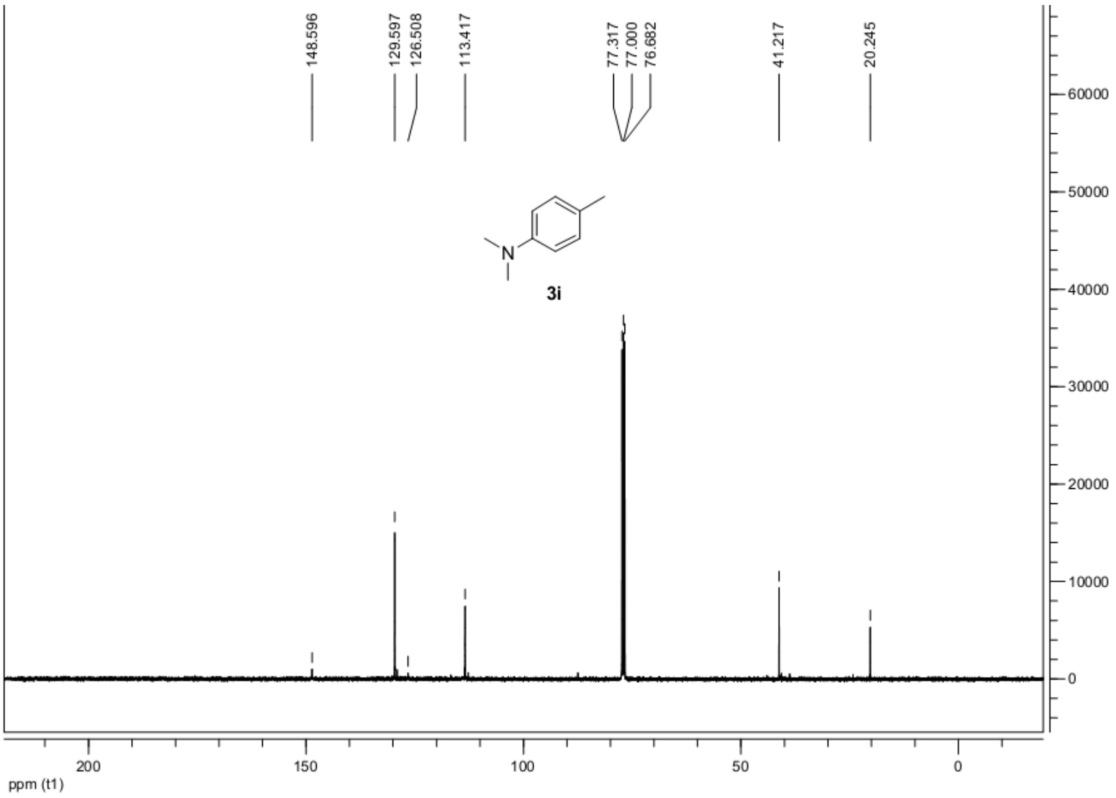

Supplementary Figure 14 | ^{13}C NMR spectrum of dimethyl-*p*-toluidine (3i). (100 MHz, $CDCl_3$, 298 K).

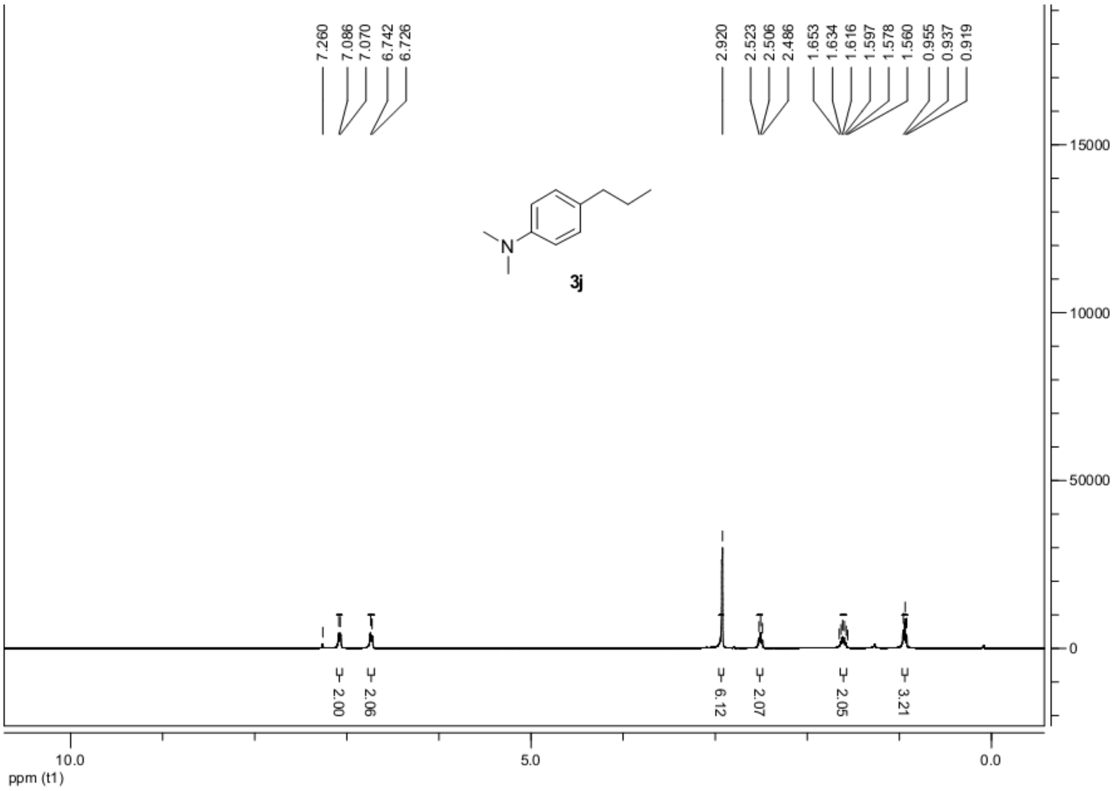

Supplementary Figure 15 | ^{1}H NMR spectrum of *N*,*N*-dimethyl-4-propylaniline (3j). (400 MHz, $CDCl_3$, 298 K).

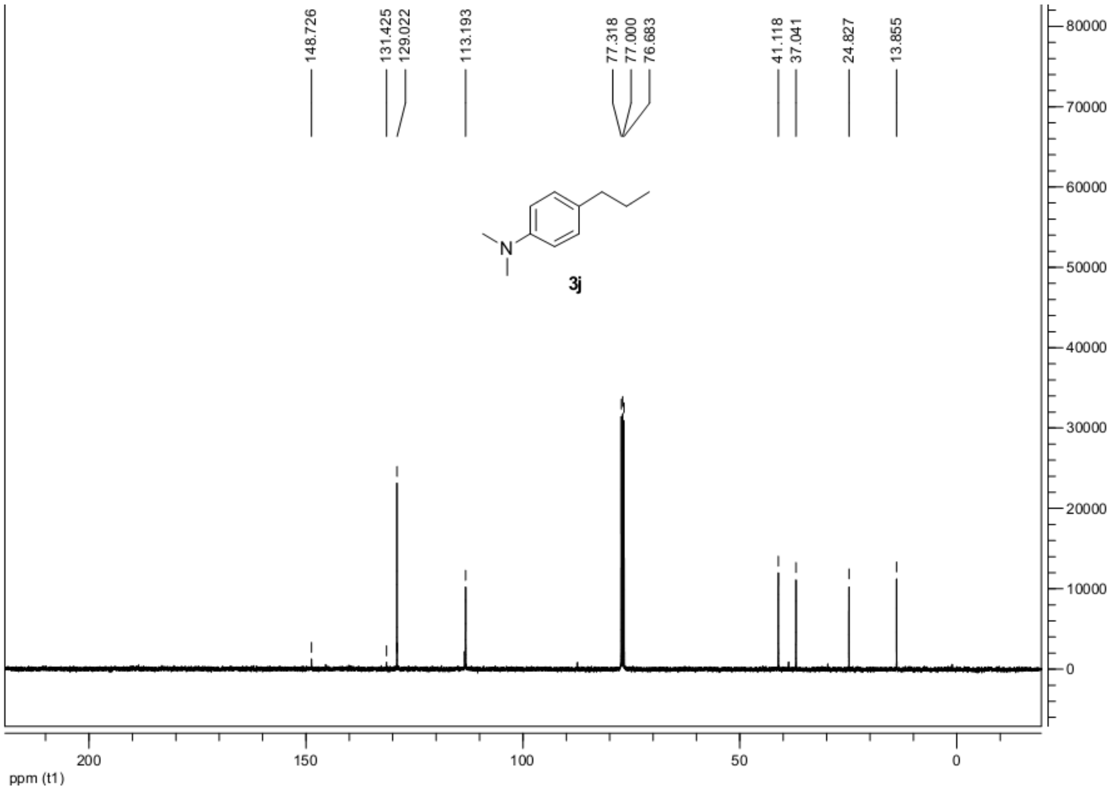

Supplementary Figure 16 | ^{13}C NMR spectrum of *N*,*N*-dimethyl-4-propylaniline (3j). (100 MHz, $CDCl_3$, 298 K).

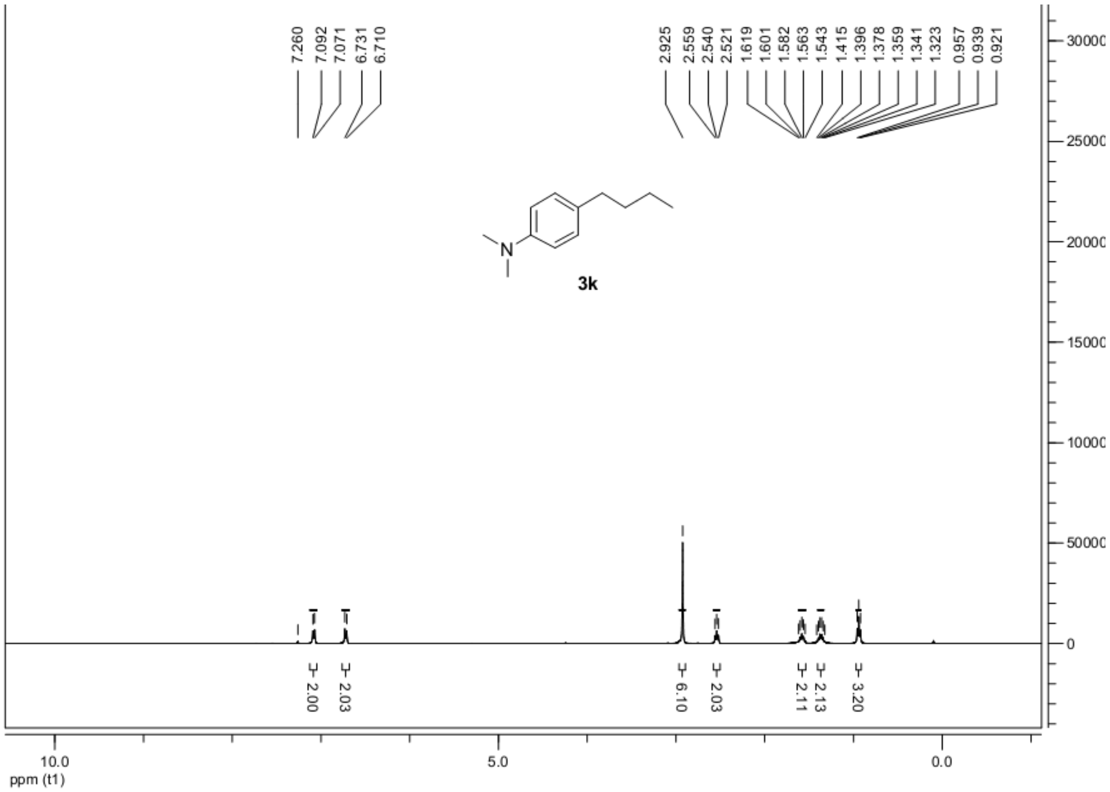

Supplementary Figure 17 | ^{1}H NMR spectrum of 4-butyl-*N*,*N*-dimethylaniline (3k). (400 MHz, $CDCl_3$, 298 K).

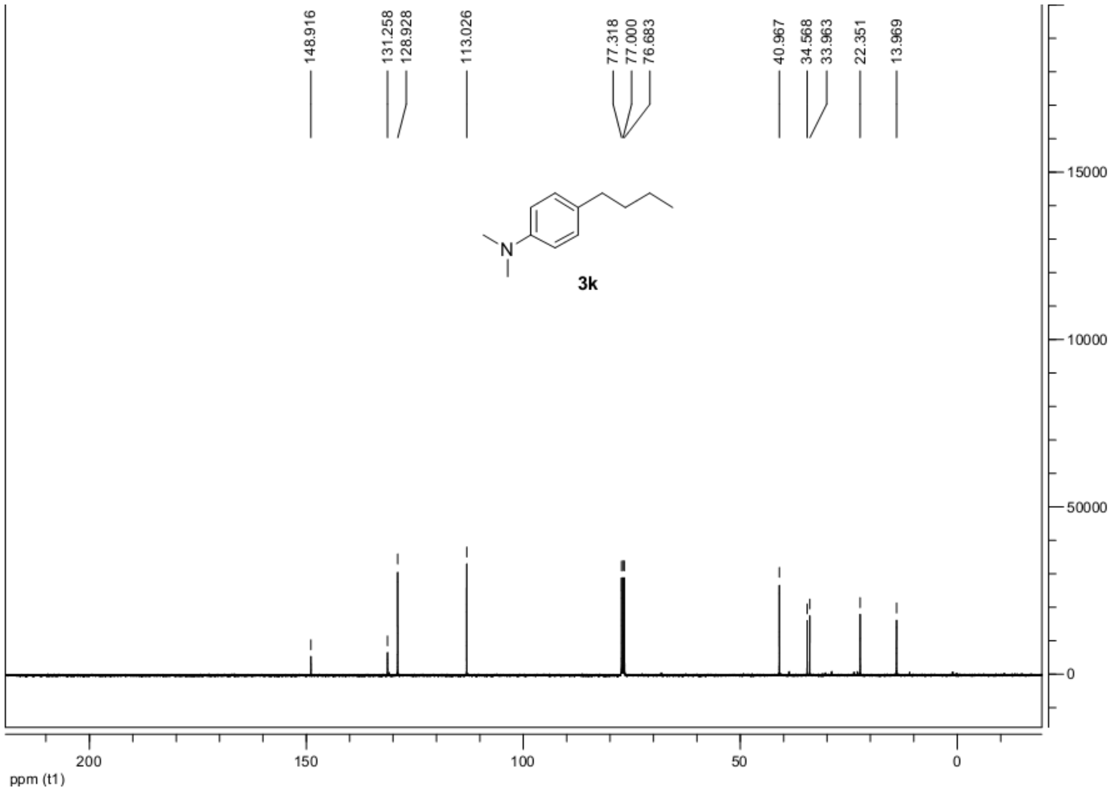

Supplementary Figure 18 | ^{13}C NMR spectrum of 4-butyl-*N*,*N*-dimethylaniline (3k). (100 MHz, $CDCl_3$, 298 K).

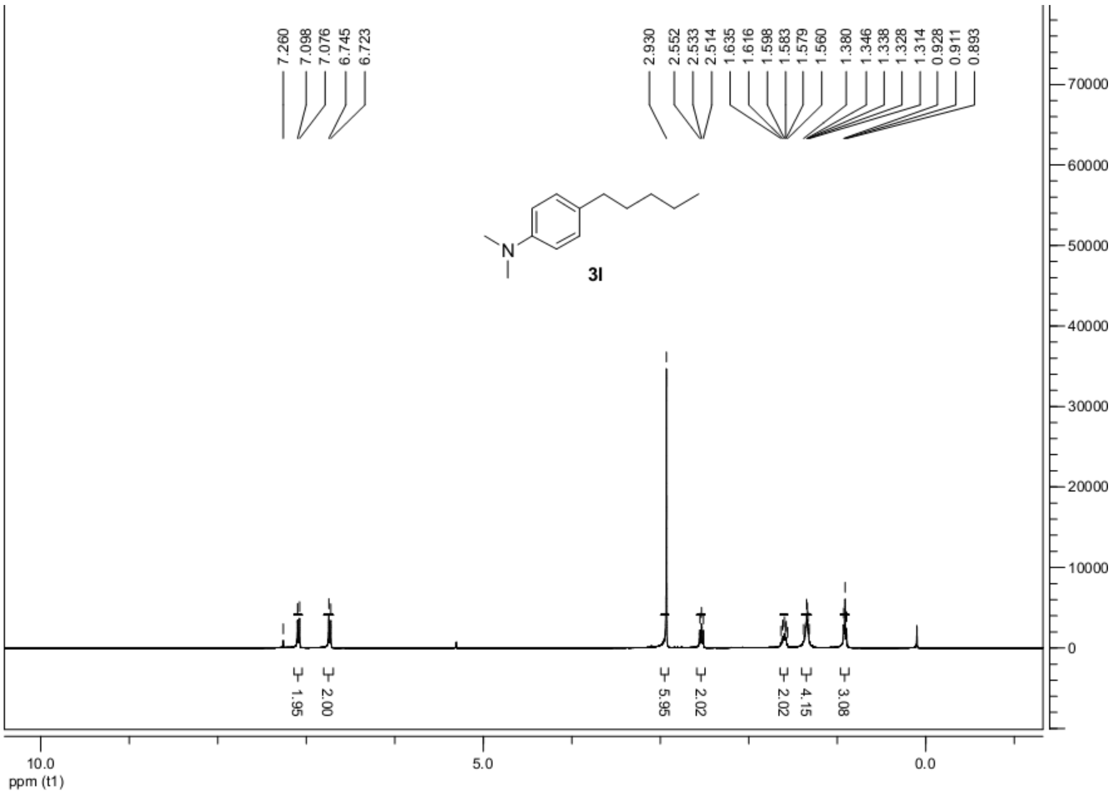

Supplementary Figure 19 | ^{1}H NMR spectrum of *N,N*-dimethyl-4-pentylaniline (3l). (400 MHz, $CDCl_3$, 298 K).

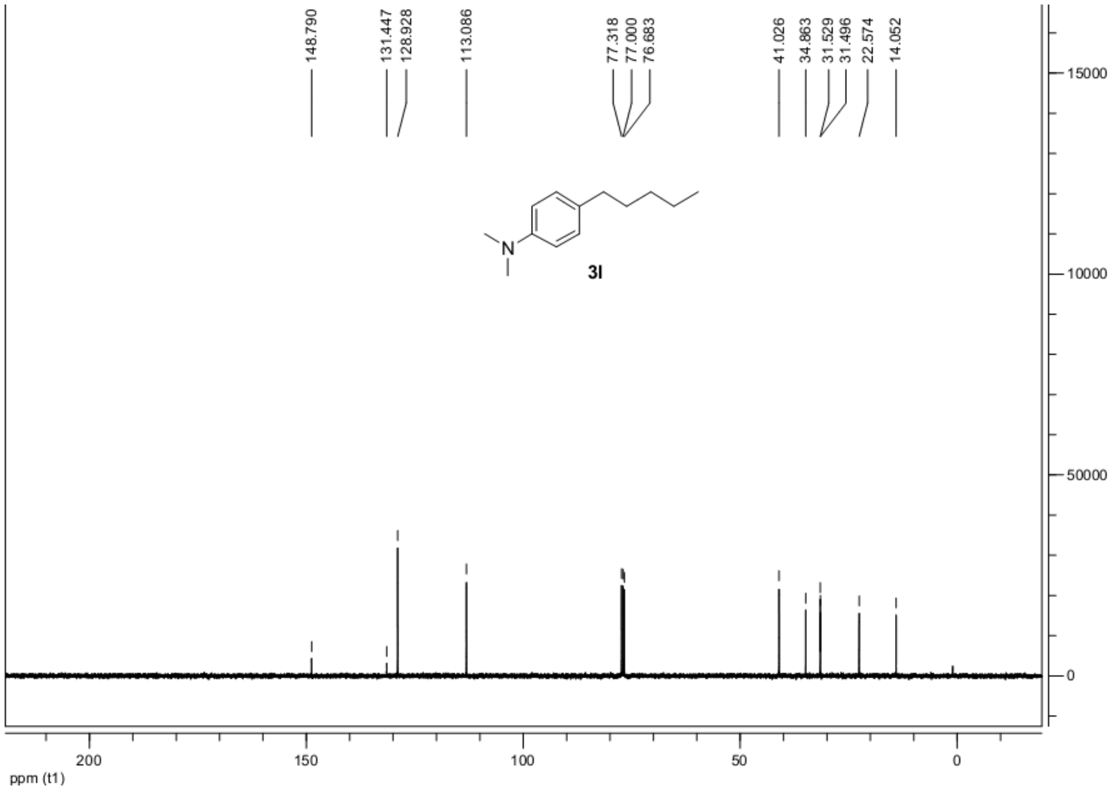

Supplementary Figure 20 | ^{13}C NMR spectrum of *N,N*-dimethyl-4-pentylaniline (3l). (100 MHz, $CDCl_3$, 298 K).

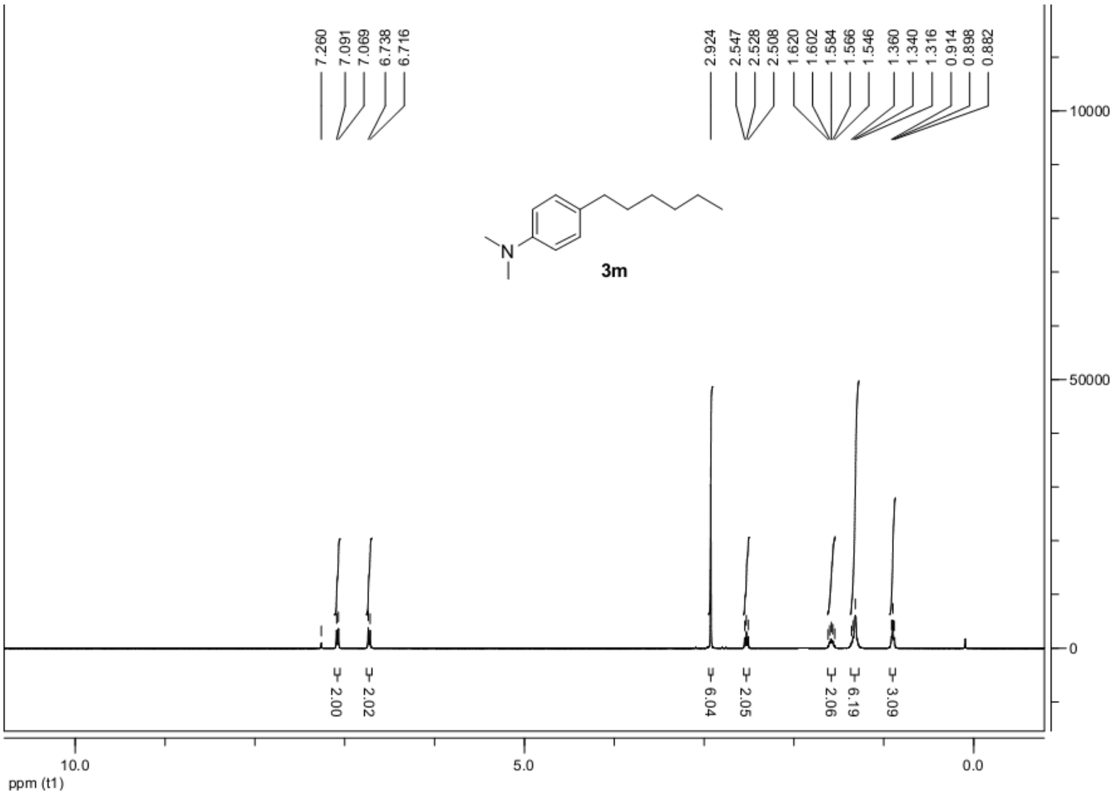

Supplementary Figure 21 | ^{1}H NMR spectrum of 4-hexyl-*N*,*N*-dimethylaniline (3m). (400 MHz, $CDCl_3$, 298 K).

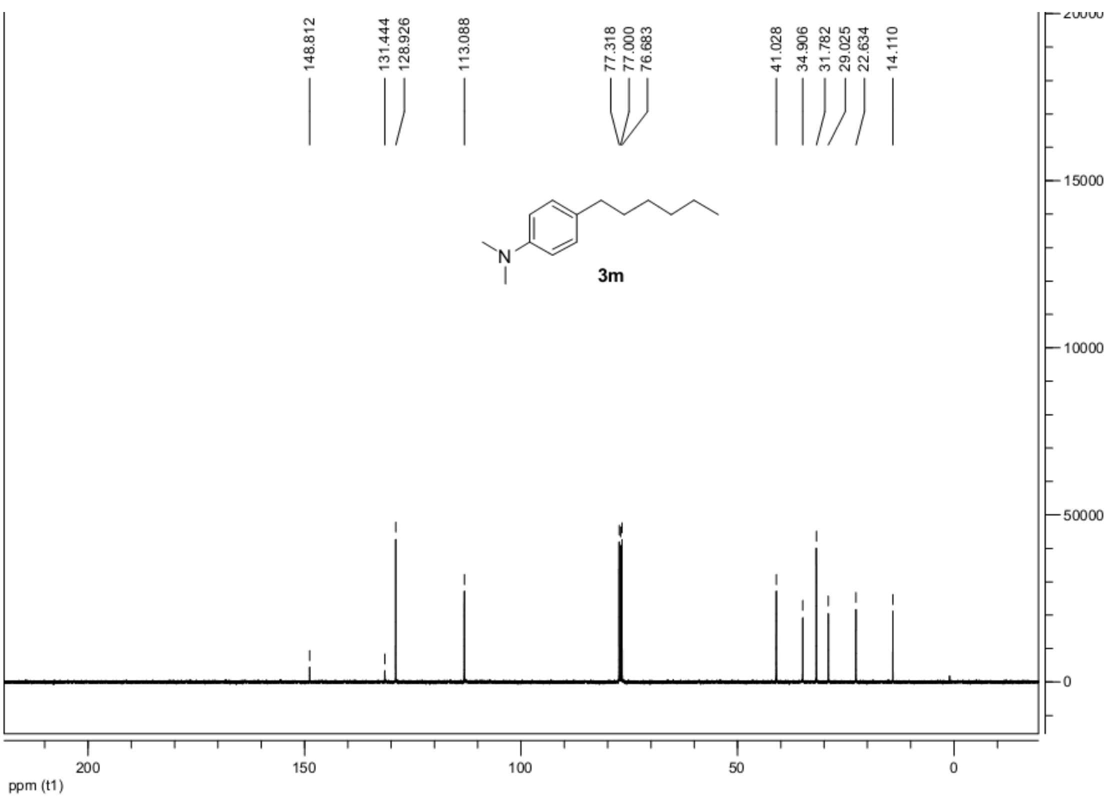

Supplementary Figure 22 | ^{13}C NMR spectrum of 4-hexyl-*N*,*N*-dimethylaniline (3m). (100 MHz, $CDCl_3$, 298 K).

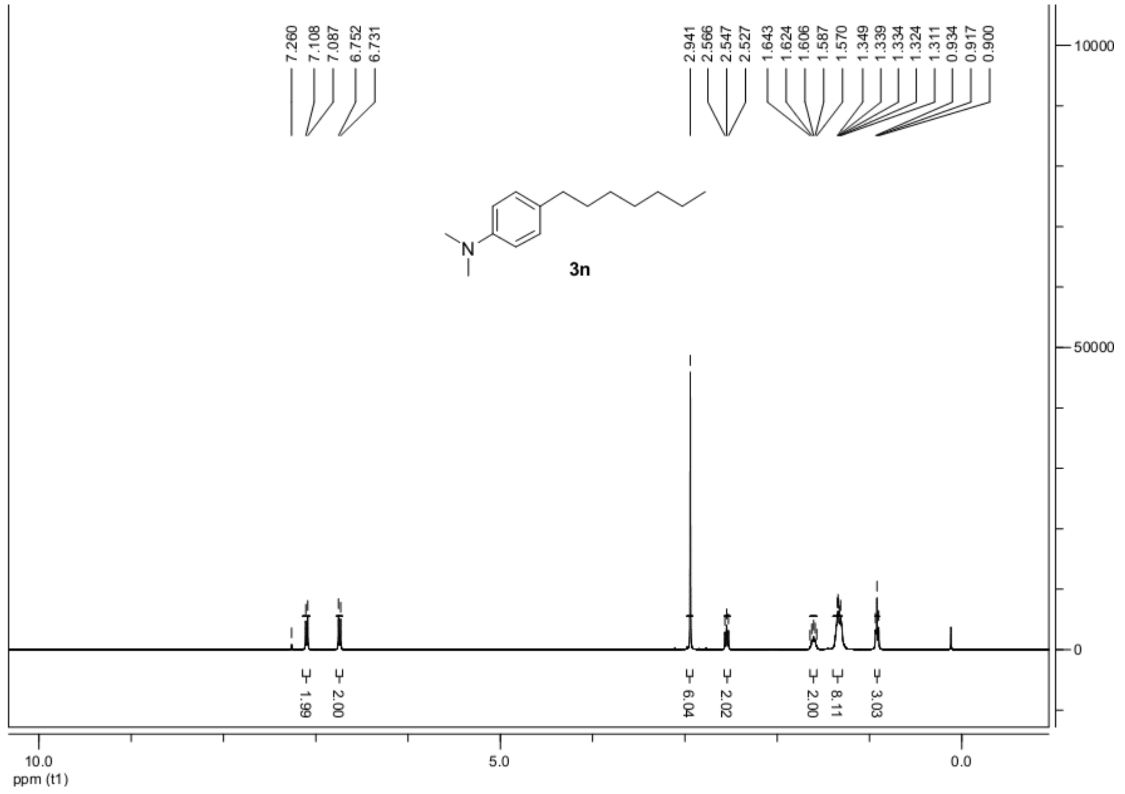

Supplementary Figure 23 | ^{1}H NMR spectrum of 4-heptyl-*N*,*N*-dimethylaniline (3n). (400 MHz, $CDCl_3$, 298 K).

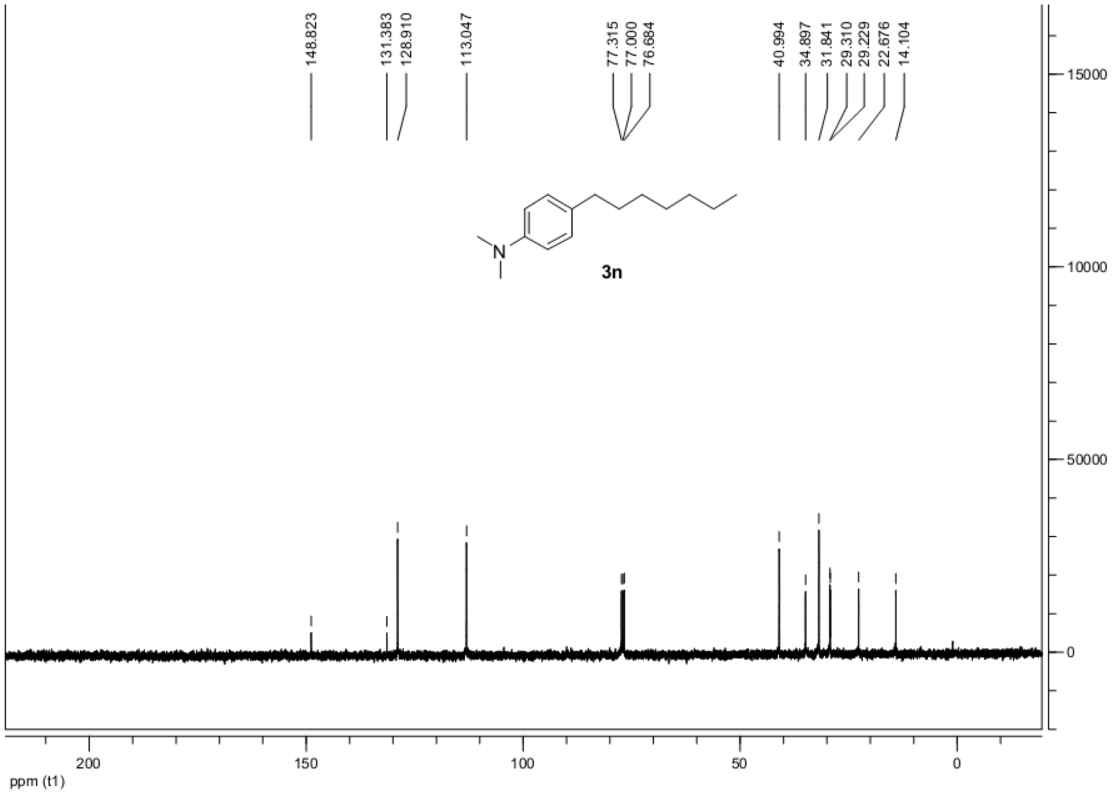

Supplementary Figure 24 | ^{13}C NMR spectrum of 4-heptyl-*N*,*N*-dimethylaniline (3n). (100 MHz, $CDCl_3$, 298 K).

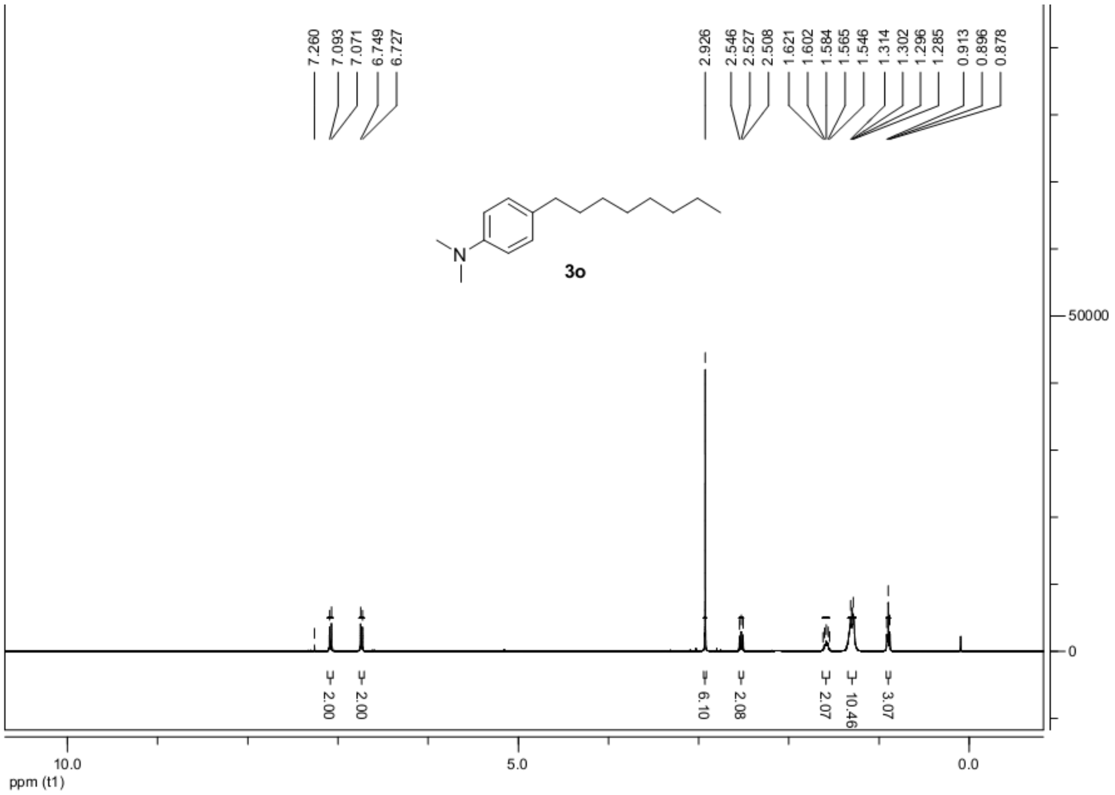

Supplementary Figure 25 | ^{1}H NMR spectrum of *N*,*N*-dimethyl-4-octylaniline (3o). (400 MHz, $CDCl_3$, 298 K).

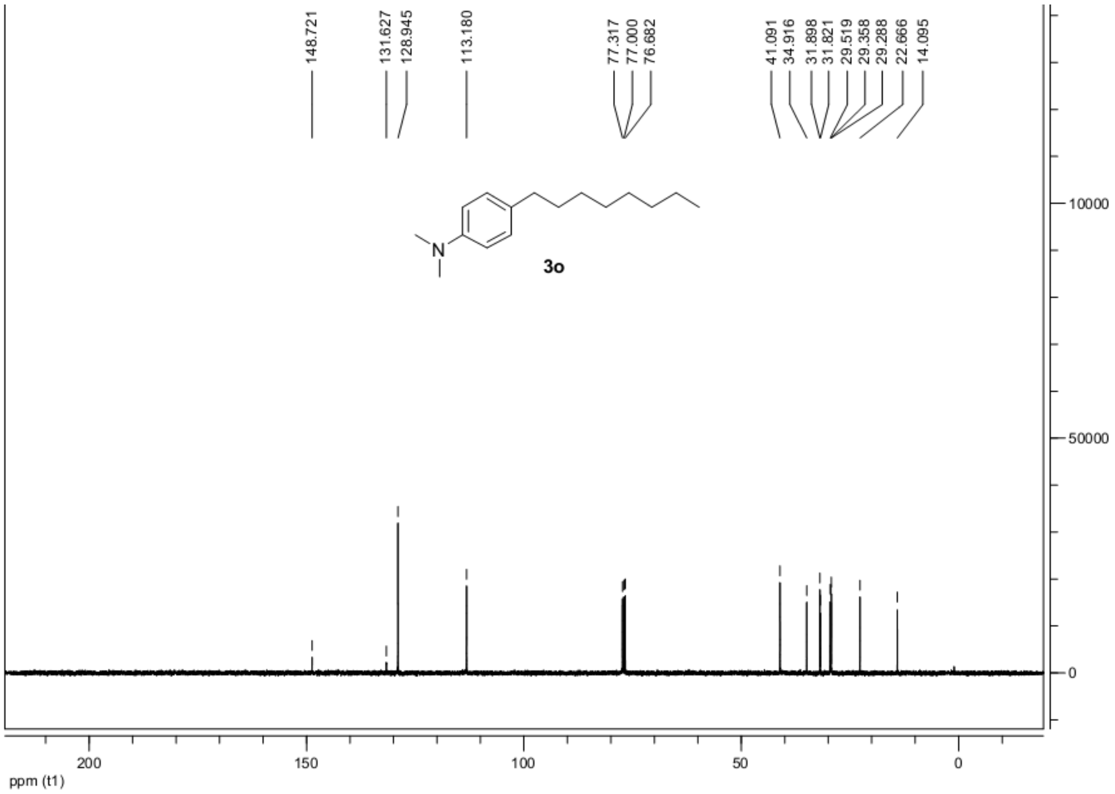

Supplementary Figure 26 | ^{13}C NMR spectrum of *N*,*N*-dimethyl-4-octylaniline (3o). (100 MHz, $CDCl_3$, 298 K).

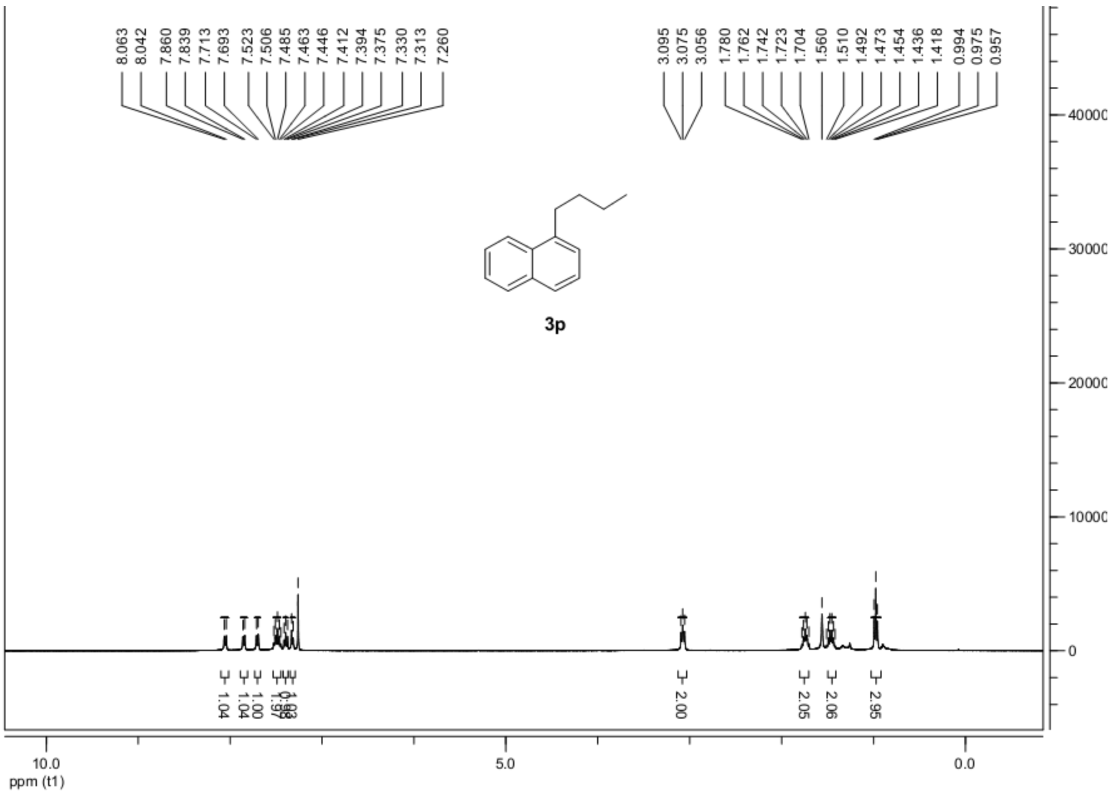

Supplementary Figure 27 | ^{1}H NMR spectrum of 1-butylnaphthalene (3p). (400 MHz, $CDCl_3$, 298 K).

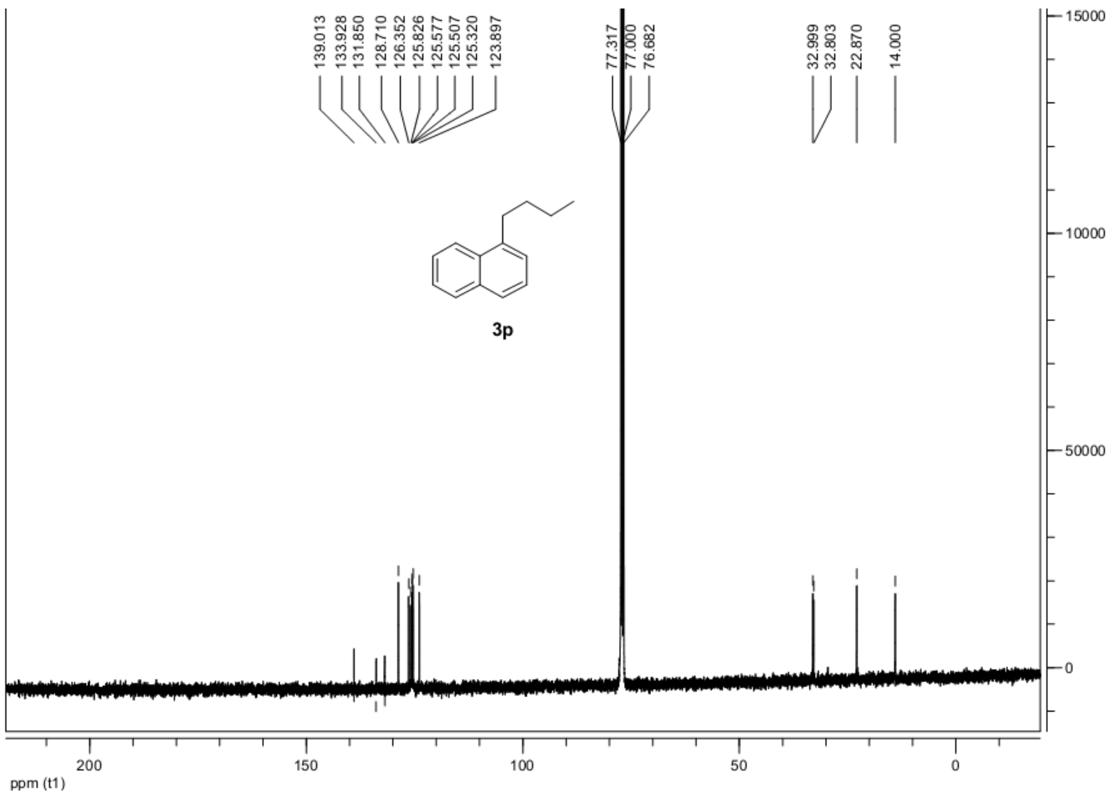

Supplementary Figure 28 | ^{13}C NMR spectrum of 1-butylnaphthalene (3p). (100 MHz, $CDCl_3$, 298 K).

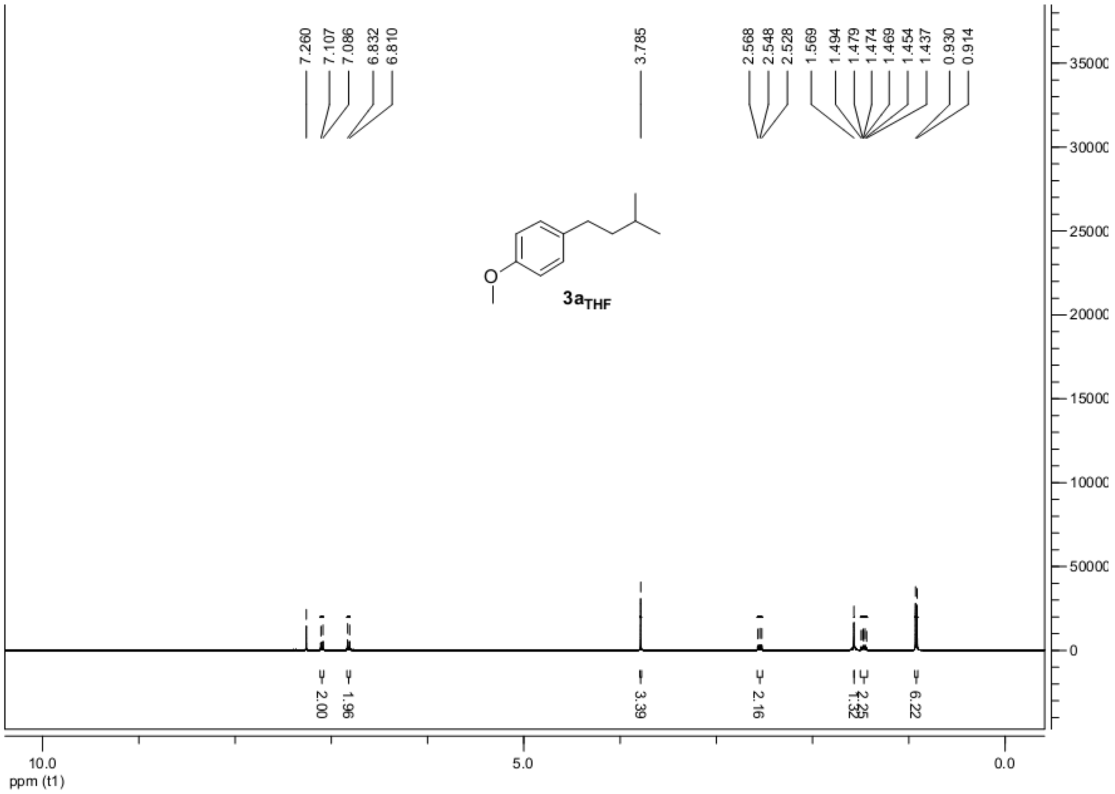

Supplementary Figure 29 | ^{1}H NMR spectrum of 1-isopentyl-4-methoxybenzene ($3a_{THF}$). (400 MHz, $CDCl_3$, 298 K).

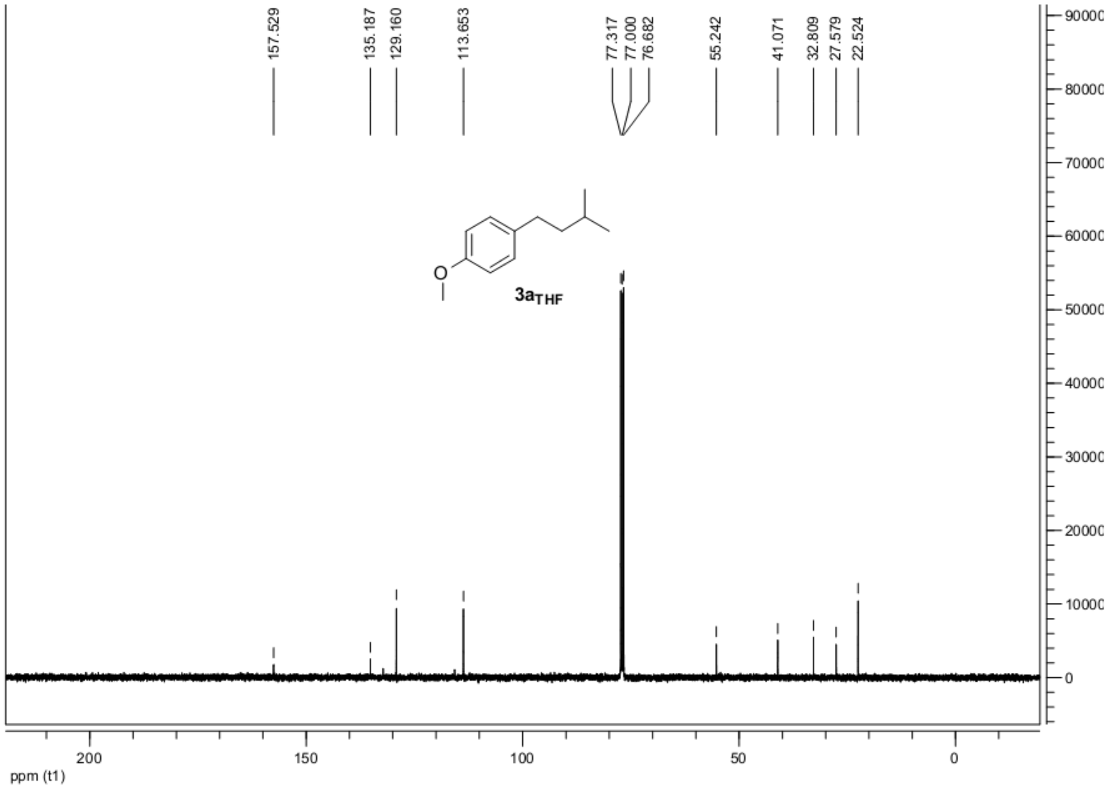

Supplementary Figure 30 | ^{13}C NMR spectrum of 1-isopentyl-4-methoxybenzene ($3a_{THF}$). (100 MHz, $CDCl_3$, 298 K).

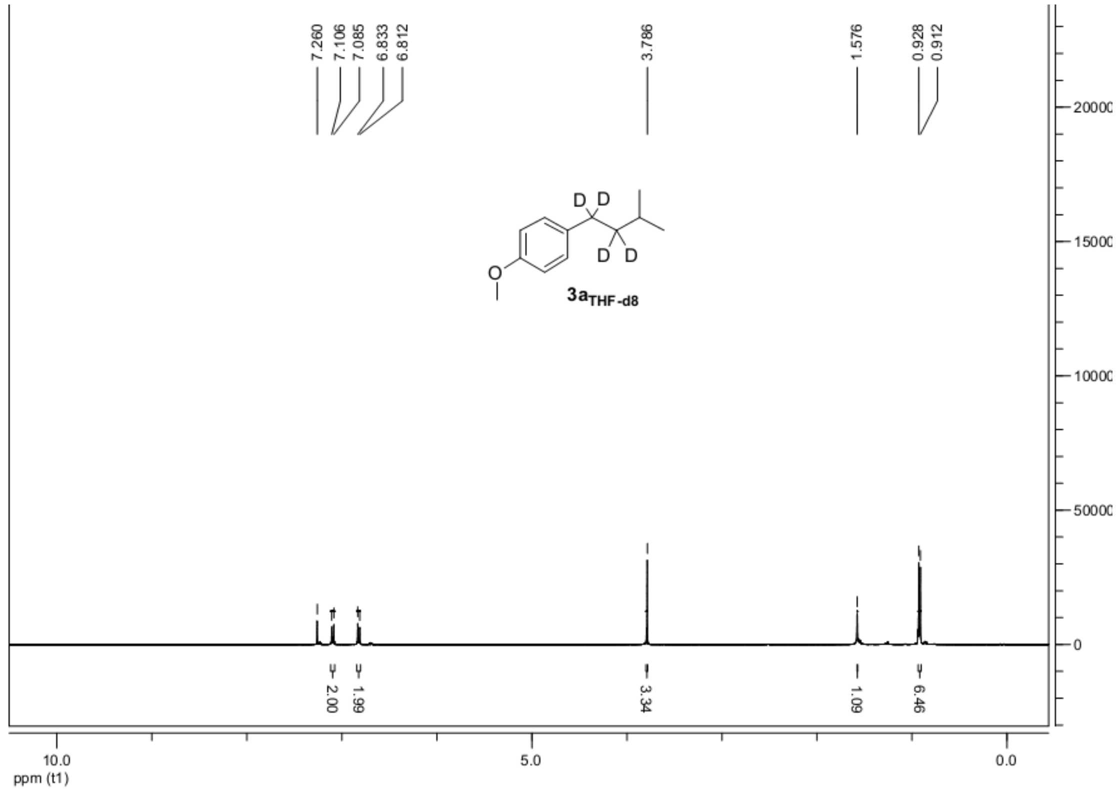

Supplementary Figure 31 | ^{1}H NMR spectrum of 1-methoxy-4-(3-methylbutyl-1,1,2,2-*d*$_4$)benzene (3a$_{THF-d8}$). (400 MHz, $CDCl_3$, 298 K).

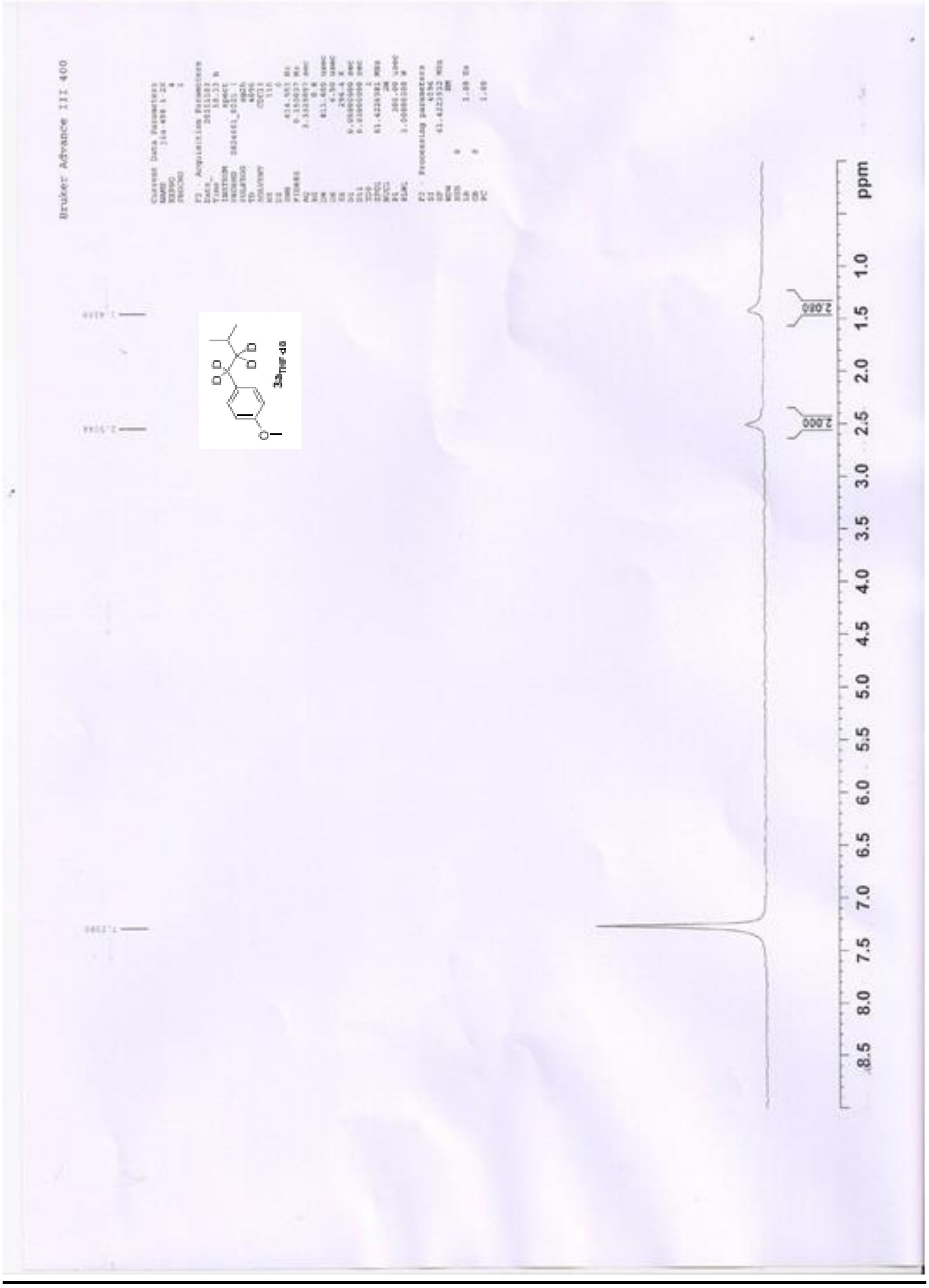

Supplementary Figure 32 | ^{2}H NMR spectrum of 1-methoxy-4-(3-methylbutyl-1,1,2,2-d_4)benzene (3a$_{THF-d8}$). (400 MHz, $CHCl_3$, 298 K).

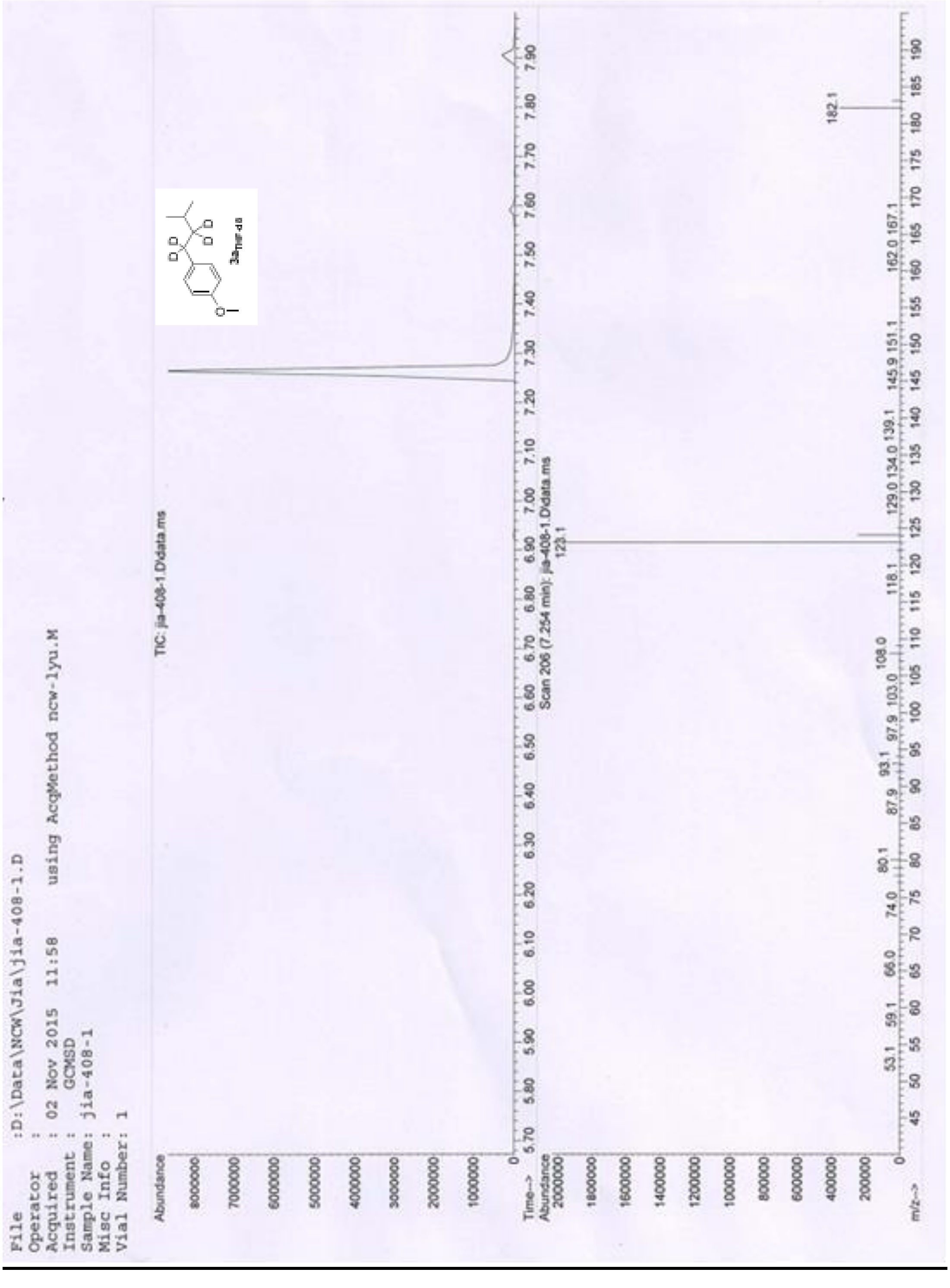

Supplementary Figure 33 | GC-MS spectrum of 1-methoxy-4-(3-methylbutyl-1,1,2,2-*d*$_4$)benzene (3a$_{THF\text{-}d8}$).

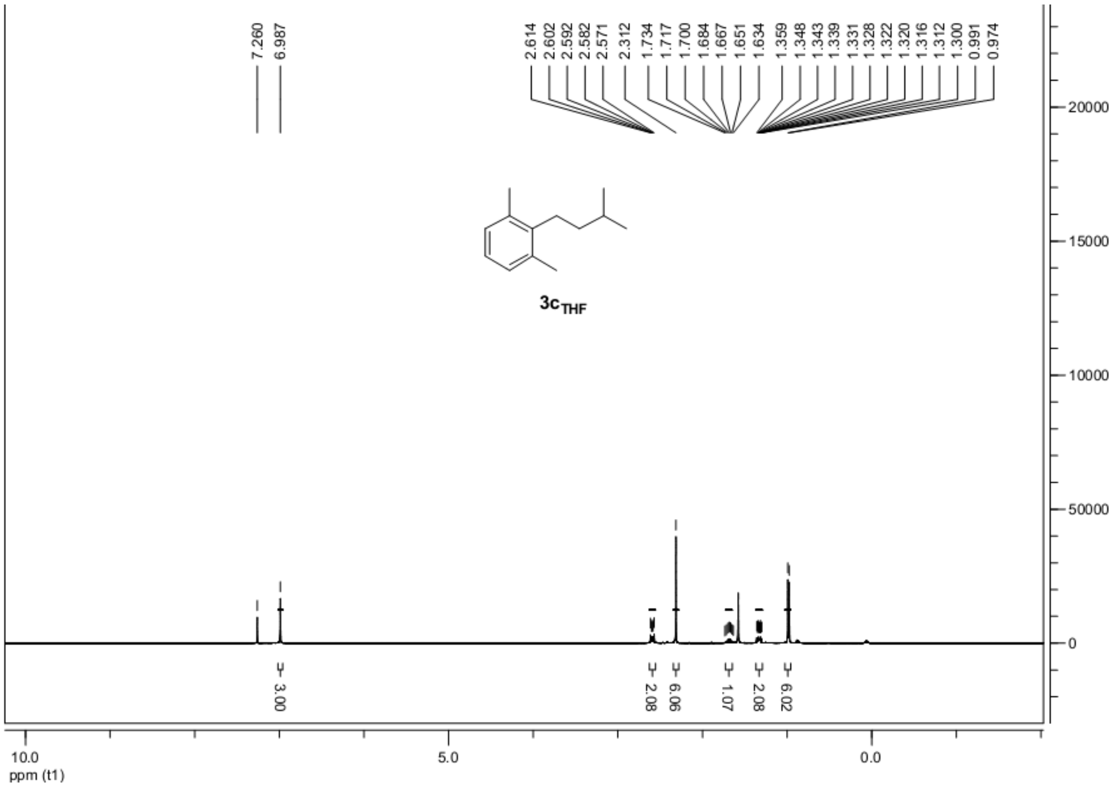

Supplementary Figure 34 | ^{1}H NMR spectrum of 2-Isopentyl-1,3-dimethylbenzene (3c$_{THF}$). (400 MHz, $CDCl_3$, 298 K).

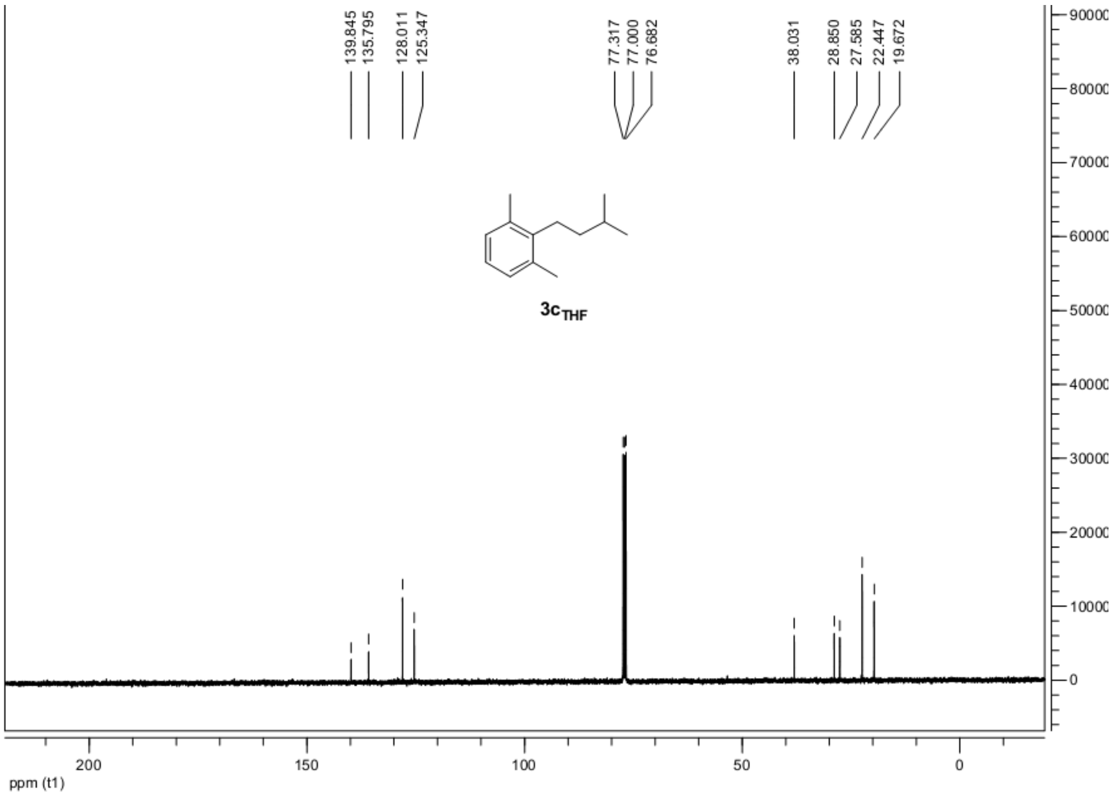

Supplementary Figure 35 | ^{13}C NMR spectrum of 2-Isopentyl-1,3-dimethylbenzene (3c$_{THF}$). (100 MHz, $CDCl_3$, 298 K).

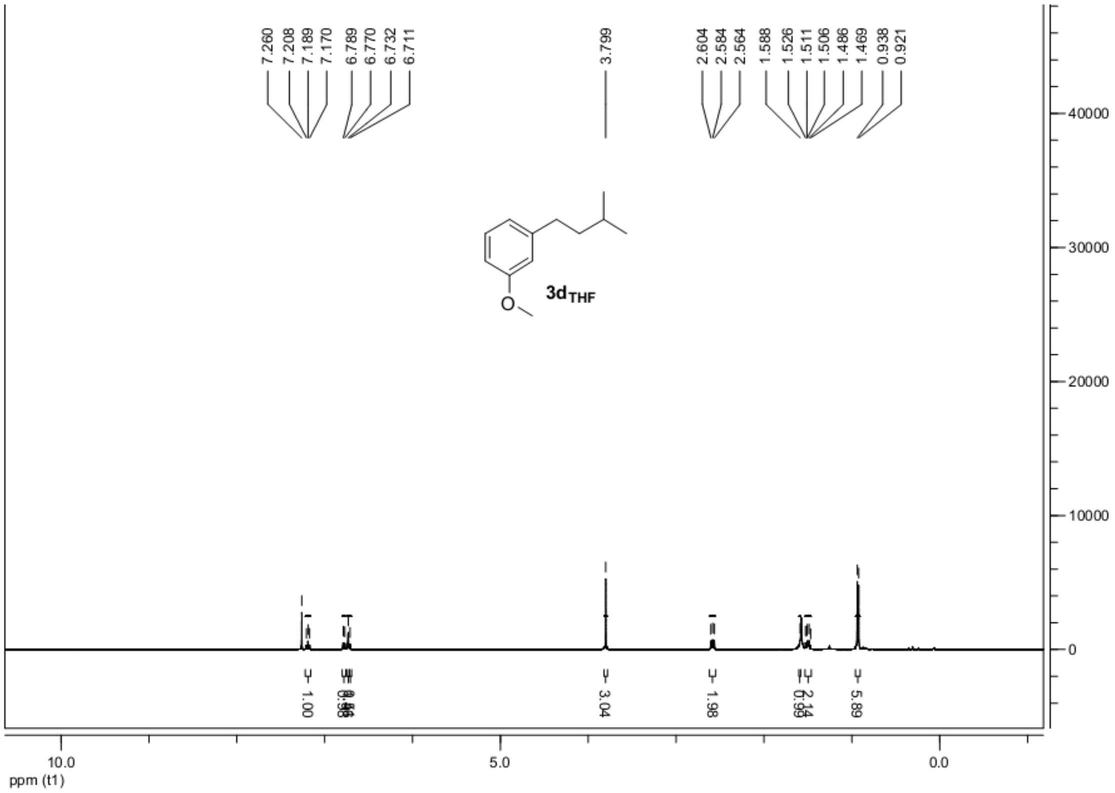

Supplementary Figure 36 | ^{1}H NMR spectrum of 1-isopentyl-3-methoxybenzene (3d$_{THF}$). (400 MHz, $CDCl_3$, 298 K).

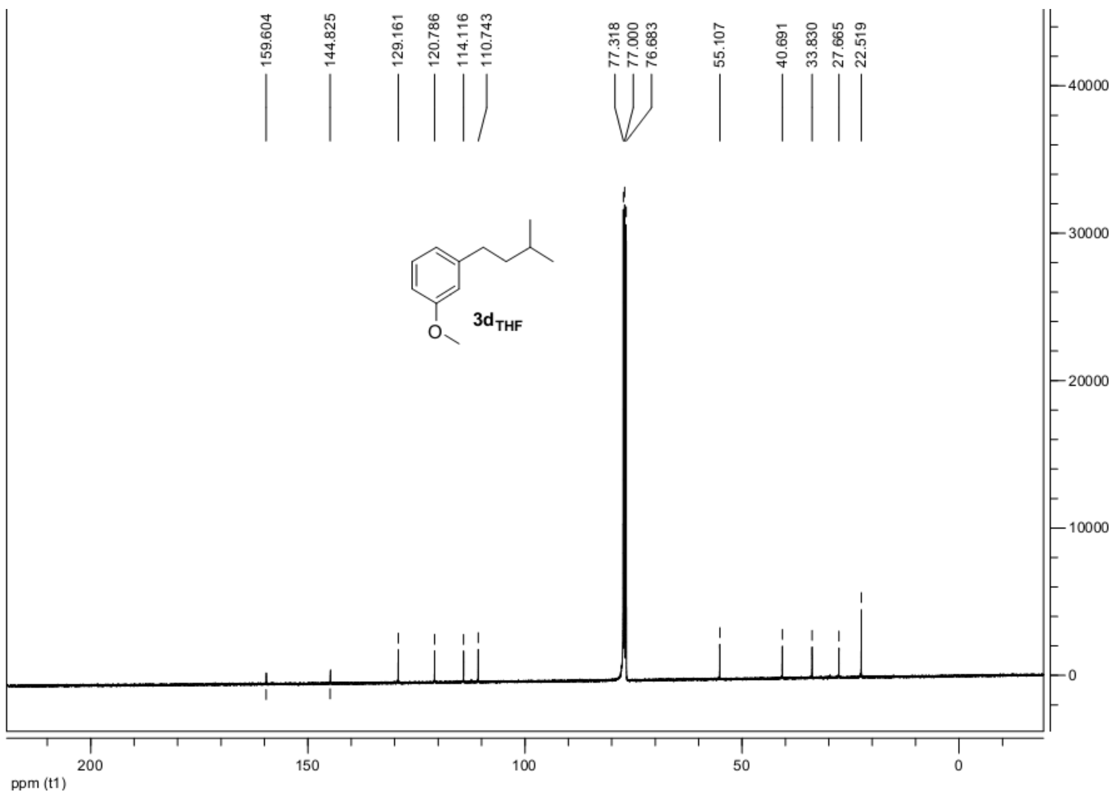

Supplementary Figure 37 | ^{13}C NMR spectrum of 1-isopentyl-3-methoxybenzene ($3d_{THF}$). (100 MHz, $CDCl_3$, 298 K).

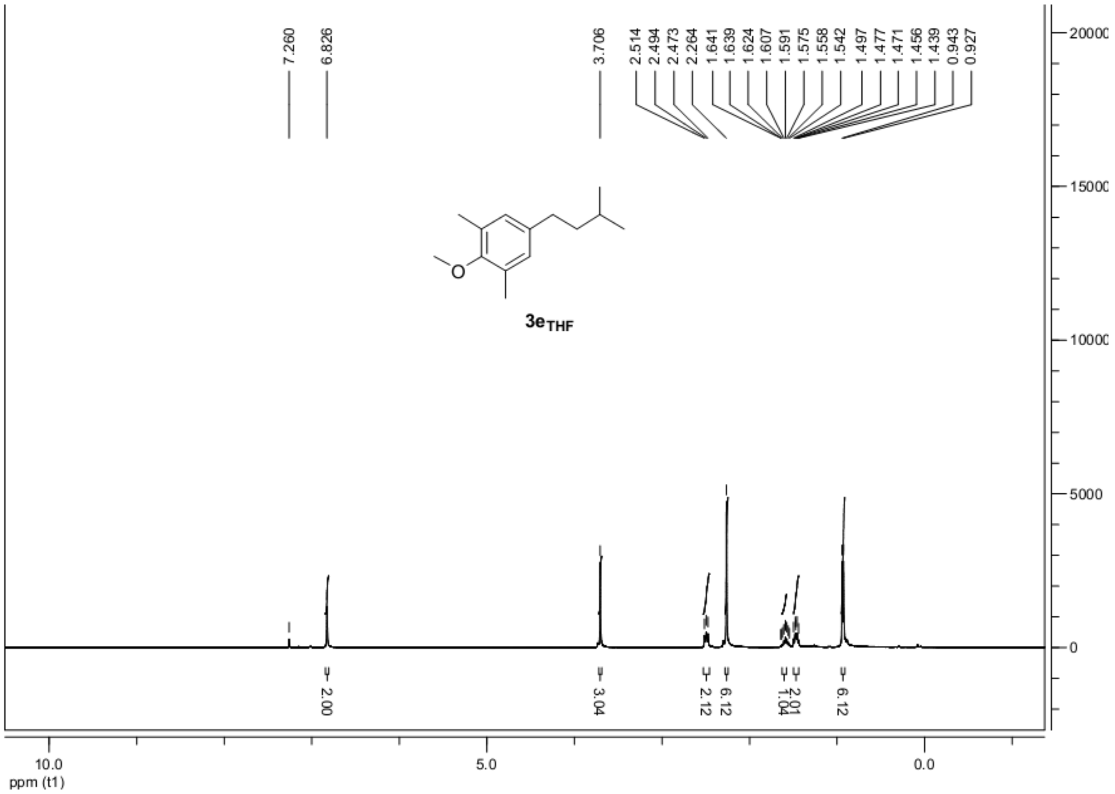

Supplementary Figure 38 | ^{1}H NMR spectrum of 5-isopentyl-2-methoxy-1,3-dimethylbenzene (3e$_{THF}$). (400 MHz, $CDCl_3$, 298 K).

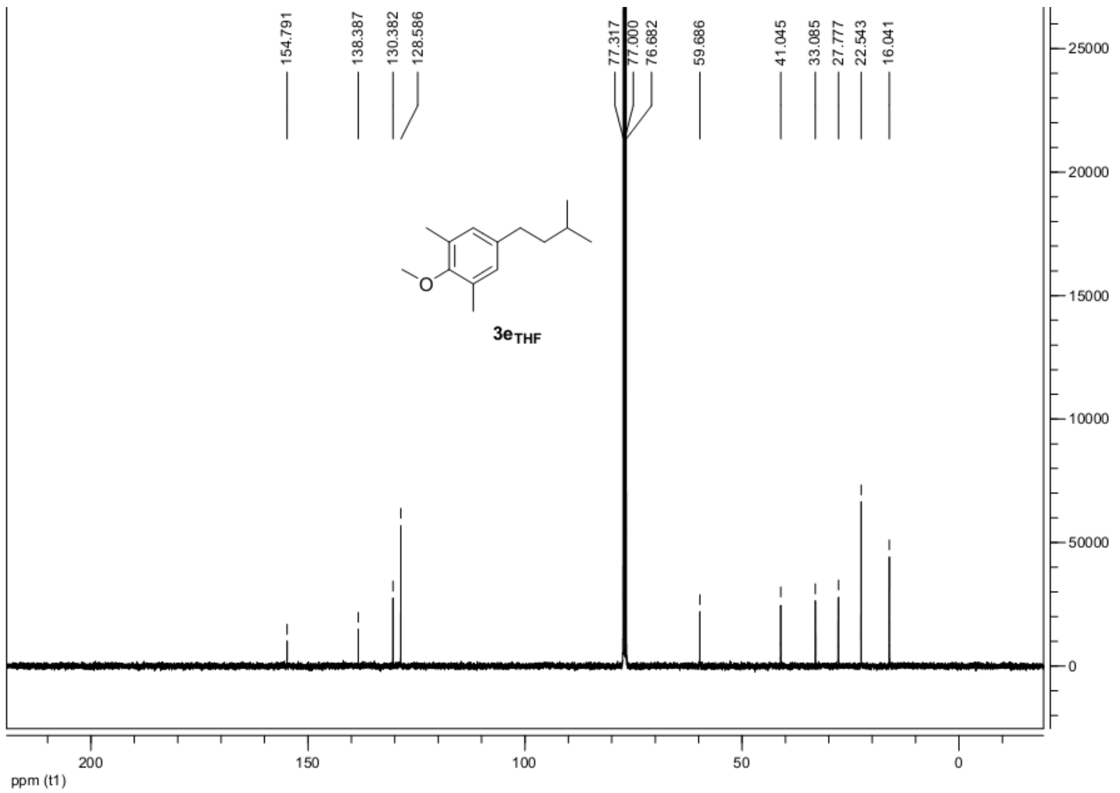

Supplementary Figure 39 | ^{13}C NMR spectrum of 5-isopentyl-2-methoxy-1,3-dimethylbenzene (3e$_{THF}$). (100 MHz, $CDCl_3$, 298 K).

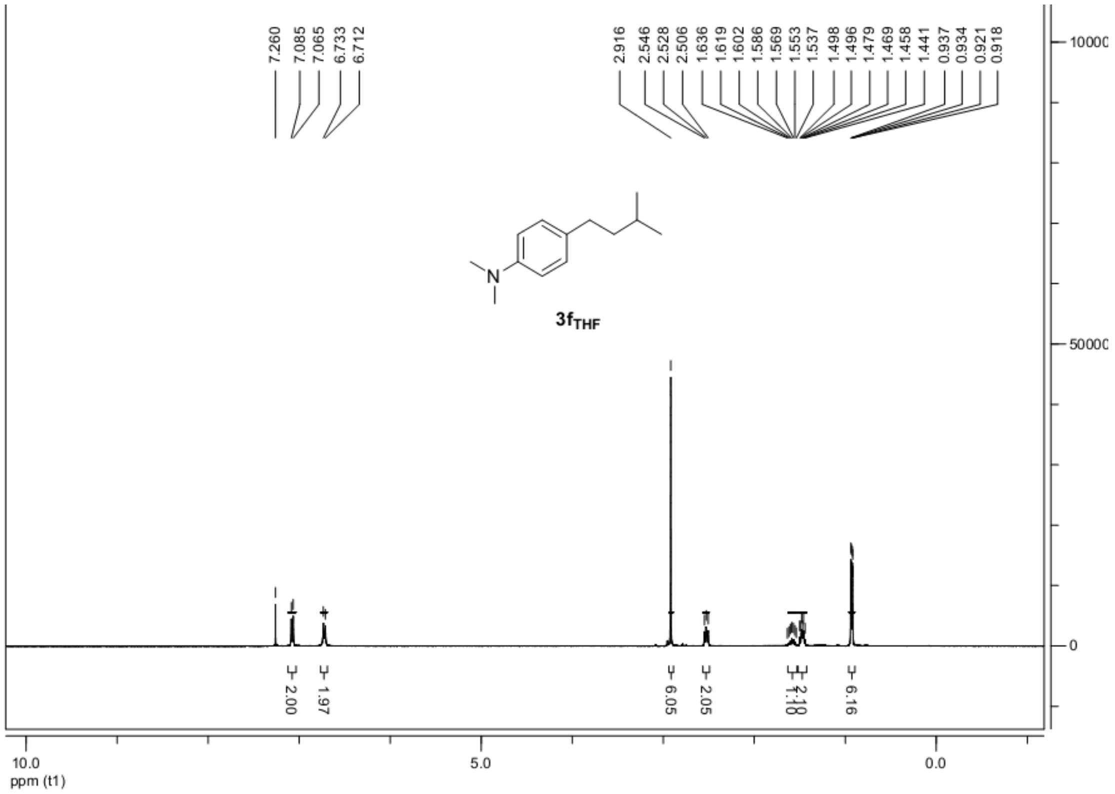

Supplementary Figure 40 | ^{1}H NMR spectrum of 4-isopentyl-*N*,*N*-dimethylaniline (3f$_{THF}$). (400 MHz, $CDCl_3$, 298 K).

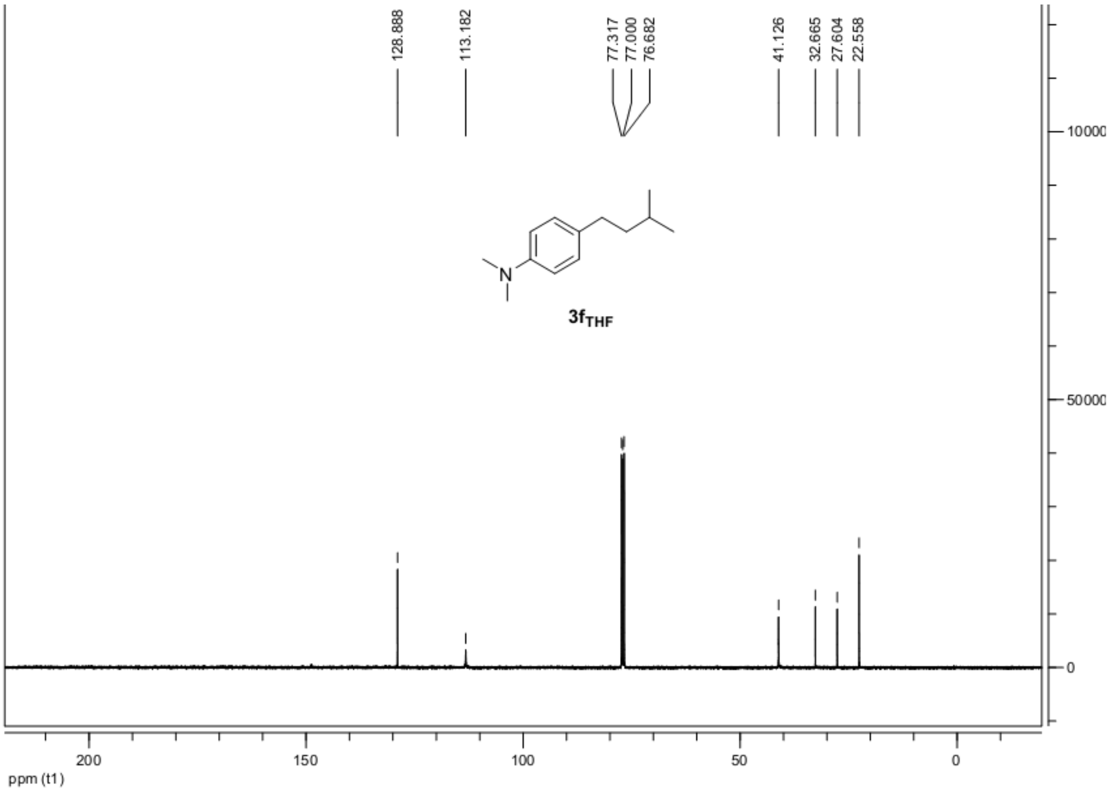

Supplementary Figure 41 | ^{13}C NMR spectrum of 4-isopentyl-*N*,*N*-dimethylaniline (3f$_{THF}$). (100 MHz, $CDCl_3$, 298 K).

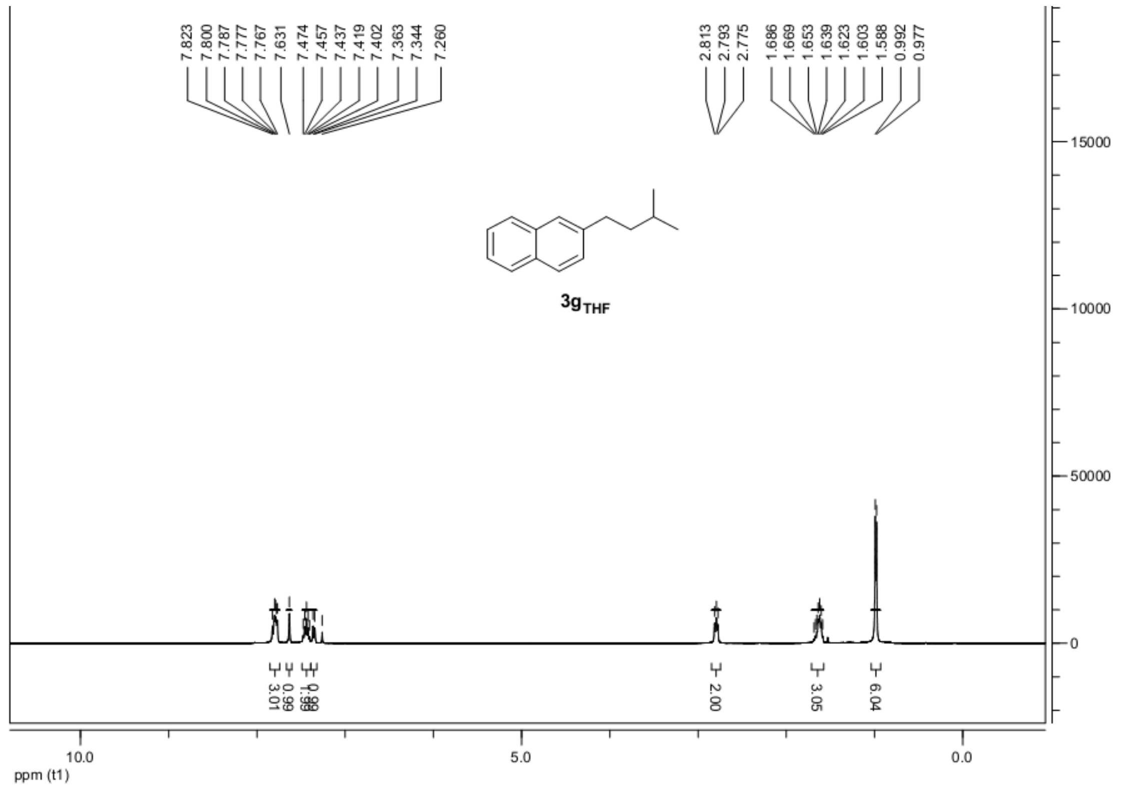

Supplementary Figure 42 | ^{1}H NMR spectrum of 2-isopentylnaphthalene ($3g_{THF}$). (400 MHz, $CDCl_3$, 298 K).

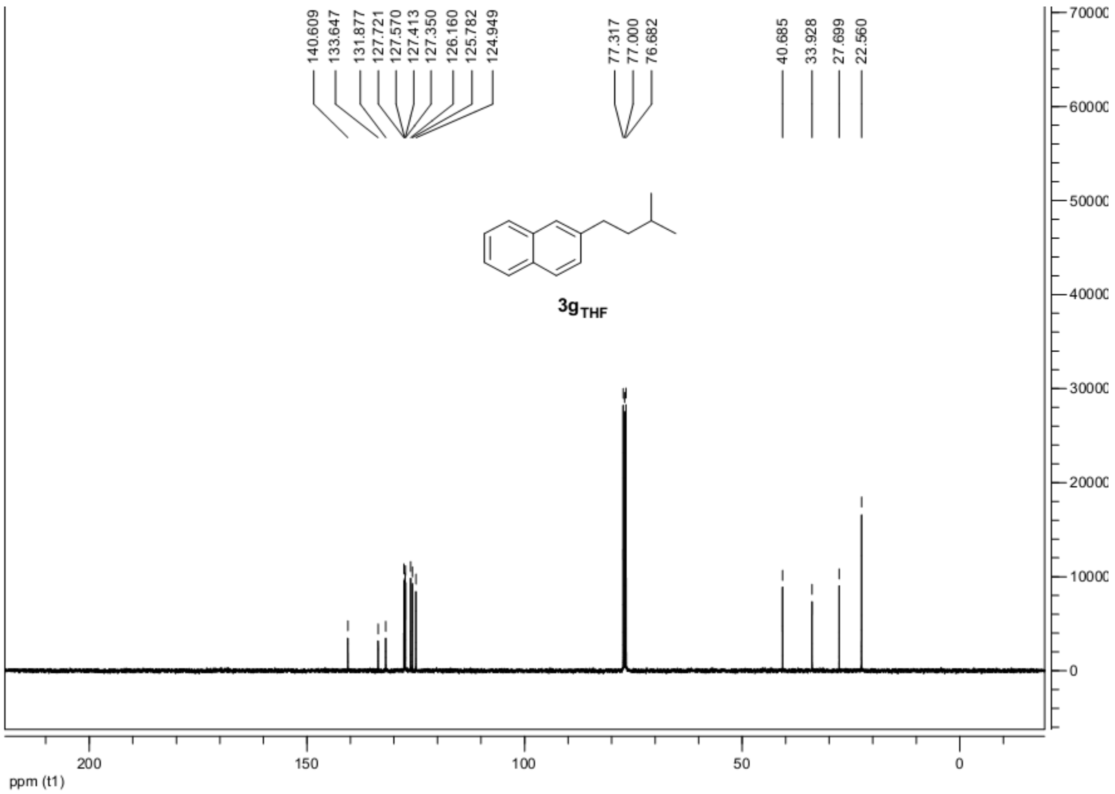

Supplementary Figure 43 | ^{13}C NMR spectrum of 2-isopentylnaphthalene (3g$_{THF}$). (100 MHz, $CDCl_3$, 298 K).

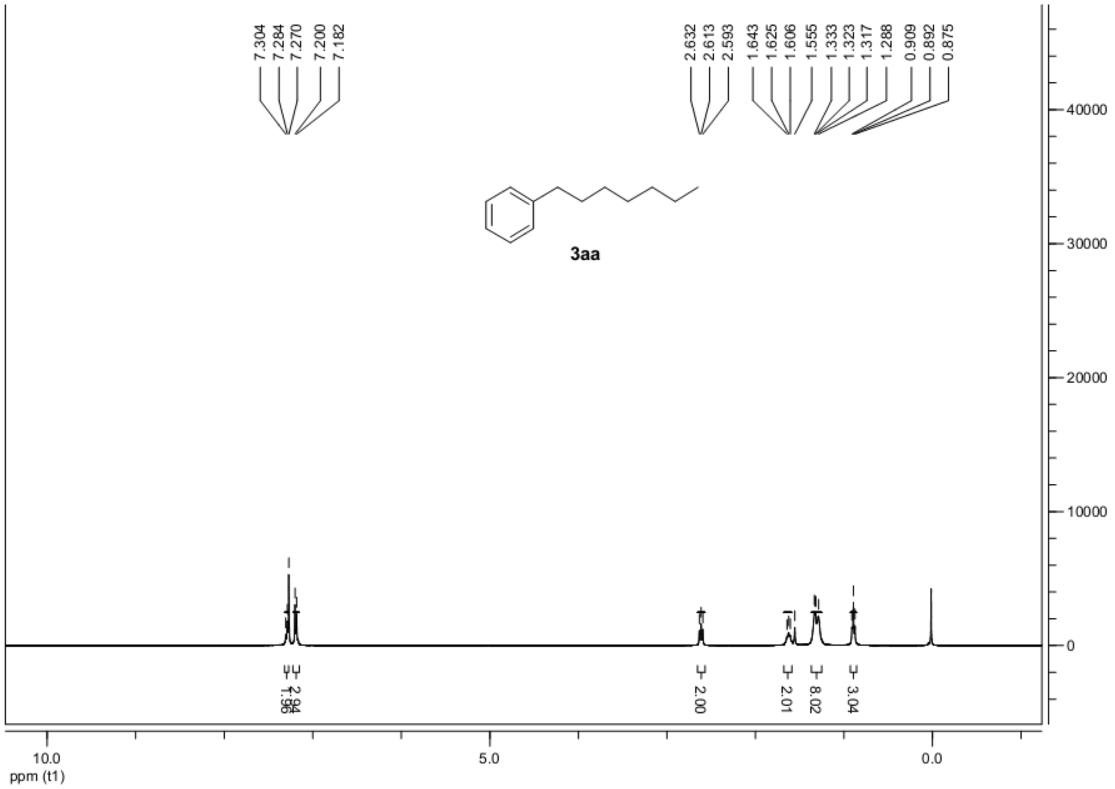

Supplementary Figure 44 | ^{1}H NMR spectrum of heptylbenzene (3aa). (400 MHz, $CDCl_3$, 298 K).

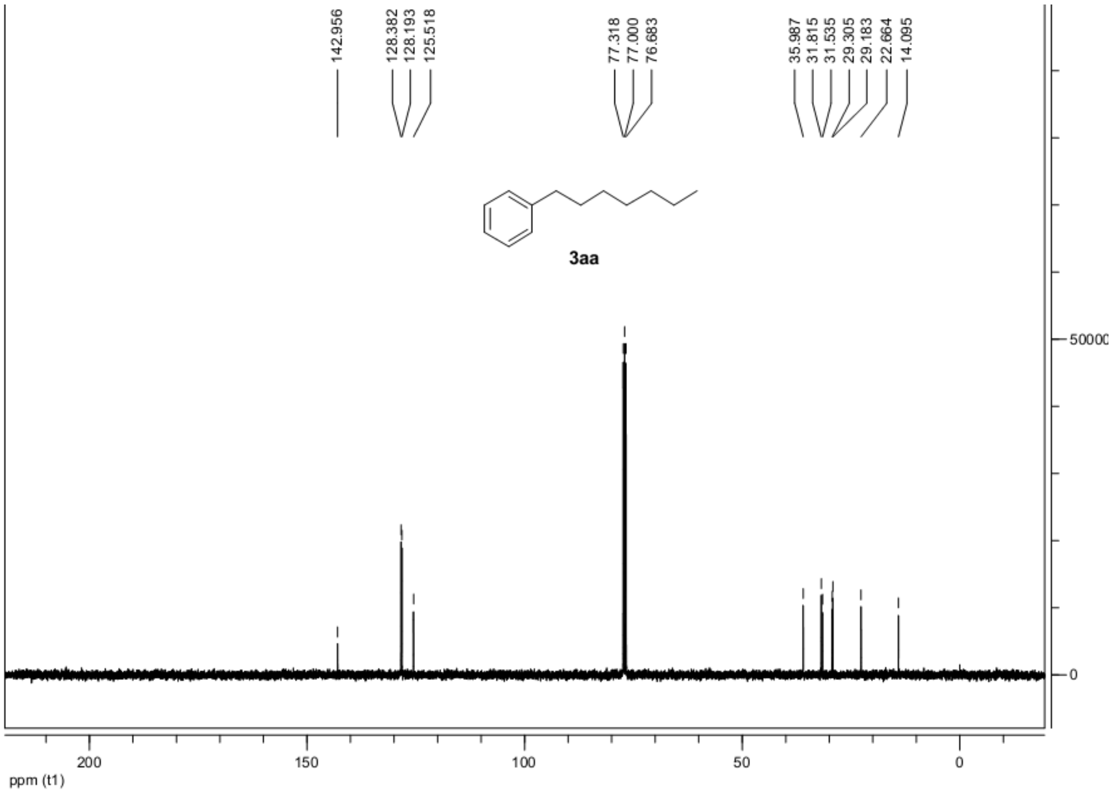

Supplementary Figure 45 | ^{13}C NMR spectrum of heptylbenzene (3aa). (100 MHz, $CDCl_3$, 298 K).

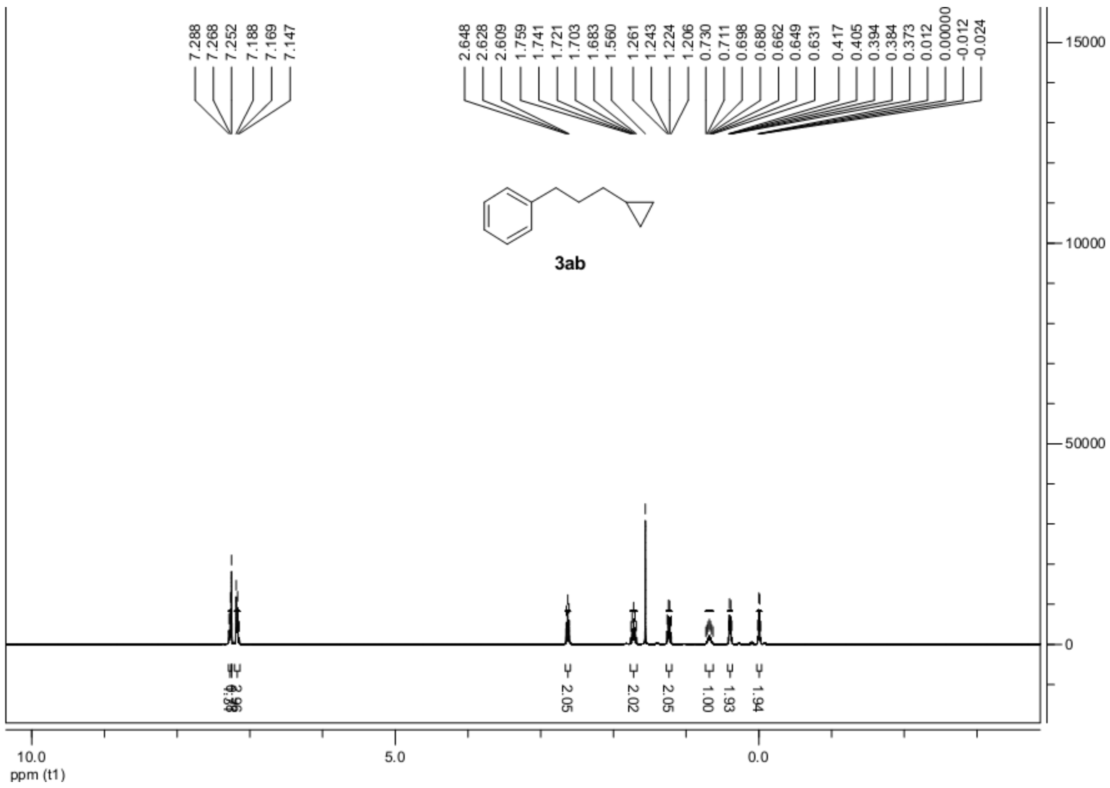

Supplementary Figure 46 | ^{1}H NMR spectrum of (3-cyclopropylpropyl)benzene (3ab). (400 MHz, $CDCl_3$, 298 K).

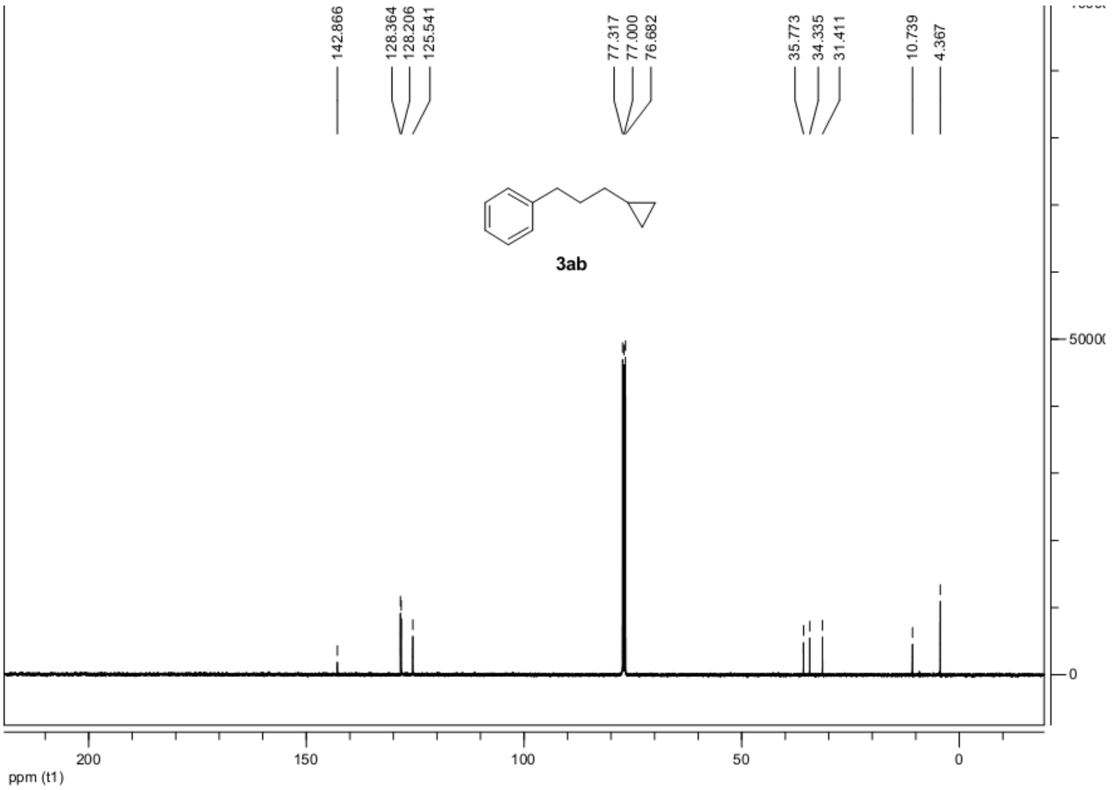

Supplementary Figure 47 | ^{13}C NMR spectrum of (3-cyclopropylpropyl)benzene (3ab). (100 MHz, $CDCl_3$, 298 K).

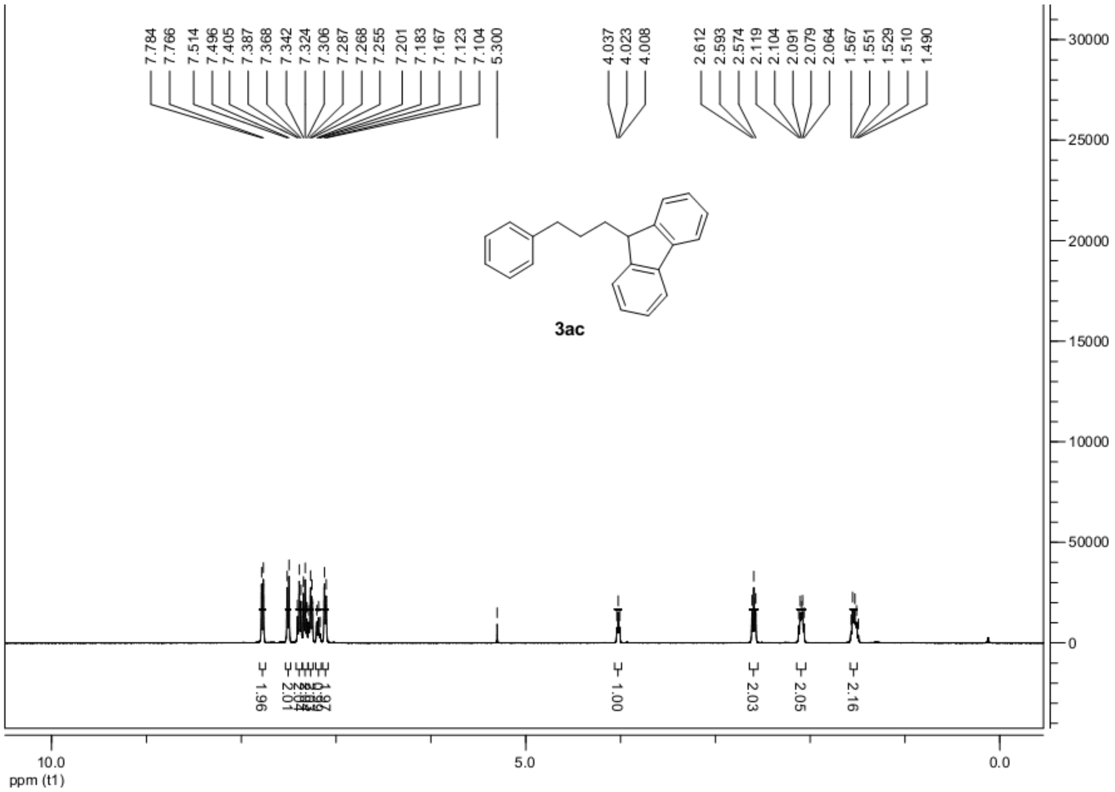

Supplementary Figure 48 | ^{1}H NMR spectrum of 9-(3-phenylpropyl)-9H-fluorene (3ac). (400 MHz, $CDCl_3$, 298 K).

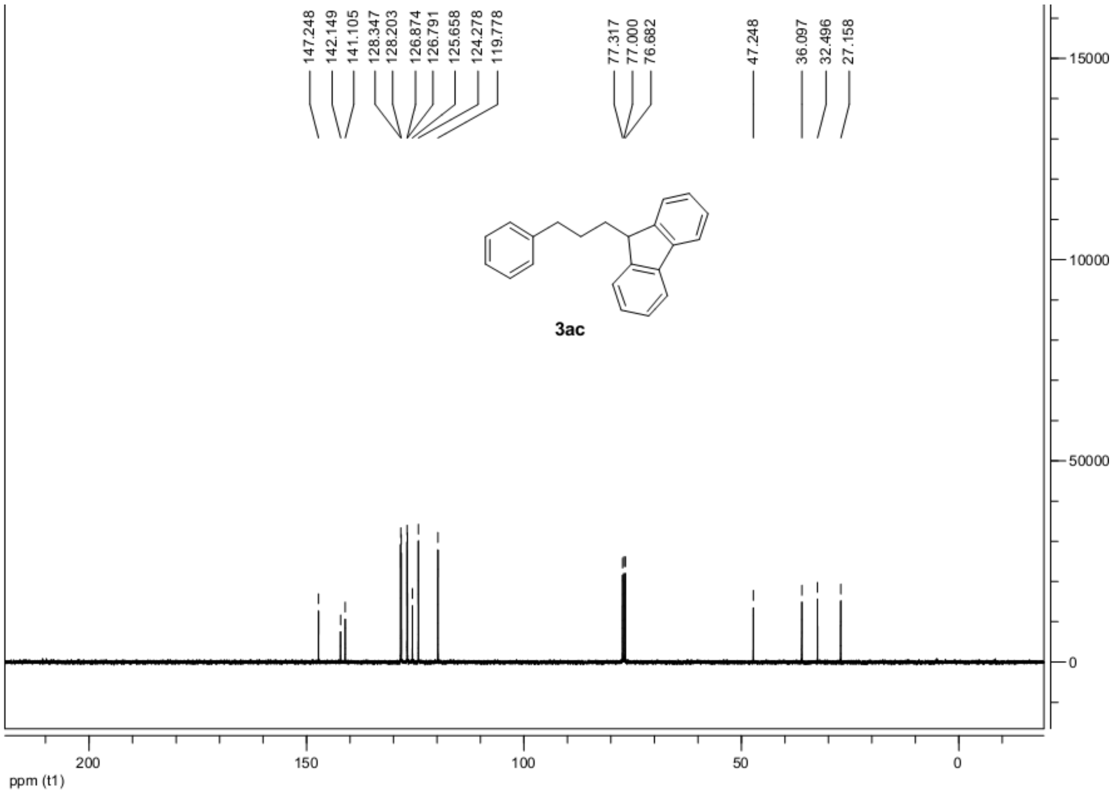

Supplementary Figure 49 | ^{13}C NMR spectrum of 9-(3-phenylpropyl)-9H-fluorene (3ac). (100 MHz, $CDCl_3$, 298 K).

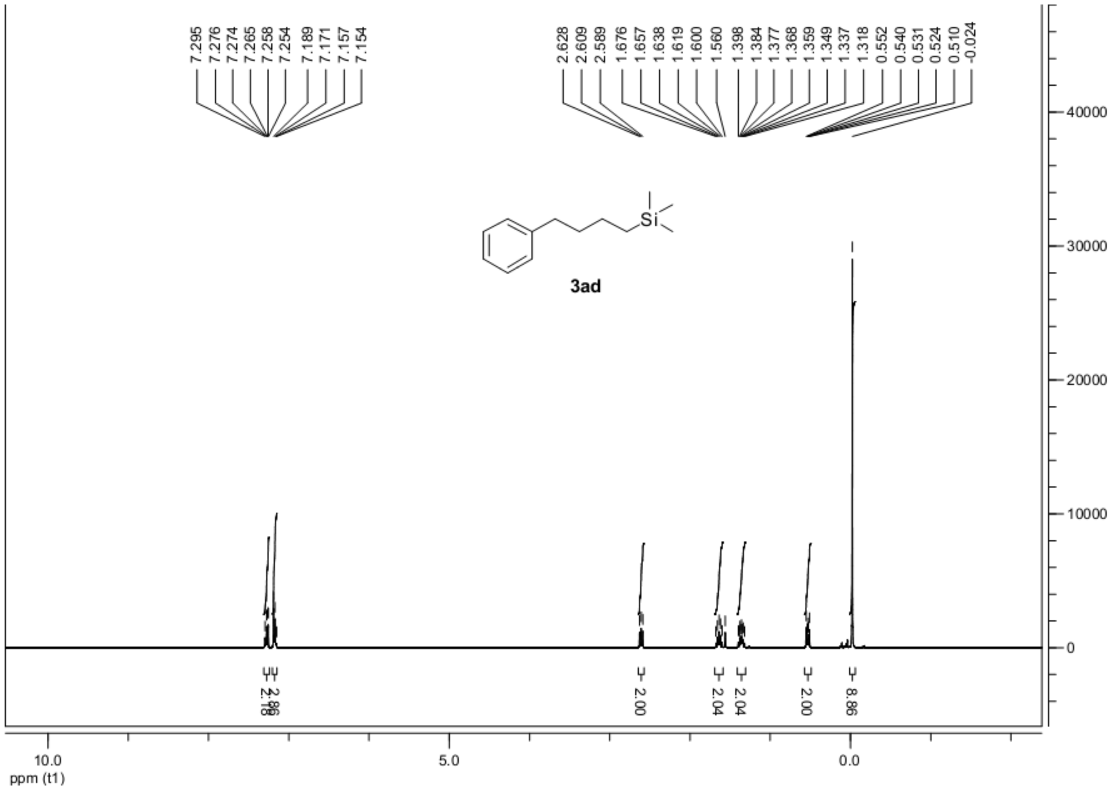

Supplementary Figure 50 | ^{1}H NMR spectrum of trimethyl(4-phenylbutyl)silane (3ad). (400 MHz, $CDCl_3$, 298 K).

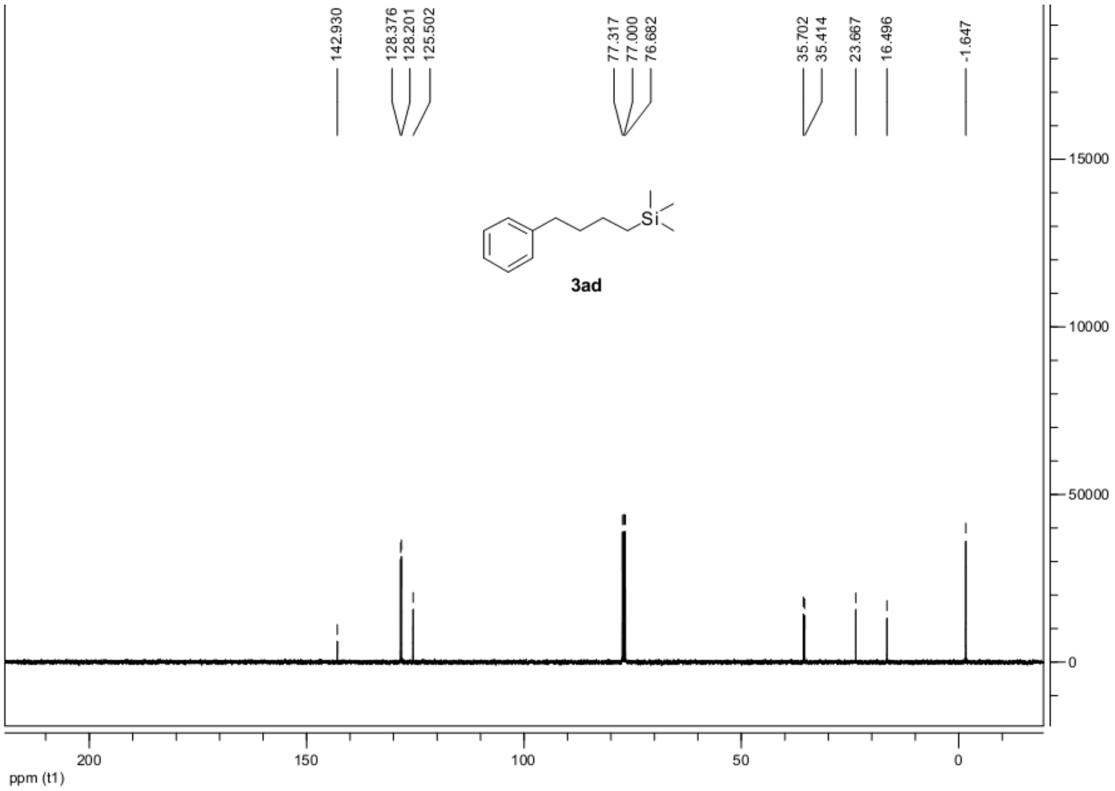

Supplementary Figure 51 | ^{13}C NMR spectrum of trimethyl(4-phenylbutyl)silane (3ad). (100 MHz, $CDCl_3$, 298 K).

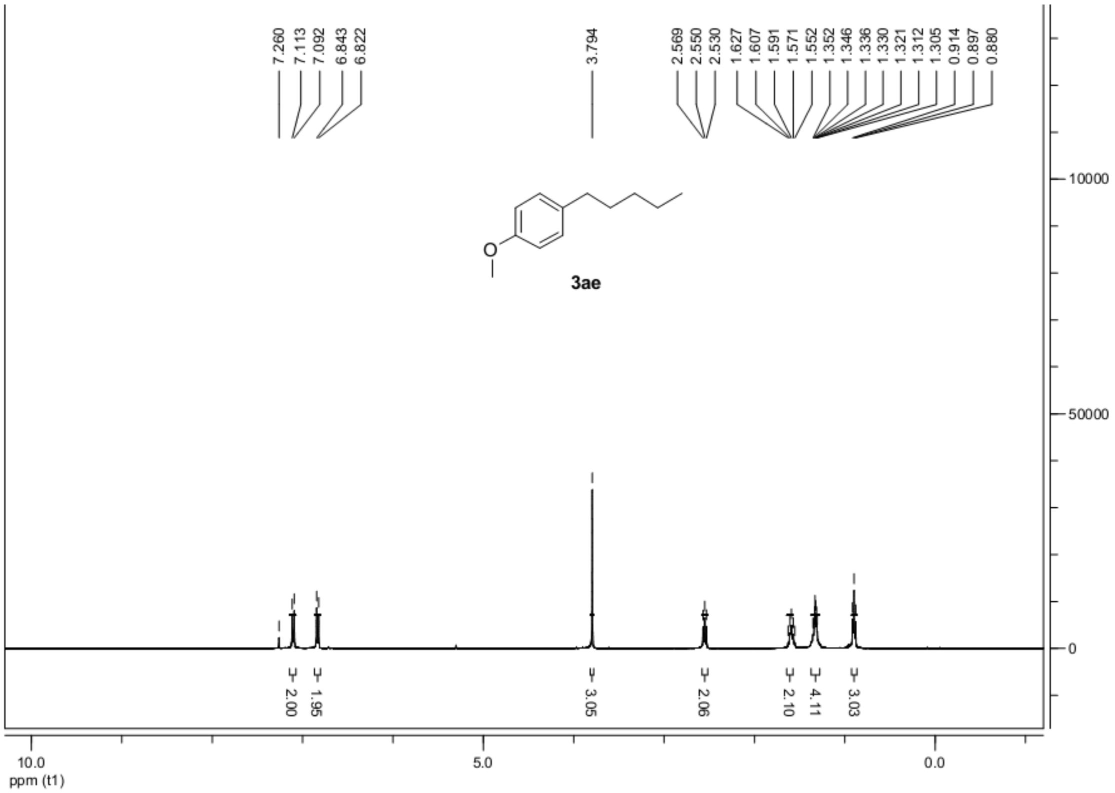

Supplementary Figure 52 | ^{1}H NMR spectrum of 1-methoxy-4-pentylbenzene (3ae). (400 MHz, $CDCl_3$, 298 K).

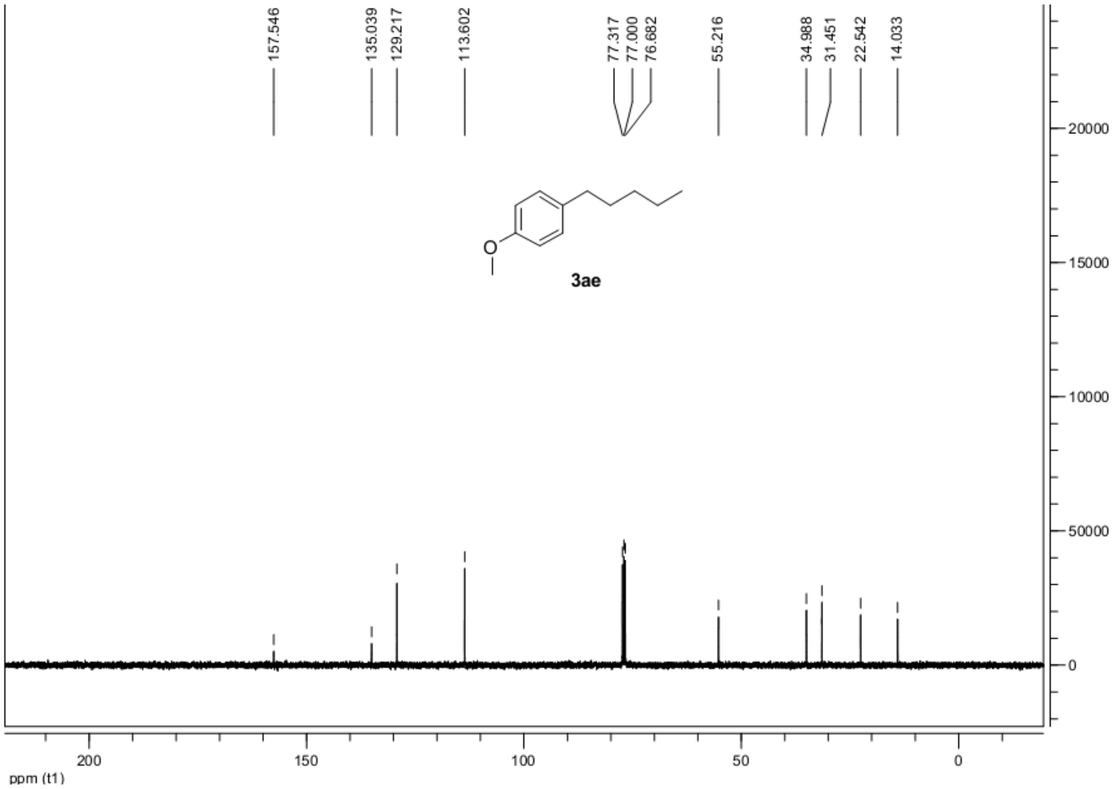

Supplementary Figure 53 | ^{13}C NMR spectrum of 1-methoxy-4-pentylbenzene (3ae). (100 MHz, $CDCl_3$, 298 K).

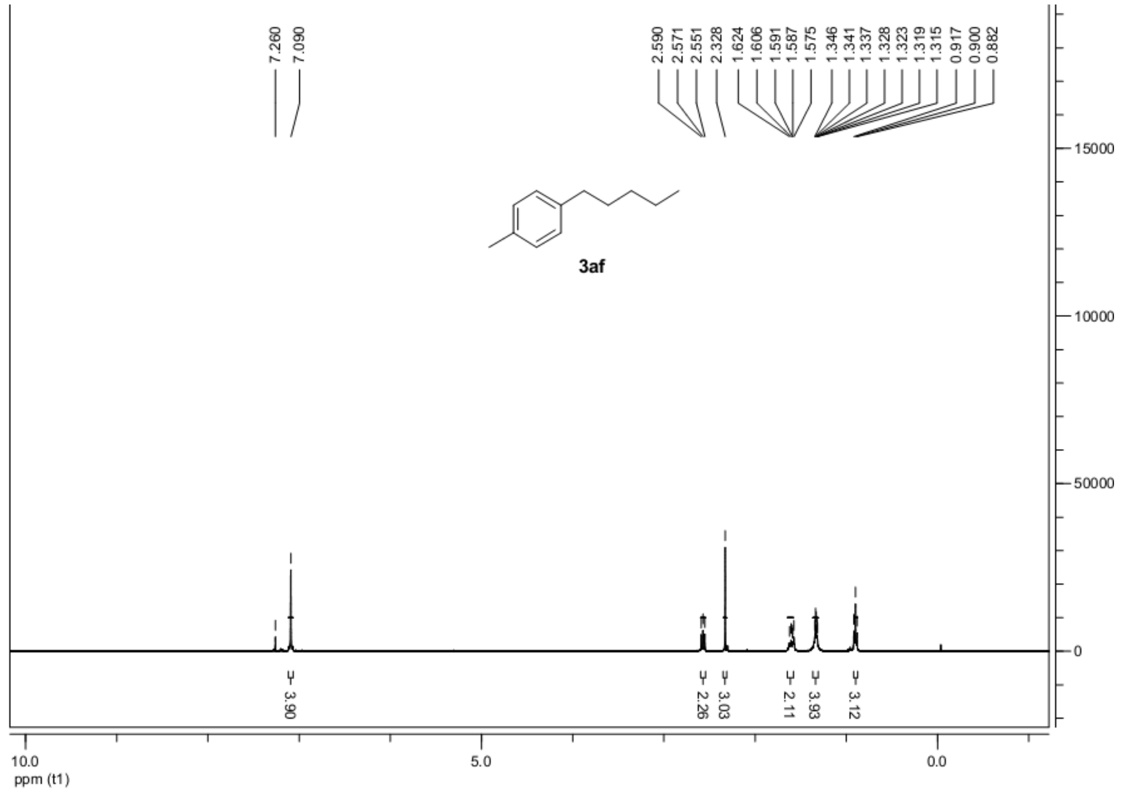

Supplementary Figure 54 | ^{1}H NMR spectrum of 1-methyl-4-pentylbenzene (3af). (400 MHz, $CDCl_3$, 298 K).

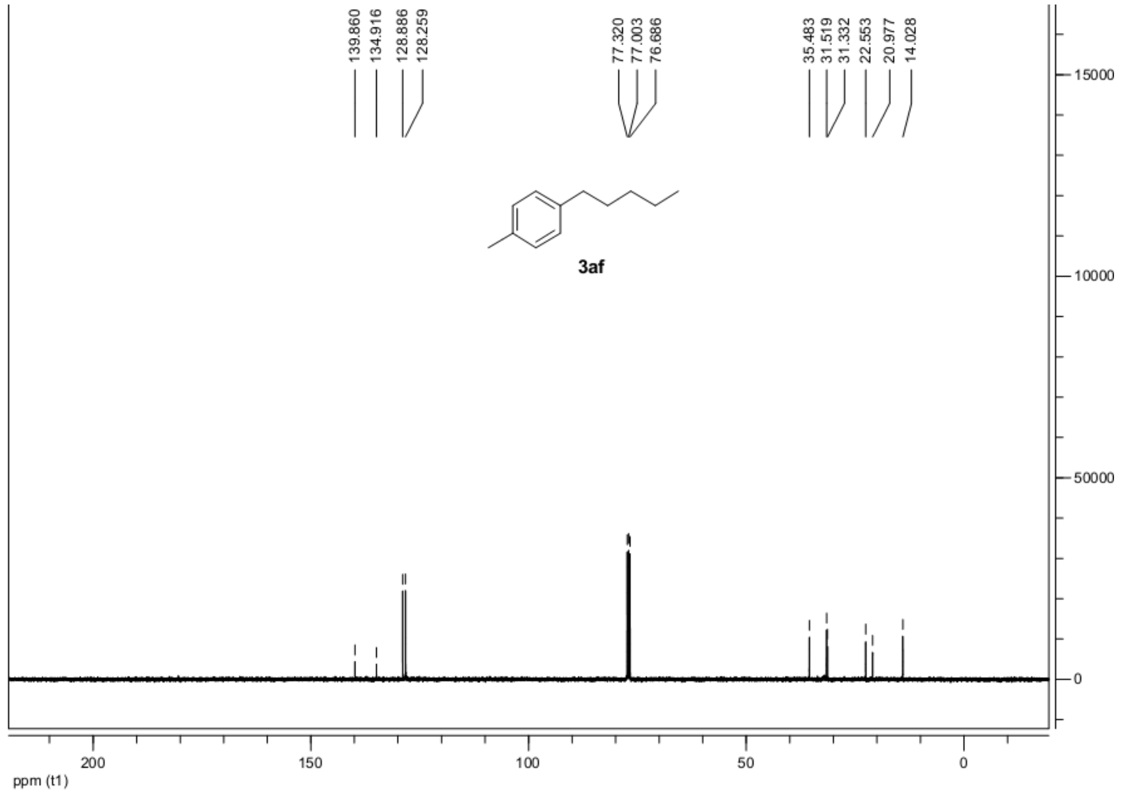

Supplementary Figure 55 | ^{13}C NMR spectrum of 1-methyl-4-pentylbenzene (3af). (100 MHz, $CDCl_3$, 298 K).

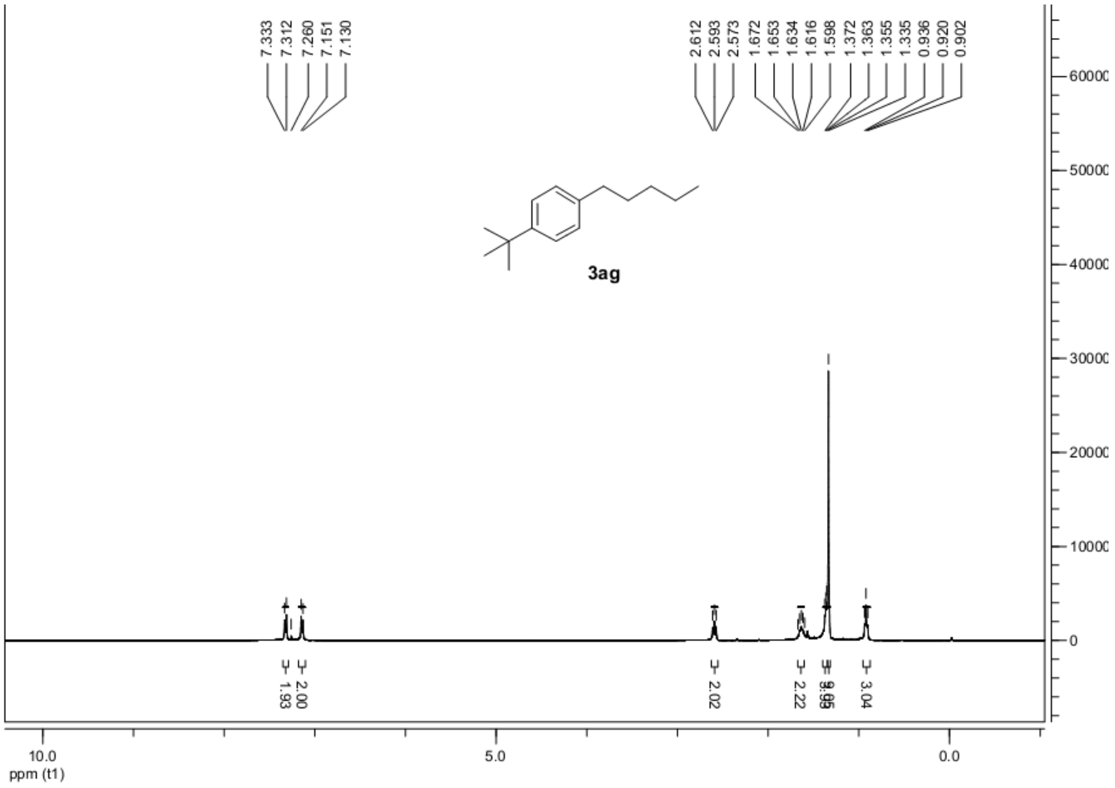

Supplementary Figure 56 | ^{1}H NMR spectrum of 1-*tert*-Butyl-4-pentylbenzene (3ag). (400 MHz, $CDCl_3$, 298 K).

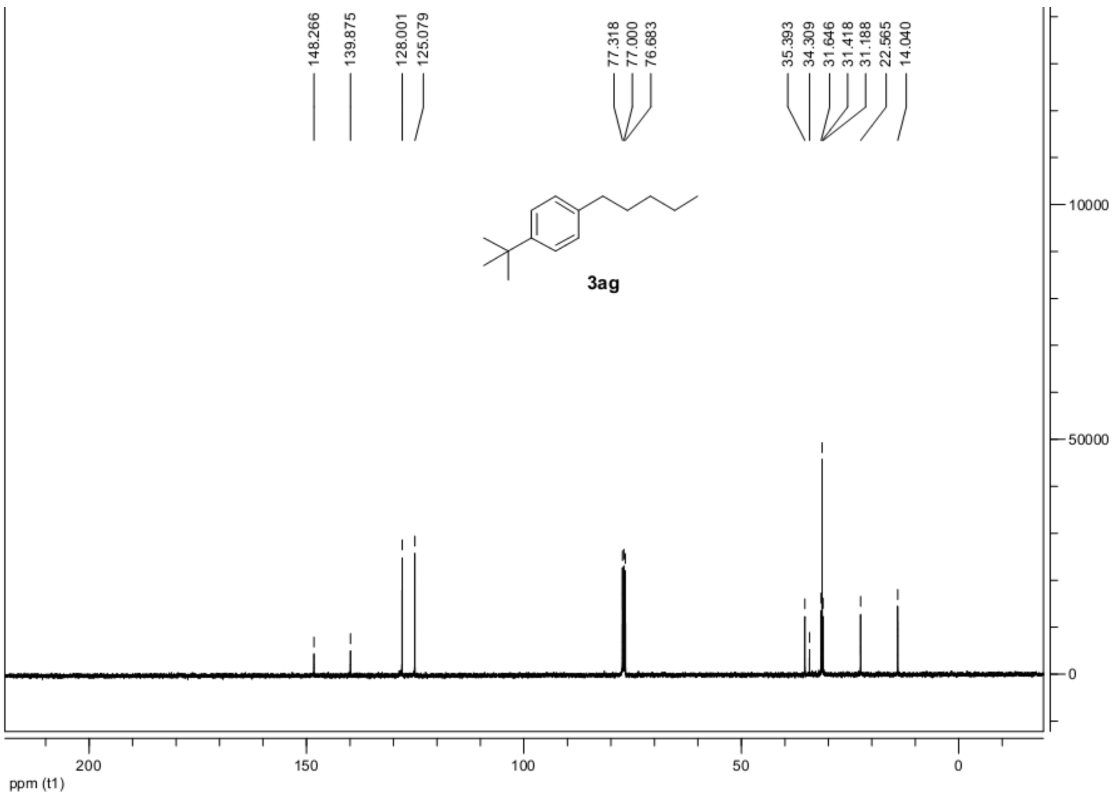

Supplementary Figure 57 | ^{13}C NMR spectrum of 1-*tert*-butyl-4-pentylbenzene (3ag). (100 MHz, $CDCl_3$, 298 K).

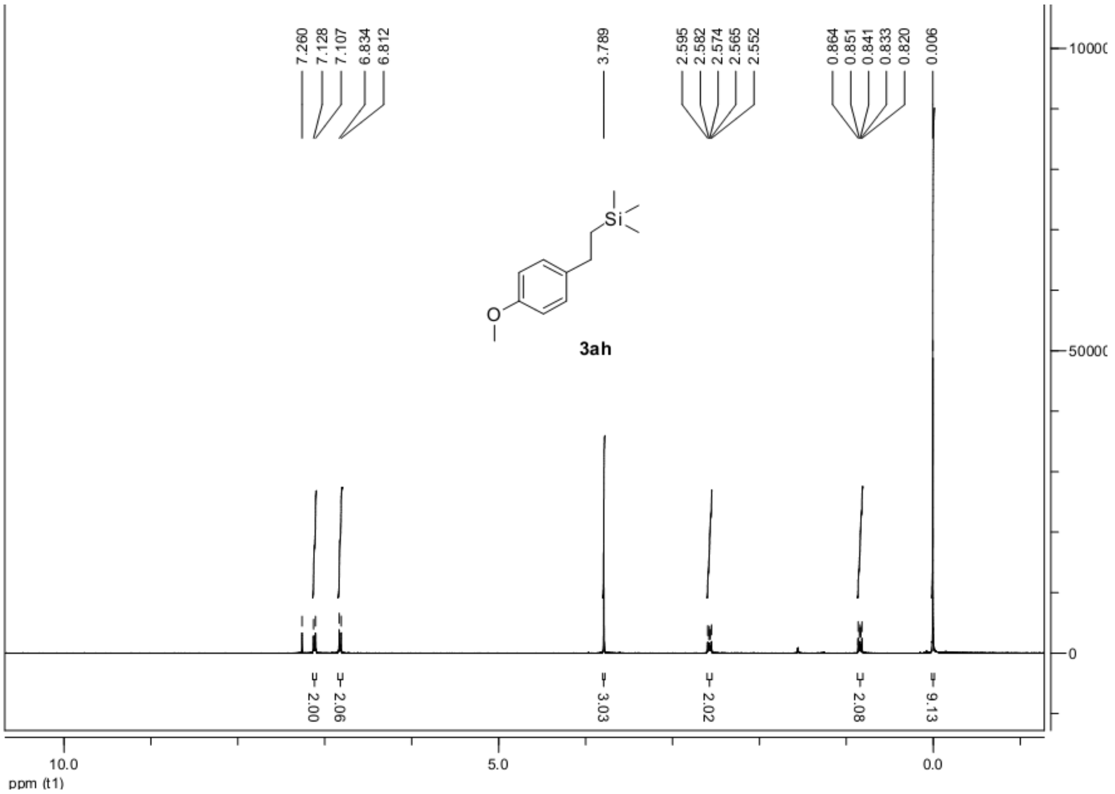

Supplementary Figure 58 | ^{1}H NMR spectrum of (4-methoxyphenethyl)trimethylsilane (3ah). (400 MHz, $CDCl_3$, 298 K).

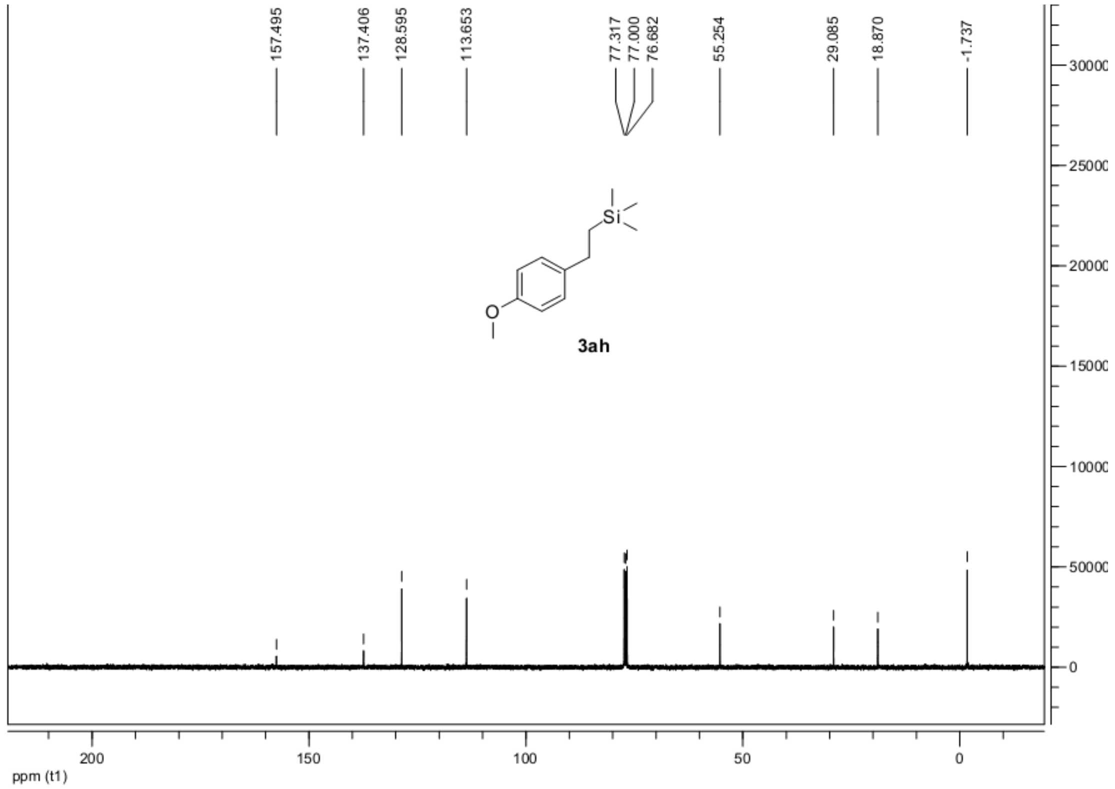

Supplementary Figure 59 | ^{13}C NMR spectrum of (4-methoxyphenethyl)trimethylsilane (3ah). (100 MHz, $CDCl_3$, 298 K).

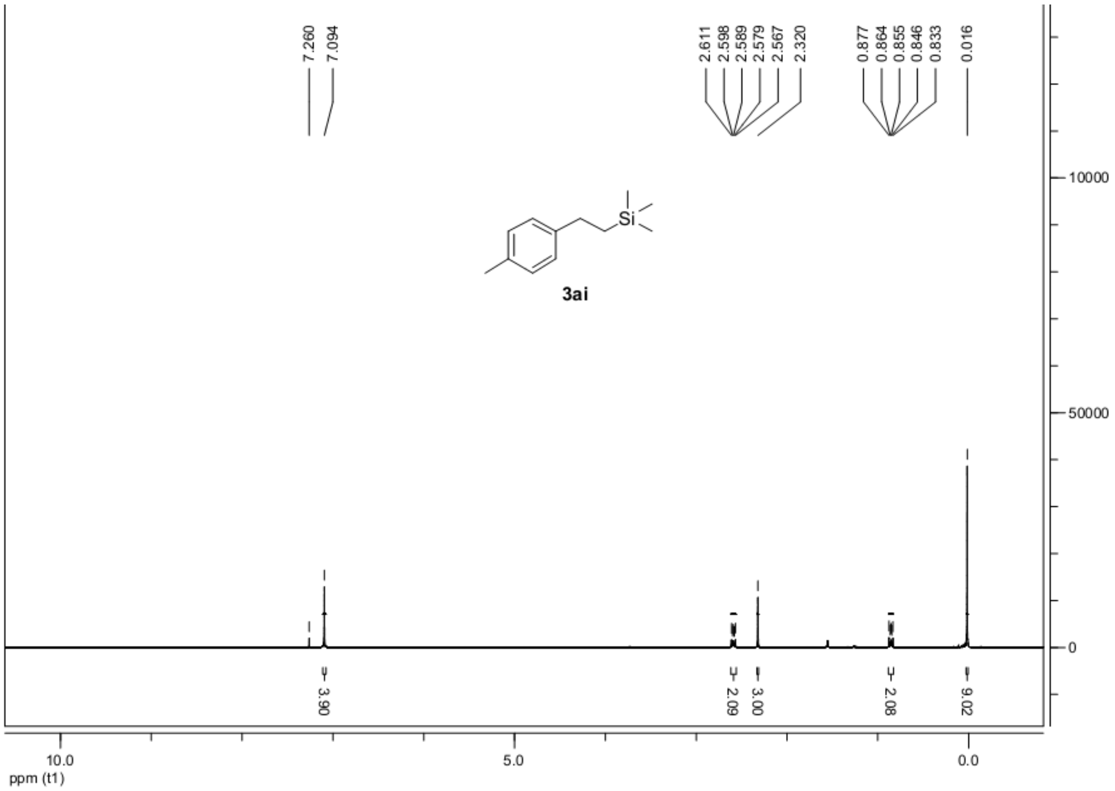

Supplementary Figure 60 | ^{1}H NMR spectrum of trimethyl(4-methylphenethyl)silane (3ai). (400 MHz, $CDCl_3$, 298 K).

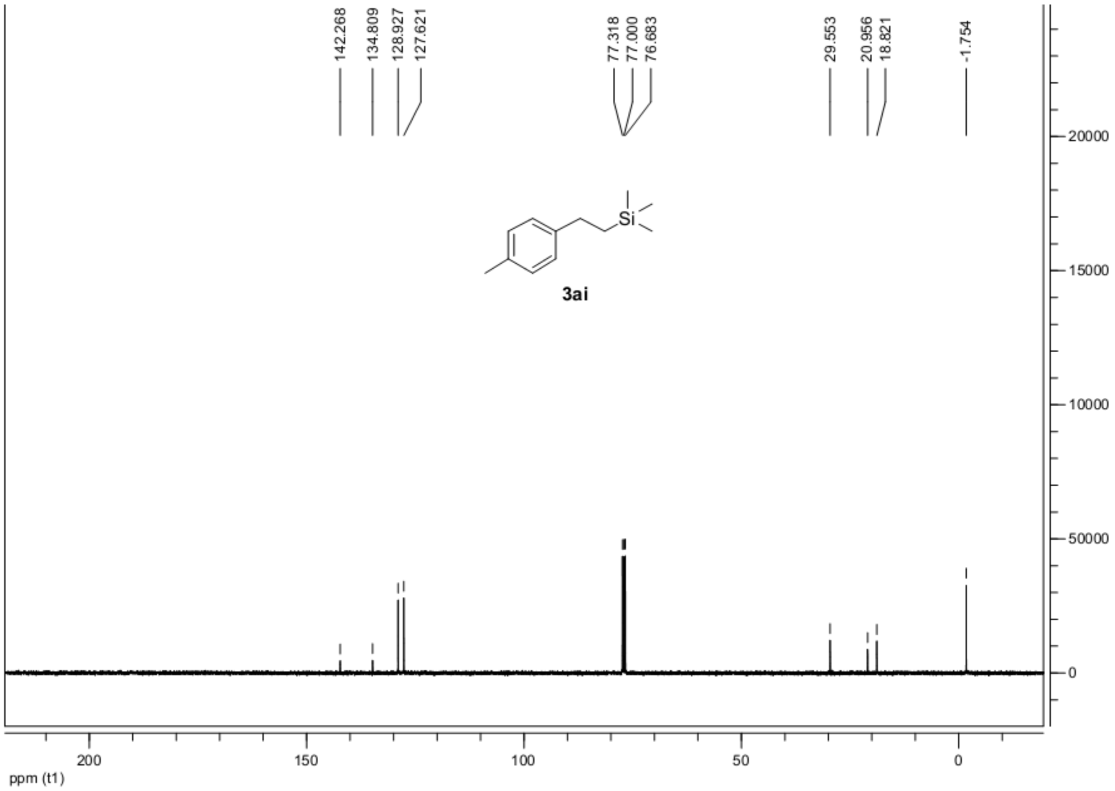

Supplementary Figure 61 | ^{13}C NMR spectrum of trimethyl(4-methylphenethyl)silane (3ai). (100 MHz, $CDCl_3$, 298 K).

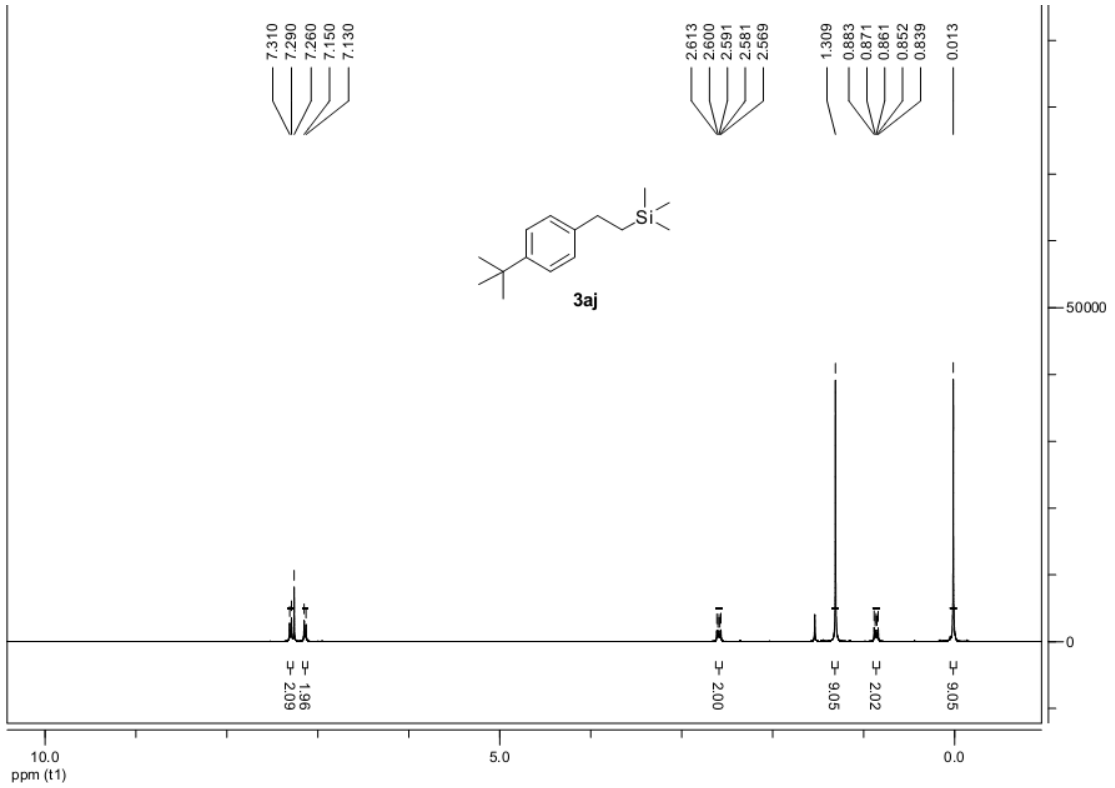

Supplementary Figure 62 | ^{1}H NMR spectrum of (4-*tert*-butylphenethyl)trimethylsilane (3aj). (400 MHz, $CDCl_3$, 298 K).

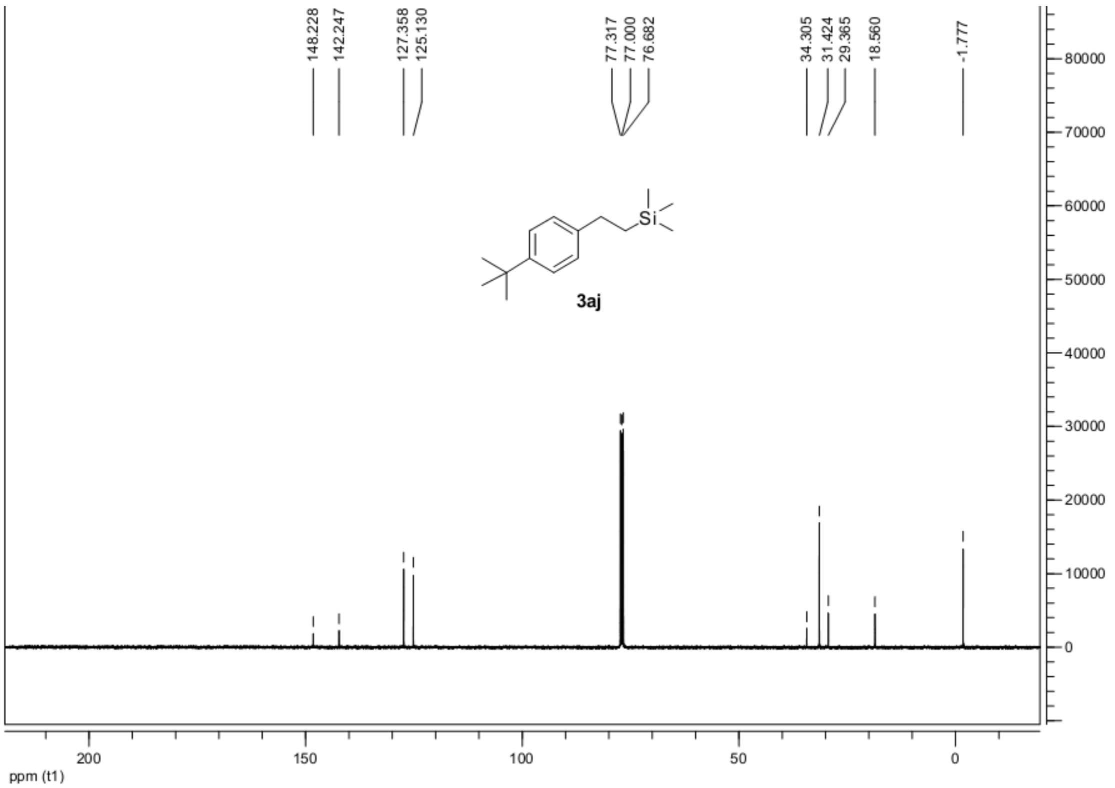

Supplementary Figure 63 | ^{13}C NMR spectrum of (4-*tert*-butylphenethyl)trimethylsilane (3aj). (100 MHz, $CDCl_3$, 298 K).

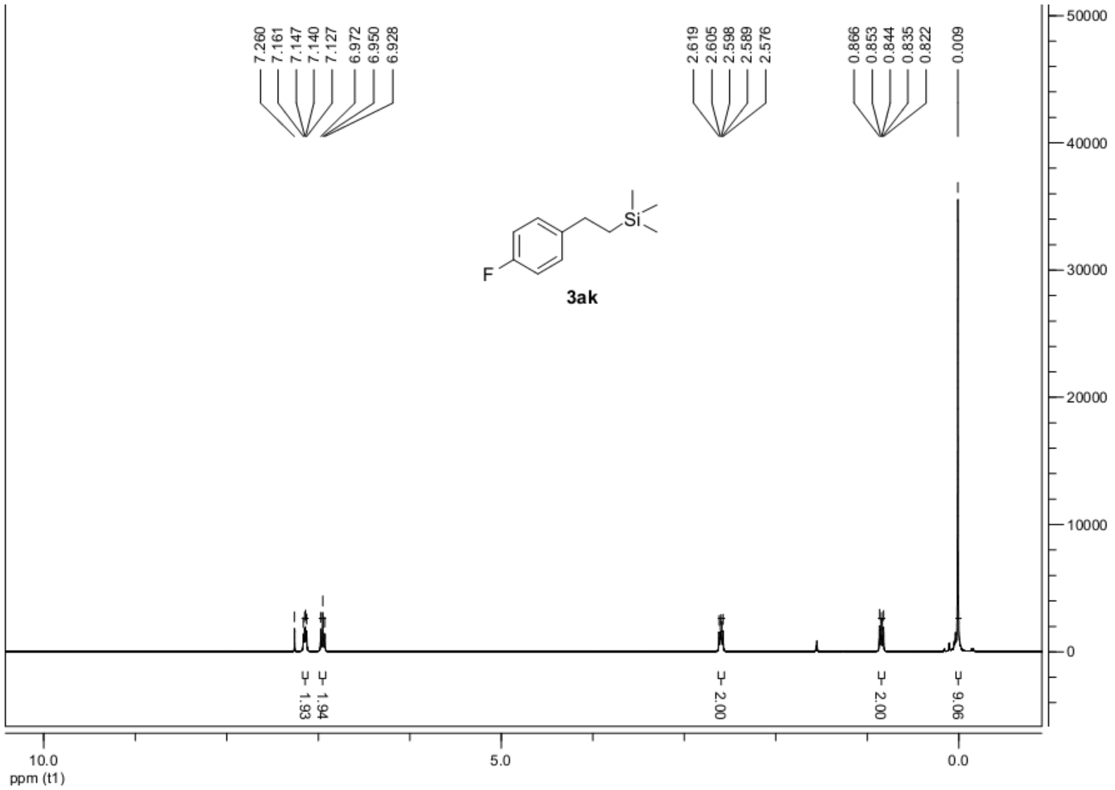

Supplementary Figure 64 | ^{1}H NMR spectrum of (4-fluorophenethyl)trimethylsilane (3ak). (400 MHz, $CDCl_3$, 298 K).

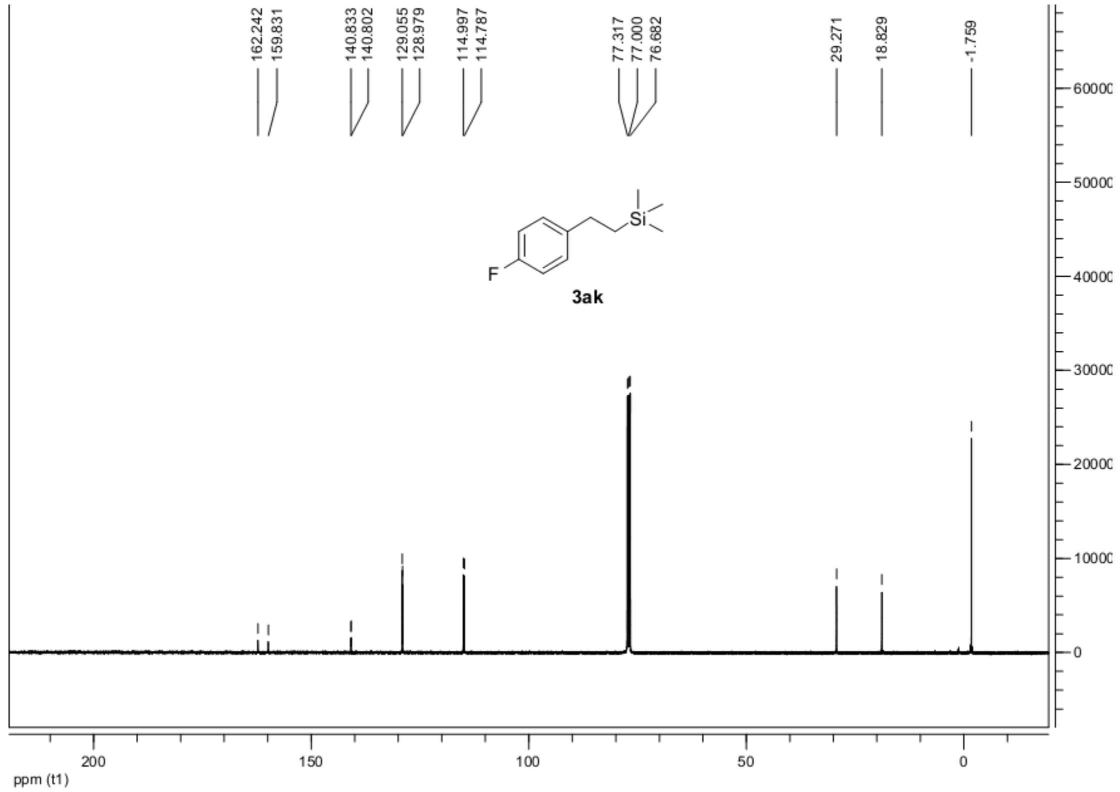

Supplementary Figure 65 | ^{13}C NMR spectrum of (4-fluorophenethyl)trimethylsilane (3ak). (100 MHz, $CDCl_3$, 298 K).

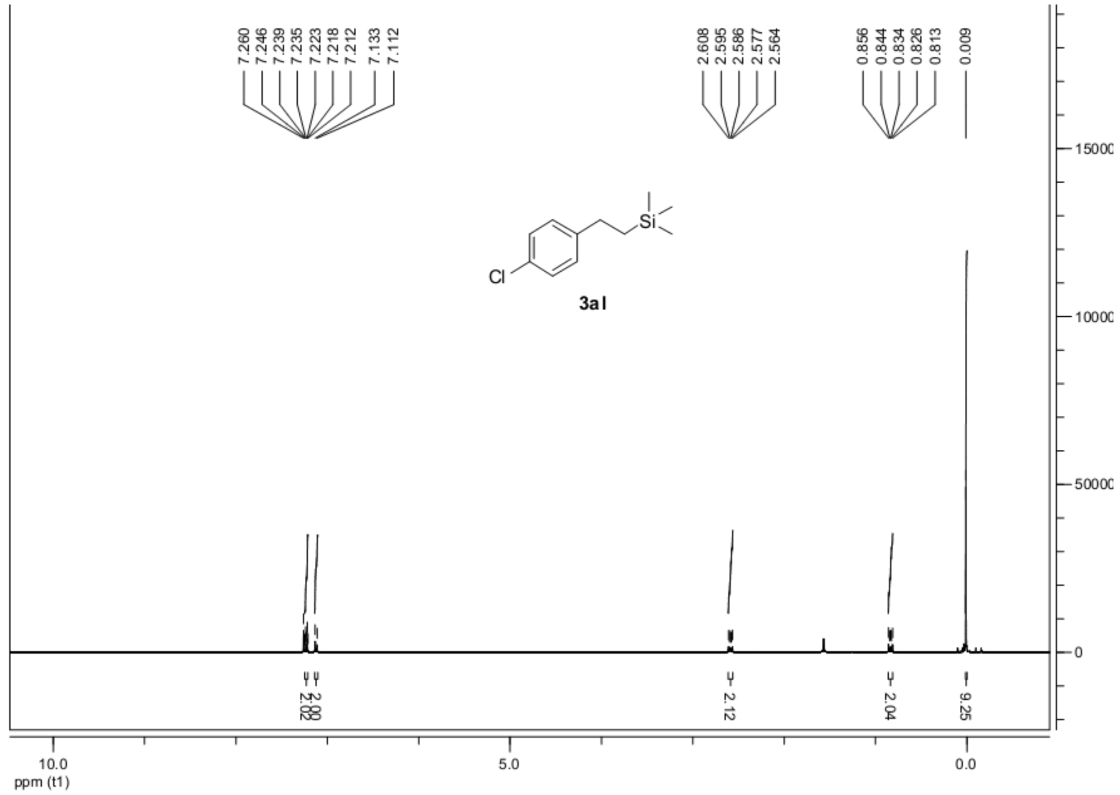

Supplementary Figure 66 | ^{1}H NMR spectrum of (4-chlorophenethyl)trimethylsilane (3al). (400 MHz, $CDCl_3$, 298 K).

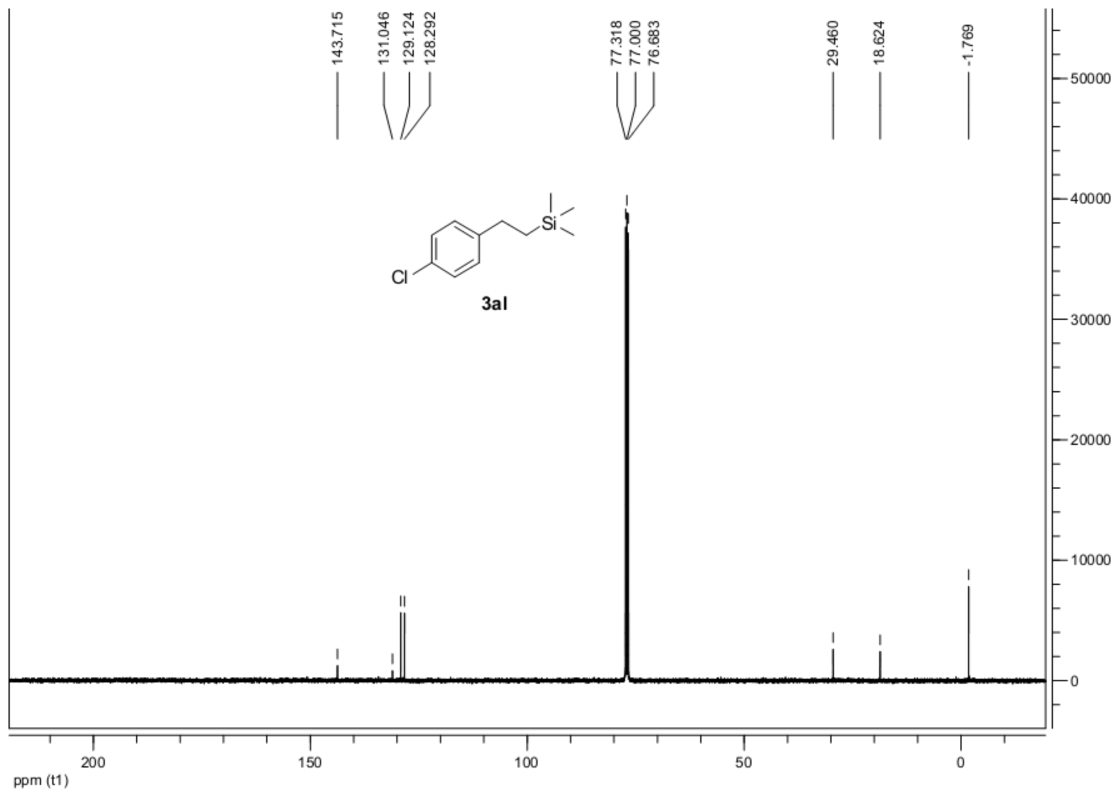

Supplementary Figure 67 | ^{13}C NMR spectrum of (4-chlorophenethyl)trimethylsilane (3al). (100 MHz, $CDCl_3$, 298 K).

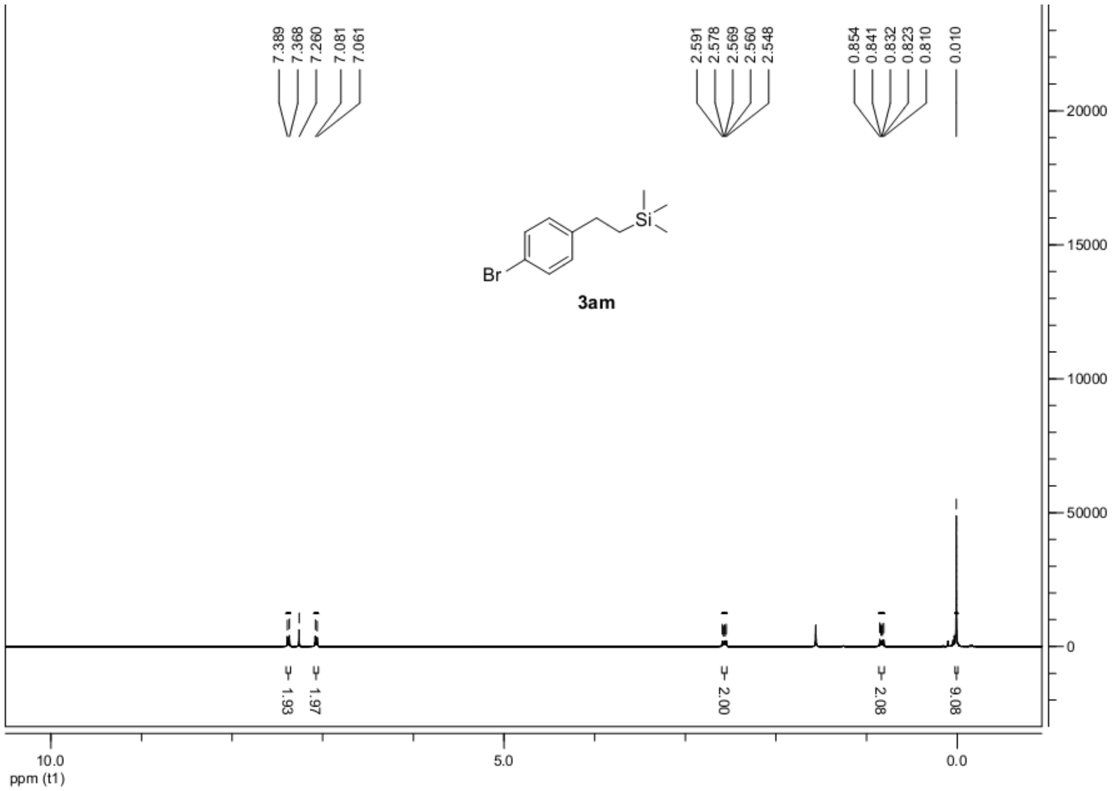

Supplementary Figure 68 | ^{1}H NMR spectrum of (4-bromophenethyl)trimethylsilane (3am). (400 MHz, $CDCl_3$, 298 K).

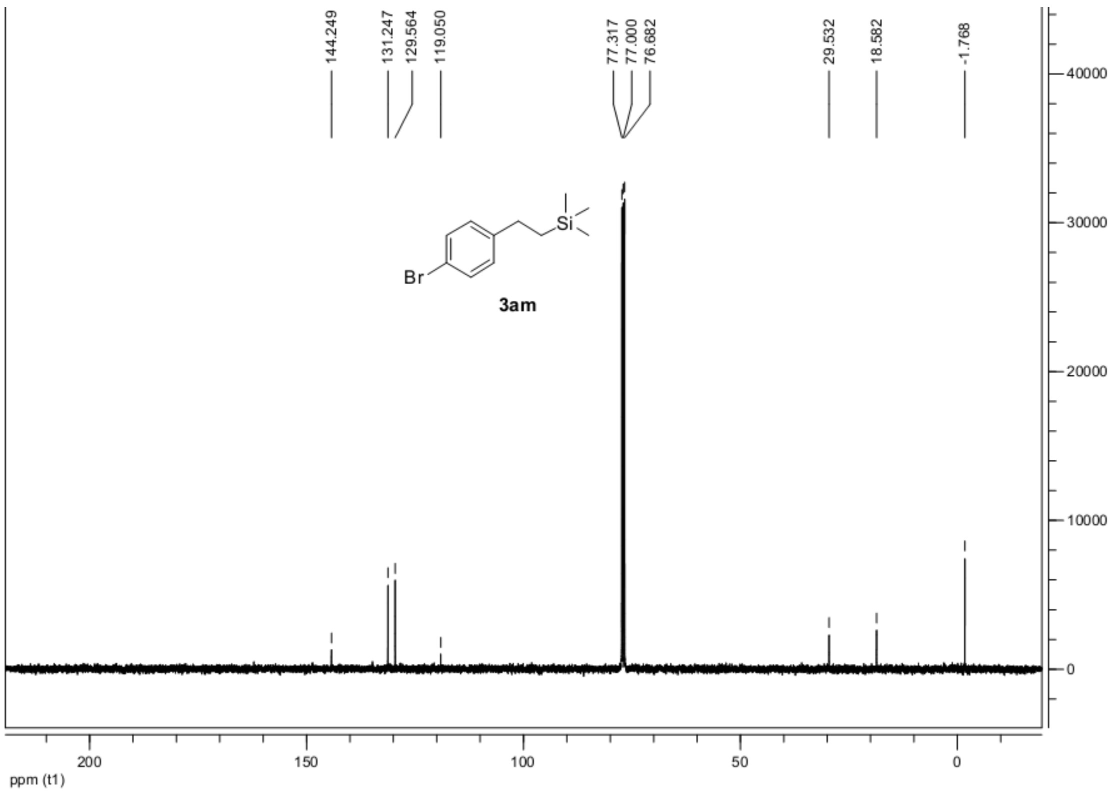

Supplementary Figure 69 | ^{13}C NMR spectrum of (4-bromophenethyl)trimethylsilane (3am). (100 MHz, $CDCl_3$, 298 K).

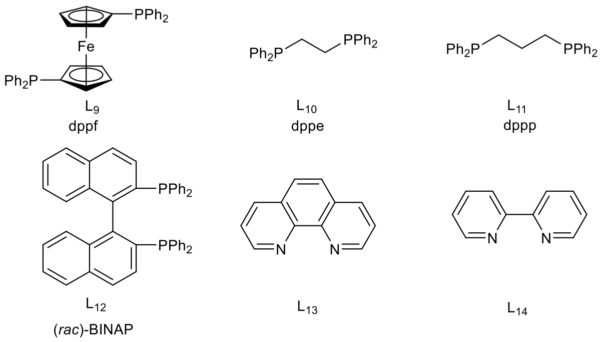

Supplementary Figure 70 | Bidentate ligands. Other bidentate ligands listed above were screened during the process of optimization for C(*sp*^{2})-C(*sp*^{3}) cross-coupling of 4-methoxybromobenzene (**1a**) and *n*-BuLi (**2a**).

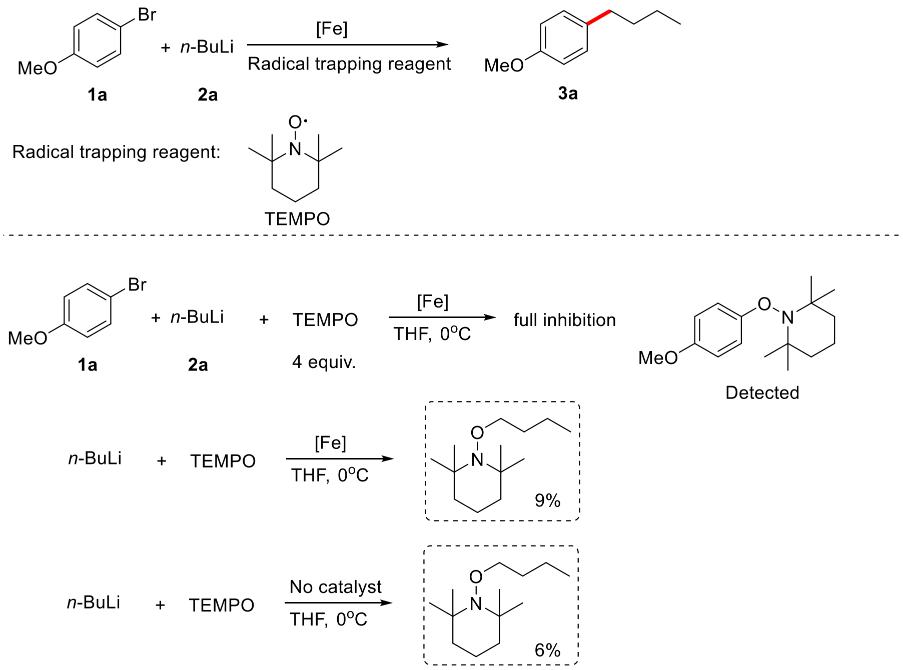

Supplementary Figure 71 | Control experiments. TEMPO as radical trapping reagent was added to quench the iron-catalysed cross-coupling of 4-methoxybromobenzene (**1a**) and *n*-BuLi (**2a**).

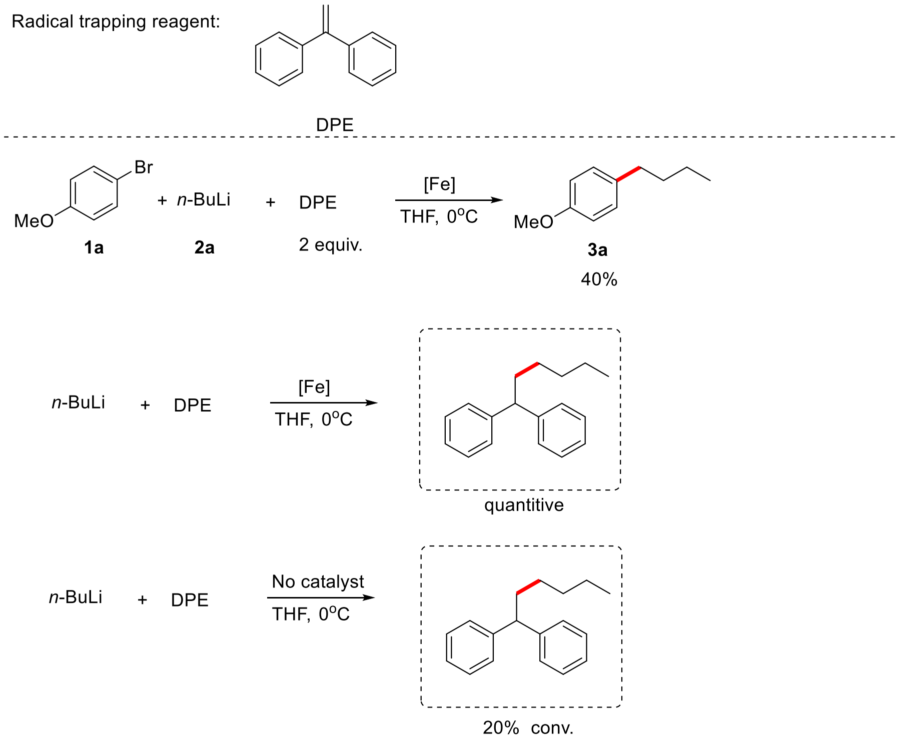

Supplementary Figure 72 | Control experiments. DPE as radical trapping reagent was added to quench the iron-catalysed cross-coupling of 4-methoxybromobenzene (**1a**) and *n*-BuLi (**2a**).

Supplementary Tables

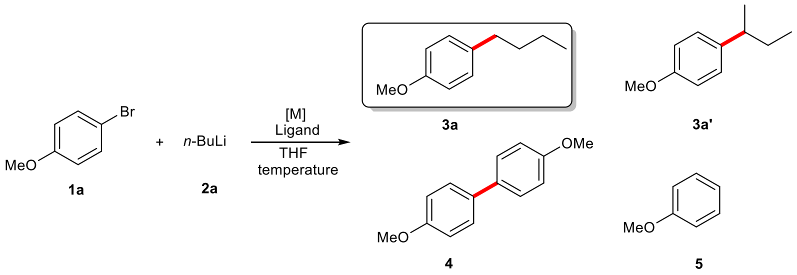

Entry	[M] (mol%)	Ligand (mol%)	T/^{o}C	Conv. (%)	3a/3a'/4/5†
1	–	–	0	5	0/0/0/>90
2	$PdCl_2$ (6)	–	0	full	29/2/54/5
3	$NiCl_2$ (6)	–	0	full	58/1/26/15
4	$CoCl_2$ (6)	–	0	full	13/0/84/3
5	$CuCl_2$ (6)	–	0	70%	79/3/10/8
6	$FeCl_2$ (10)	L_9 (20)	r.t.	full	17/5/33/45
7	$FeCl_2$ (10)	L_{10} (20)	r.t.	full	13/0/23/55
8	$FeCl_2$ (10)	L_{11} (20)	r.t.	full	11/0/29/50
9	$FeCl_2$ (10)	L_{12} (20)	r.t.	full	9/3/35/53
10	$FeCl_2$ (10)	L_{13} (20)	r.t.	full	5/0/28/67
11	$FeCl_2$ (10)	L_{14} (20)	r.t.	67%	7/0/15/78

Supplementary Table 1 | Other optimization results for C(sp^2)-C(sp^3) Cross-Coupling of 4-methoxybromobenzene (**1a**) and *n*-BuLi (**2a**).

Element / Sample	Pd	Co	Ni	Zn	Cu
	μg	μg	μg	μg	μg
$FeCl_3$/g	N.D	6	59	N.D	N.D

N.D: Not detected.

m[Co] (μg) = m[$FeCl_3$] $\times 10^{-3}$ (mg) $\times 6 = 1.14 \times 10^{-2}$ μg

m[Ni] (μg) = m[$FeCl_3$] $\times 10^{-3}$ (mg) $\times 59 = 0.1121$ μg

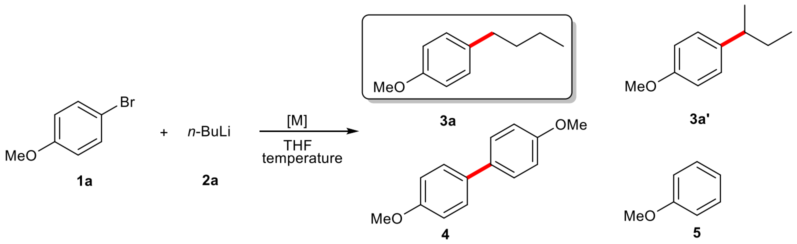

Entry	[M]/ μg	T/°C	Conv. (%)	3a/3a'/4/5†
1	Co/1.14×10^{-2}	0	5	0/0/trace/>90
2	Ni/0.1121	0	5	trace/0/0/>90
3	−	0	5	0/0/0/>90

Supplementary Table 2 | ICP−AES (Inductively Coupled Plasma−Mass Spectroscopy) analysis of various metals in iron(III) chloride and mimic contaminant results for C(sp^2)-C(sp^3) Cross-Coupling of 4-methoxybromobenzene (**1a**) and *n*-BuLi (**2a**).

Supplementary Methods

General information

All reactions were carried out under an atmosphere of dry argon with the rigid exclusion of air and moisture using standard Schlenk techniques or in a glovebox unless otherwise specified. ^{1}H and ^{13}C NMR spectras were recorded on a Bruker DPX 400.19 spectrometer at 400 MHz and 100 MHz, respectively. Chemical shifts (^{1}H and ^{13}C) are reported in parts per million and referenced to the residual solvent peak (for $CDCl_3$, δ = 7.27 ppm, 77.0 ppm respectively). The following designations are used to describe multiplicities: s (singlet), d (doublet), t (triplet), q (quartet), m (multiplet), br (broad), app (apparent). When rotational isomers are present, the major rotational isomer is reported, as are any clearly differentiated signals arising from the minor rotational isomer for ^{1}H NMR spectra. For ^{13}C NMR spectra, peaks are for major rotational isomer only unless otherwise noted. GC/MS was performed on an Agilent 7890B system (HP-5ms column) with an Agilent 5977A MSD. All organic solvents were freshly distilled from Na or CaH_2 immediately prior to use. All other chemicals were purchased from either Aldrich or Acros Chemical Co. and used as received unless otherwise specified.

Typical procedures for Iron-Catalysed Cross-Coupling of Organolithium Compounds

Iron-catalysed cross-coupling of 4-methoxybromobenzene (1a) and *n*-BuLi (2a).

An oven-dried vial was equipped with a magnetic stirring bar and transferred into a glove box. [$(FeCl_3)_2(TMEDA)_3$] (3.96 mg, 0.006 mmol, 3 mol%) was charged and 4-methoxybromobenzene (0.2 mmol) and THF (1 mL) were added subsequently. Then, the vial was sealed with a rubber stopper and taken out from the glove box. At 0 ^{o}C, *n*-BuLi (0.30 mmol, 1.6 M or 2.4 M in hexane, diluted with THF to a final concentration of 0.35 M) was added by a syringe pump in 1 h. After the addition was completed, the reaction mixture was stirred at 0 ^{o}C for 1 h. Then, a saturated solution of aqueous NH_4Cl was added and the reaction mixture was extracted three times with CH_2Cl_2. The organic phases were collected, and the solvent evaporation under reduced pressure afforded the crude product, which was then purified by preparative thin layer chromatography.

Iron-catalysed release-catch ethylene coupling of 4-methoxybromobenzene (1a) with isopropyllithium.

An oven-dried vial was equipped with a magnetic stirring bar and transferred into a glove box. [$(FeCl_3)_2(TMEDA)_3$] (3.96 mg, 0.006 mmol, 3 mol%) was charged and the vial was sealed with a rubber stopper. Then, the vial was taken out from the glovebox. At −78 ^{o}C, isopropyllithium (0.50 mmol, 0.7 M in pentane) was added slowly into a solution of 4-methoxybromobenzene (0.2 mmol) in THF (1.0 mL), Subsequently, the mixture was added using a syringe pump in 1.0 h to a solution of [$(FeCl_3)_2(TMEDA)_3$]

(3 mol%) in THF (1.0 mL) at 22 °C. After the addition was completed, the reaction mixture was stirred overnight. Then, a saturated aqueous solution of NH_4Cl was added and the reaction mixture was extracted three times with CH_2Cl_2. The organic phases were collected, and the solvent evaporation under reduced pressure afforded the crude product, which was then purified by preparative thin layer chromatography.

Iron-catalysed cross-coupling of 1-bromo-3-phenylpropane with *n*-BuLi (2a).

An oven-dried vial was equipped with a magnetic stirring bar and transferred into a glove box. $[(FeCl_3)_2(TMEDA)_3]$ (3.96 mg, 0.006 mmol, 3 mol%) was charged and 1-bromo-3-phenylpropane (0.2 mmol) and THF (1 mL) were added subsequently. Then, the vial was sealed with a rubber stopper and taken out from the glove box. At 22 °C, *n*-BuLi (0.30 mmol, 1.6 M or 2.4 M in hexane, diluted with THF to a final concentration of 0.35 M) was added by a syringe pump in 1 h. After the addition was completed, the reaction mixture was stirred at 22 °C overnight. Then, a saturated aqueous solution of NH_4Cl was added and the reaction mixture was extracted three times with CH_2Cl_2. The organic phases were collected, and the solvent evaporation under reduced pressure afforded the crude product, which was then purified by preparative thin layer chromatography.

Gram-Scale reactions

Procedure for synthesis of 3a in a multi-gram scale.

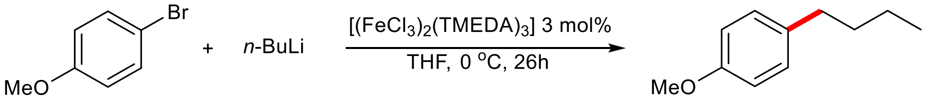

1a **2a** **3a**

A 250 mL oven-dried round bottom flask was equipped with a magnetic stirring bar and transferred into a glove box. $[(FeCl_3)_2(TMEDA)_3]$ (198 mg, 0.3 mmol, 3 mol%) was charged. Then, the flask was sealed with a rubber stopper and taken out from the glove box. Subsequently, 4-methoxybromobenzene (1.25 mL, 10 mmol), which was distilled under reduced pressure, and freshly distilled THF (50 mL) were added at room temperature. The flask was transferred to 0 °C (ice bath) and diluted *n*-BuLi (6.25 mL, 15 mmol, 2.4 M in hexane, diluted with THF to a final concentration of 0.35 M) was added by a syringe pump in 25 h. After the addition was completed, the reaction mixture was stirred at 0 °C for 1 h. Then, a saturated aqueous solution of NH_4Cl (100 mL) was added and the reaction mixture was extracted three times with CH_2Cl_2 (300 mL). The organic phases were collected, and the solvent evaporation under reduced pressure afforded the crude product, which was then purified by flash column chromatography (100% hexane) to afford **3a** (75%) as a colorless oil.

Procedure for synthesis of $3a_{THF}$ in a multi-gram scale.

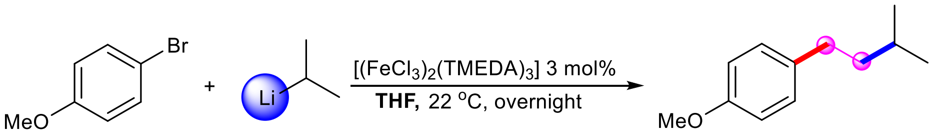

1a **$3a_{THF}$**

A 250 mL oven-dried round bottom flask was equipped with a magnetic stirring bar and transferred into a glove box. [$(FeCl_3)_2(TMEDA)_3$] (198 mg, 0.3 mmol, 3 mol%) was charged. Then, the flask was sealed with a rubber stopper and taken out from the glove box. Subsequently, 4-methoxybromobenzene (1.25 mL, 10 mmol), which was distilled under reduced pressure, and freshly distilled THF (10 mL) were added at room temperature. At –78 °C (dry ice/acetone), isopropyllithium (35.7 mL, 25 mmol, 0.7 M in pentane) was added slowly into THF (100 mL). The mixture was added immediately using a syringe pump in 5 h to the flask at 22 °C. After the addition was completed, the reaction mixture was stirred overnight. Then, a saturated aqueous solution of NH_4Cl (100 mL) was added and the reaction mixture was extracted three times with CH_2Cl_2 (300 mL). The organic phases were collected, and the solvent evaporation under reduced pressure afforded the crude product, which was then purified by flash column chromatography (100% hexane) to afford **$3a_{THF}$** (63%) as a colorless oil.

Procedure for synthesis of 3aa in a multi-gram scale.

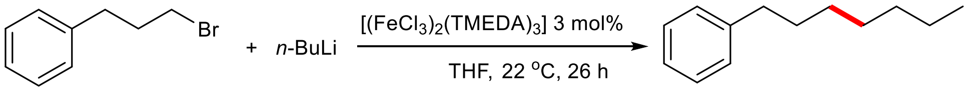

1aa **2a** **3aa**

A 250 mL oven-dried round bottom flask was equipped with a magnetic stirring bar and transferred into a glove box. [$(FeCl_3)_2(TMEDA)_3$] (198 mg, 0.3 mmol, 3 mol%) was charged. Then, the flask was sealed with a rubber stopper and taken out from the glove box. Subsequently, 1-bromo-3-phenylpropane (1.52 mL, 10 mmol), which was distilled under reduced pressure, and freshly distilled THF (50 mL) were added at room temperature. At 22 °C, *n*-BuLi (6.25 mL, 15 mmol, 2.4 M in hexane, diluted with THF to a final concentration of 0.35 M) was added by a syringe pump in 25 h. After the addition was completed, the reaction mixture was stirred at 22 °C for 1 h. Then, a saturated aqueous solution of NH_4Cl (100 mL) was added and the reaction mixture was extracted three times with CH_2Cl_2 (300 mL). The organic phases were collected, and the solvent evaporation under reduced pressure afforded the crude product, which was then purified by flash column chromatography (100% hexane) to afford **3aa** (67%) as a colorless oil.

Control experiments

To evaluate the involvement of radical intermediates and the role of the iron catalyst, several control experiments were designed and conducted. We attempted to use some radical trapping regents to quench the radicals that might be involved, such as TEMPO and 1,1-diphenylethylene. It was found that TEMPO resulted in full

inhibition of the cross-coupling reaction under standard conditions. However, *n*-BuLi was also consumed by TEMPO (Figure 71). When 1,1-diphenylethylene was introduced to trap the possible radicals being resulted from the reaction mixture, the product 1,1-diphenylhexane from 1,1-diphenylethylene coupled with *n*-BuLi was isolated (Figure 72). Although direct evidence has not been obtained, it seems very likely that radical species are involved in the iron-catalysed cross-coupling process.

Preparation of Organolithium Reagents

Representative procedure for the generation of organolithium reagents: pentyllithium, heptyllithium, and octyllithium.

To a dry Schlenk flask equipped with a magnetic stirring bar was added freshly prepared lithium slots (277.6 mg, 40 mmol) and Et_2O (5 mL) under argon. The mixture was cooled to –20 ^{o}C and a solution of pentyl bromide (1.24 mL, 10 mmol) in Et_2O (5.0 mL) was added slowly over 120 min using a syringe pump. After the addition, the mixture was allowed to warm to room temperature and stirred for an additional 90 min.

Cyclopropyllithium[1]

Cyclopropyllithium was prepared in accordance with a previously reported procedure.

(9*H*-fluoren-9-yl)lithium[2]

(9*H*-fluoren-9-yl)lithium was prepared in accordance with a previously reported procedure.

Analytical Data of Compounds

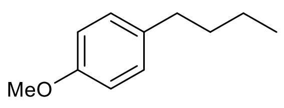

1-Butyl-4-methoxybenzene (3a)[3]. ^{1}H NMR (400 MHz, $CDCl_3$) δ 7.10 (d, *J* = 8.5 Hz, 2H), 6.83 (d, *J* = 8.6 Hz, 2H), 3.79 (s, 3H), 2.55 (t, *J* = 8.0 Hz, 2H), 1.61-1.57 (m, 0.75H), 1.56-1.53 (m, 1.25H), 1.40-1.30 (m,2H), 0.92 (t, *J* = 7.3 Hz, 3H). ^{13}C NMR (100 MHz, $CDCl_3$) δ 157.60, 135.02, 129.23, 113.64, 55.24, 34.71, 33.89, 22.29, 13.94.

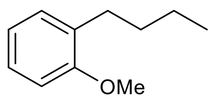

1-Butyl-2-methoxybenzene (3b)[4]. ^{1}H NMR (400 MHz, $CDCl_3$) δ 7.18-7.12 (m, 2H), 6.90-6.83 (m, 2H), 3.82 (s, 3H), 2.61 (t, *J* = 8.0 Hz, 2H), 1.60-1.58 (m, 1H), 1.55-1.52 (m, 1H), 1.41-1.31 (m, 2H), 0.93 (t, *J* = 7.3 Hz, 3H). ^{13}C NMR (100 MHz, $CDCl_3$) δ 157.43, 131.31, 129.71, 126.71, 120.27, 110.20, 55.25, 32.05, 29.82, 22.65, 14.01.

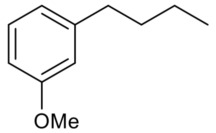

1-Butyl-3-methoxybenzene (3c)[4]. ^{1}H NMR (400 MHz, $CDCl_3$) δ 7.19 (td, *J* = 7.5 Hz, 1.2 Hz, 1H), 6.78 (d, *J* = 7.7 Hz, 2H), 6.73 (s, 1.5H), 6.71 (s, 0.5H), 3.80 (s, 3H), 2.59 (t, *J* = 8.0 Hz, 2H), 1.63-1.62 (m, 2H), 1.40-1.31 (m, 2H), 0.92 (t, *J* = 7.3 Hz, 3H). ^{13}C NMR (100 MHz, $CDCl_3$) δ 159.53, 144.58, 129.12, 120.86, 114.17, 110.77, 55.10, 35.70, 33.53, 22.36, 13.94.

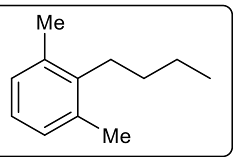

2-Butyl-1,3-dimethylbenzene (3e)[5]. ^{1}H NMR (400 MHz, $CDCl_3$) δ 6.99 (s, 3H), 2.62-2.58 (m, 2H), 2.32 (s, 6H), 1.46-1.42 (m, 4H), 0.97 (t, *J* = 6.8 Hz, 3H). ^{13}C NMR (100 MHz, $CDCl_3$) δ 139.73, 135.91, 128.00, 125.37, 31.25, 29.45, 23.31, 19.77, 13.94.

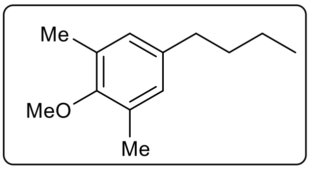

5-Butyl-2-methoxy-1,3-dimethylbenzene (3f). ^{1}H NMR (400 MHz, $CDCl_3$) δ 6.84 (s, 2H), 3.72 (s, 3H), 2.51 (t, *J* = 8.0 Hz, 2H), 2.28 (s, 6H), 1.62-1.54 (m, 2H), 1.41-1.32 (m, 2H), 0.94 (t, *J* = 7.3 Hz, 3H). ^{13}C NMR (100 MHz, $CDCl_3$) δ 154.81, 138.18, 130.32, 128.65, 59.66, 34.97, 33.82, 22.46, 16.03, 13.96. HRMS (EI): calculated for $C_{13}H_{20}O$ [M+]: 192.15087; found: 192.15132.

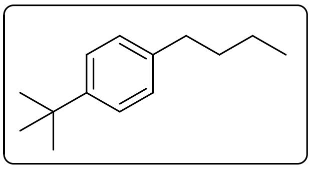

1-*tert*-Butyl-4-butylbenzene (3g)[4]. ^{1}H NMR (400 MHz, $CDCl_3$) δ 7.30 (d, *J* = 8.2 Hz, 2H), 7.12 (d, *J* = 8.1 Hz, 2H), 2.58 (t, *J* = 8.0 Hz, 2H), 1.63-1.57 (m, 2H), 1.41-1.34 (m, 2H), 1.31 (s, 9H), 0.93 (t, *J* = 7.3 Hz, 3H). ^{13}C NMR (100 MHz, $CDCl_3$) δ 148.26, 139.82, 128.01, 125.07, 35.09, 34.30, 33.64, 31.41, 22.47, 13.98.

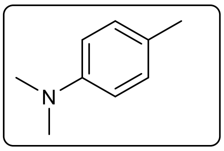

Dimethyl-*p*-toluidine (3i)[6]. ^{1}H NMR (400 MHz, $CDCl_3$) δ 7.07 (d, *J* = 8.2 Hz, 2H), 6.72 (d, *J* = 8.6 Hz, 2H), 2.91 (s, 6H), 2.27 (s, 3H). ^{13}C NMR (100 MHz, $CDCl_3$) δ 148.60, 129.50, 126.51, 113.42, 41.22, 20.25.

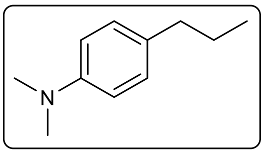

***N,N*-Dimethyl-4-propylaniline (3j)[6].** ^{1}H NMR (400 MHz, $CDCl_3$) δ 7.08 (d, *J* = 6.5 Hz, 2H), 6.73 (d, *J* = 6.6 Hz, 2H), 2.92 (s, 6H), 2.51 (t, *J* = 7.6 Hz, 2H), 1.65-1.56 (m, 2H), 0.94 (t, *J* = 7.3 Hz, 3H). ^{13}C NMR (100 MHz, $CDCl_3$) δ 148.73, 131.43, 129.02, 113.19, 41.12, 37.04, 24.83, 13.86.

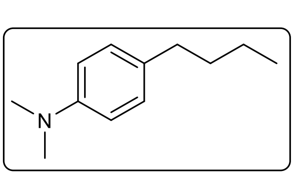

4-Butyl-*N,N*-dimethylaniline (3k)[3]. ^{1}H NMR (400 MHz, $CDCl_3$) δ 7.08 (d, *J* = 8.4 Hz, 2H), 6.72 (d, *J* = 8.5 Hz, 2H), 2.92 (s, 6H), 2.54 (t, *J* = 7.7 Hz, 2H), 1.62-1.54 (m,

2H), 1.42-1.32 (m, 2H), 0.94 (t, *J* = 7.3 Hz, 3H). ^{13}C NMR (100 MHz, $CDCl_3$) δ 148.92, 131.26, 128.93, 113.03, 40.97, 34.57, 33.96, 22.35, 13.97.

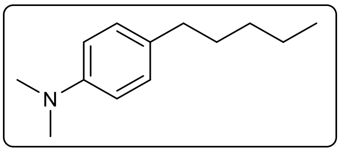

***N,N*-Dimethyl-4-pentylaniline (3l).** ^{1}H NMR (400 MHz, $CDCl_3$) δ 7.09 (d, *J* = 8.6 Hz, 2H), 6.73 (d, *J* = 8.6 Hz, 2H), 2.93 (s, 6H), 2.53 (t, *J* = 8.0 Hz, 2H), 1.64-1.56 (m, 2H), 1.38-1.32 (m, 4H), 0.91 (t, *J* = 6.9 Hz, 3H). ^{13}C NMR (100 MHz, $CDCl_3$) δ 148.79, 131.45, 128.93, 113.09, 41.03, 34.87, 31.53, 31.50, 22.57, 14.05. HRMS (EI): calculated for $C_{13}H_{21}N$ [M+]: 191.16685; found: 191.16705.

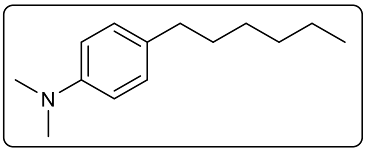

4-Hexyl-*N,N*-dimethylaniline (3m)[7]. ^{1}H NMR (400 MHz, $CDCl_3$) δ 7.08 (d, *J* = 8.5 Hz, 2H), 6.73 (d, *J* = 8.5 Hz, 2H), 2.92 (s, 6H), 2.53 (t, *J* = 8.0 Hz, 2H), 1.62-1.55 (m, 2H), 1.36-1.32 (m, 6H), 0.90 (t, *J* = 7.4 Hz, 3H). ^{13}C NMR (100 MHz, $CDCl_3$) δ 148.81, 131.44, 128.93, 113.09, 41.03, 34.91, 31.78, 31.78, 29.03, 22.63, 14.11.

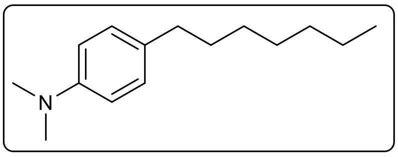

4-Heptyl-*N,N*-dimethylaniline (3n). ^{1}H NMR (400 MHz, $CDCl_3$) δ 7.10 (d, *J* = 8.6 Hz, 2H), 6.74 (d, *J* = 8.6 Hz, 2H), 2.94 (s, 6H), 2.55 (t, *J* = 8.0 Hz, 2H), 1.64-1.57 (m, 2H), 1.35-1.31 (m, 8H), 0.92 (t, *J* = 6.9 Hz, 3H). ^{13}C NMR (100 MHz, $CDCl_3$) δ 148.82, 131.38, 128.91, 113.05, 40.99, 34.90, 31.84, 31.84, 29.31, 29.23, 22.68, 14.10. HRMS (EI): calculated for $C_{15}H_{25}N$ [M+]: 219.19815; found: 219.19866.

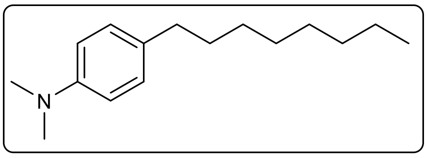

***N,N*-Dimethyl-4-octylaniline (3o).** ^{1}H NMR (400 MHz, $CDCl_3$) δ 7.08 (d, *J* = 8.7 Hz, 2H), 6.74 (d, *J* = 8.6 Hz, 2H), 2.93 (s, 6H), 2.53 (t, *J* = 8.0 Hz, 2H), 1.62-1.55 (m, 2H), 1.31-1.28 (m, 10H), 0.90 (t, *J* = 6.9 Hz, 3H). ^{13}C NMR (100 MHz, $CDCl_3$) δ 148.72, 131.63, 128.95, 113.18, 41.09, 34.92, 31.90, 31.82, 29.52, 29.36, 29.29, 22.67, 14.10. HRMS (EI): calculated for $C_{16}H_{27}N$ [M+]: 233.21380; found: 233.21378.

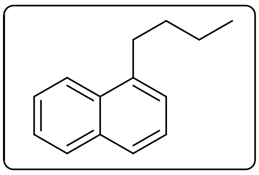

1-Butylnaphthalene (3p)[3]. ^{1}H NMR (400 MHz, $CDCl_3$) δ 8.05 (d, *J* = 8.1 Hz, 1H), 7.85 (d, *J* = 8.2 Hz, 1H), 7.70 (d, *J* = 8.1 Hz, 1H), 7.53-7.44 (m, 2H), 7.39 (t, *J* = 8.0 Hz, 1H), 7.32 (d, *J* = 6.8 Hz, 1H), 3.08 (t, *J* = 8.0 Hz, 2H), 1.78-1.70 (m, 2H), 1.51-1.41 (m, 2H), 0.98 (t, *J* = 7.3 Hz, 3H). ^{13}C NMR (100 MHz, $CDCl_3$) δ 131.89, 133.86, 138.98, 128.71, 126.35, 125.83, 125.58, 125.51 125.32, 123.90, 33.00, 32.80, 22.87, 14.00.

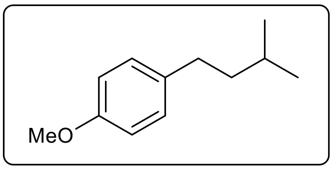

1-isopentyl-4-methoxybenzene ($3a_{THF}$). ^{1}H NMR (400 MHz, $CDCl_3$) δ 7.10 (d, *J* = 8.7 Hz, 2H), 6.82 (d, *J* = 8.8 Hz, 2H), 3.79 (s, 3H), 2.55 (t, *J* = 8.0 Hz, 2H), 1.56-1.57 (m, 1H), 1.49-1.44 (m, 2H), 0.92 (d, *J* = 6.5 Hz, 6H). ^{13}C NMR (100 MHz, $CDCl_3$) δ 157.53, 135.19, 129.16, 113.65, 55.24, 41.07, 32.81, 27.58, 22.52. HRMS (EI): calculated for $C_{12}H_{18}O$ [M+]: 178.13522; found: 178.13535.

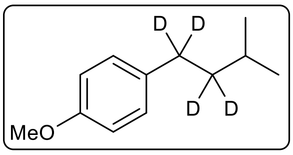

1-methoxy-4-(3-methylbutyl-1,1,2,2-*d*$_4$)benzene ($3a_{THF-d8}$). ^{1}H NMR (400 MHz, $CDCl_3$) δ 7.10 (d, *J* = 8.7 Hz, 2H), 6.82 (d, *J* = 8.8 Hz, 2H), 3.79 (s, 3H), 2.55 (t, *J* = 8.0 Hz, 2H), 1.56-1.57 (m, 1H), 1.49-1.44 (m, 2H), 0.92 (d, *J* = 6.5 Hz, 6H). ^{2}H NMR (400 MHz, $CHCl_3$) δ 2.50 (s, 2D), 1.42 (s, 2D). HRMS (EI): calculated for $C_{12}H_{14}D_4O$ [M+]: 182.16032; found: 182.16034.

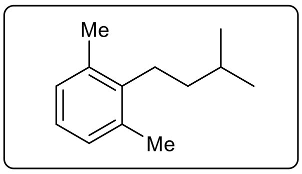

2-Isopentyl-1,3-dimethylbenzene ($3c_{THF}$). ^{1}H NMR (400 MHz, $CDCl_3$) δ 6.99 (s, 3H), 2.61-2.57 (m, 2H), 2.31 (s, 6H), 1.73-1.63 (m, 1H), 1.36-1.30 (m, 2H), 0.98 (d, *J* = 6.6 Hz, 6H). ^{13}C NMR (100 MHz, $CDCl_3$) δ 139.85, 135.80, 128.01, 125.35, 38.03, 28.85, 27.59, 22.45, 19.67. HRMS (EI): calculated for $C_{13}H_{20}$ [M+]: 176.15650; found: 176.15607.

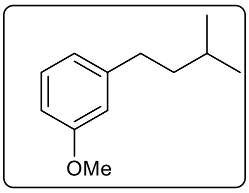

1-Isopentyl-3-methoxybenzene (3d$_{THF}$). ^{1}H NMR (400 MHz, $CDCl_3$) δ 7.19 (t, *J* = 7.7 Hz, 1H), 6.78 (d, *J* = 7.5 Hz, 1H), 6.73 (s, 1.5H), 6.71 (s, 0.5H), 3.80 (s, 3H), 2.58 (t, *J* = 7.9 Hz, 2H), 1.59-1.58 (m, 1H), 1.53-1.47 (m, 2H), 0.93 (d, *J* = 6.5 Hz, 2H). ^{13}C NMR (100 MHz, $CDCl_3$) δ 159.60, 144.83, 129.16, 120.79, 114.12, 110.74, 55.11, 40.69, 33.83, 27.67, 22.52. HRMS (EI): calculated for $C_{12}H_{18}O$ [M+]: 178.13522; found: 178.13493.

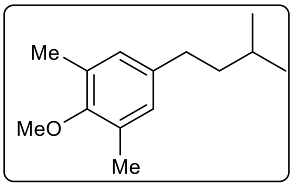

5-Isopentyl-2-methoxy-1,3-dimethylbenzene (3e$_{THF}$). ^{1}H NMR (400 MHz, $CDCl_3$) δ 6.83 (s, 2H), 3.71 (s, 3H), 2.52-2.47 (m, 2H), 2.26 (s, 6H), 1.63-1.57 (m, 1H), 1.50-1.44 (m, 2H), 0.93 (d, *J* = 6.5 Hz, 6H). ^{13}C NMR (100 MHz, $CDCl_3$) δ 154.79, 138.39, 130.38, 128.59, 59.69, 41.05, 33.09, 27.78, 22.54, 16.04. HRMS (EI): calculated for $C_{14}H_{22}O$ [M+]: 206.16652; found: 206.16655.

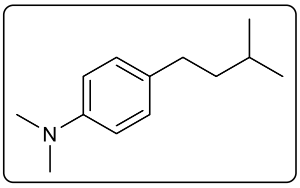

4-Isopentyl-*N*,*N*-dimethylaniline (3f$_{THF}$). ^{1}H NMR (400 MHz, $CDCl_3$) δ 7.07 (d, *J* = 8.3 Hz, 2H), 6.72 (d, *J* = 8.5 Hz, 2H), 2.92 (s, 6H), 2.53 (t, *J* = 7.3 Hz, 2H), 1.64-1.54 (m, 1H), 1.50-1.44 (m, 2H), 0.93 (dd, *J* = 6.5 Hz, 1.4 Hz, 6H). ^{13}C NMR (100 MHz, $CDCl_3$) δ 128.89, 113.18, 41.13, 32.67, 27.60, 22.56. HRMS (EI): calculated for $C_{13}H_{21}N$ [M+]: 191.16685; found: 191.16744.

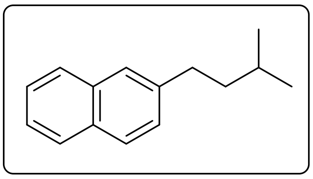

2-Isopentylnaphthalene (3g$_{THF}$). ^{1}H NMR (400 MHz, $CDCl_3$) δ 7.83-7.76 (m, 3H), 7.63 (s, 1H), 7.48-7.36 (m, 2H), 7.35 (d, *J* = 7.5 Hz, 1H), 2.79 (t, *J* = 8.0 Hz, 2H), 1.69-1.59 (m, 3H), 0.98 (d, *J* = 6.1 Hz, 6H). ^{13}C NMR (100 MHz, $CDCl_3$) δ 140.61, 133.65, 131.88, 127.72, 127.57, 127.41, 127.35, 126.16, 125.78, 124.95, 40.69, 33.93, 27.70, 22.56. HRMS (EI): calculated for $C_{15}H_{18}$ [M+]: 198.14030; found: 198.14046.

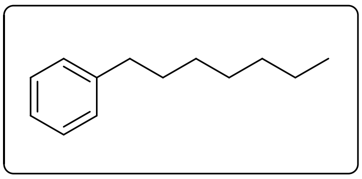

Heptylbenzene (3aa)[8]. ^{1}H NMR (400 MHz, $CDCl_3$) δ 7.31-7.27 (m, 2H), 7.21-7.18 (m, 3H), 2.61 (t, *J* = 8.0 Hz, 2H), 1.64-1.61 (m, 2H), 1.33-1.28 (m, 8H), 0.89 (t, *J* = 6.8 Hz, 3H). ^{13}C NMR (100 MHz, $CDCl_3$) δ 142.96, 128.38, 128.19, 125.52, 35.99, 31.82, 31.54, 29.31, 29.18, 22.66, 14.10.

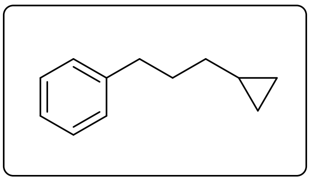

(3-Cyclopropylpropyl)benzene (3ab). ^{1}H NMR (400 MHz, $CDCl_3$) δ 7.29-7.25 (m, 2H), 7.19-7.15 (m, 3H), 2.63 (t, *J* = 8.0 Hz, 2H), 1.76-1.68 (m, 2H), 1.23 (dd, *J* = 14.8Hz, 7.1Hz, 2H), 0.73-0.63 (m, 1H), 0.42-0.37 (m, 2H), 0.012- -0.01 (m, 2H). ^{13}C NMR (100 MHz, $CDCl_3$) δ 142.87, 128.36, 128.21, 125.54, 35.77, 34.34, 31.41, 10.74, 4.37. HRMS (EI): calculated for $C_{12}H_{16}$ [M+]: 160.12465; found: 160.12428.

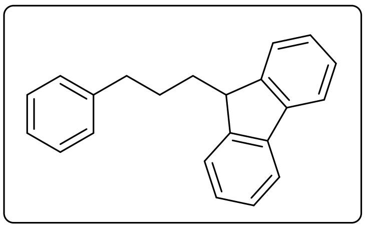

9-(3-Phenylpropyl)-9H-fluorene (3ac). ^{1}H NMR (400 MHz, $CDCl_3$) δ 7.77 (d, *J* = 7.4 Hz, 2H), 7.50 (d, *J* = 7.3 Hz, 2H), 7.39 (t, *J* = 7.4 Hz, 2H), 7.32 (t, *J* = 7.4 Hz, 2H), 7.27 (t, *J* = 6.5 Hz, 2H), 7.18 (t, *J* = 7.4 Hz, 1H), 7.11 (d, *J* = 7.7 Hz, 2H), 4.02 (t, *J* = 5.7 Hz,1H), 2.59 (t, *J* = 7.6 Hz, 2H), 2.12-2.06 (m, 2H), 1.57-1.49 (m, 2H). ^{13}C NMR (100 MHz, $CDCl_3$) δ 147.25, 142.15, 141.11, 128.35, 128.20, 126.87, 126.79, 125.66, 124.28, 119.78, 47.25, 36.10, 32.50, 27.16. HRMS (EI): calculated for $C_{22}H_{20}$ [M+]: 284.15595; found: 284.15622.

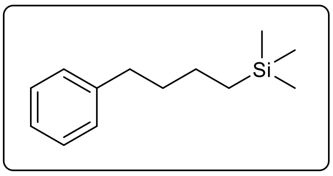

Trimethyl(4-phenylbutyl)silane (3ad). ^{1}H NMR (400 MHz, $CDCl_3$) δ 7.30-7.25 (m, 2H), 7.20-7.15 (m, 3H), 2.61 (t, *J* = 8.0 Hz, 2H), 1.68-1.60 (m, 2H), 1.40-1.32 (m, 2H), 0.55-0.51 (m, 2H), -0.02 (s, 9H). ^{13}C NMR (100 MHz, $CDCl_3$) δ 142.93, 128.38,

128.20, 125.50, 35.70, 35.41, 23.67, 16.50, −1.65. HRMS (EI): calculated for $C_{13}H_{22}Si$ [M+]: 206.14853; found: 206.14874.

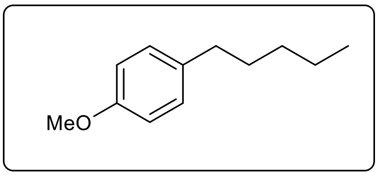

1-Methoxy-4-pentylbenzene (3ae)[9]. ^{1}H NMR (400 MHz, $CDCl_3$) δ 7.10 (d, *J* = 8.5 Hz, 2H), 6.83 (d, *J* = 8.5 Hz, 2H), 3.79 (s, 3H), 2.55 (t, *J* = 8.0 Hz, 2H), 1.63-1.55 (m, 2H), 1.35-1.30 (m, 4H), 0.90 (t, *J* = 6.8 Hz, 3H). ^{13}C NMR (100 MHz, $CDCl_3$) δ 157.55, 135.04, 129.22, 113.60, 55.22, 34.99, 31.45, 31.45, 22.54, 14.03.

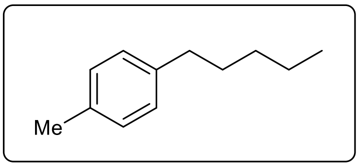

1-Methyl-4-pentylbenzene (3af)[10]. ^{1}H NMR (400 MHz, $CDCl_3$) δ 7.09 (s, 4H), 2.57 (t, *J* = 8.0 Hz, 2H), 2.33 (s, 3H), 1.62-1.58 (m, 2H), 1.35-1.32 (m, 4H), 0.90 (t, *J* = 6.9 Hz, 3H). ^{13}C NMR (100 MHz, $CDCl_3$) δ 139.86, 134.92, 128.89, 128.26, 35.48, 31.52, 31.33, 22.55, 20.98, 14.03.

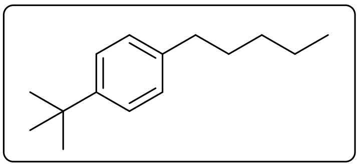

1-*tert*-Butyl-4-pentylbenzene (3ag). ^{1}H NMR (400 MHz, $CDCl_3$) δ 7.32 (d, *J* = 8.1 Hz, 2H), 7.14 (d, *J* = 8.1 Hz, 2H), 2.59 (t, *J* = 8.0 Hz, 2H), 1.67-1.60 (m, 2H), 1.37-1.35 (m, 4H), 1.34 (bs, 9H), 0.92 (t, *J* = 6.7 Hz, 3H). ^{13}C NMR (100 MHz, $CDCl_3$) δ 148.27, 139.88, 128.00, 125.08, 35.39, 34.31, 31.65, 31.42, 31.19, 22.57, 14.04. HRMS (EI): calculated for $C_{15}H_{24}$ [M+]: 204.18725; found: 204.18720.

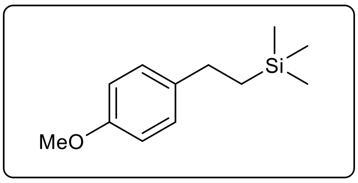

(4-Methoxyphenethyl)trimethylsilane (3ah). ^{1}H NMR (400 MHz, $CDCl_3$) δ 7.12 (d, *J* = 8.6 Hz, 2H), 6.82 (d, *J* = 8.6 Hz, 2H), 3.79 (s, 3H), 2.60-2.55 (m, 2H), 0.86-0.82 (m, 2H), 0.01 (s, 9H). ^{13}C NMR (100 MHz, $CDCl_3$) δ 157.49, 137.41, 128.60, 113.65, 55.25, 29.09, 18.87, −1.74. HRMS (EI): calculated for $C_{12}H_{20}OSi$ [M+]: 208.12779; found: 208.12794.

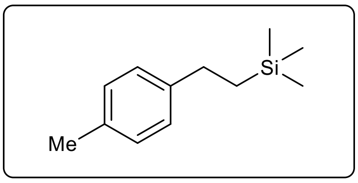

Trimethyl(4-methylphenethyl)silane (3ai). ^{1}H NMR (400 MHz, $CDCl_3$) δ 7.09 (s, 4H), 2.61-2.57 (m, 2H), 2.32 (s, 3H), 0.88-0.83 (m, 2H), 0.02 (s, 9H). ^{13}C NMR (100 MHz, $CDCl_3$) δ 142.27, 134.81, 128.93, 127.62, 29.55, 20.96, 18.82, −1.75. HRMS (EI): calculated for $C_{12}H_{20}Si$ [M+]: 192.13288; found: 192.13218.

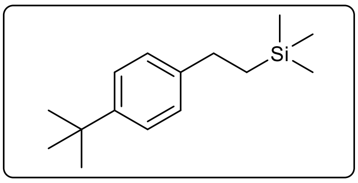

(4-*tert*-Butylphenethyl)trimethylsilane (3aj). ^{1}H NMR (400 MHz, $CDCl_3$) δ 7.30 (d, *J* = 8.2 Hz, 2H), 7.14 (d, *J* = 8.2 Hz, 2H), 2.61-2.57 (m, 2H), 1.31 (s, 9H), 0.88-0.84 (m, 2H), 0.01 (s, 9H). ^{13}C NMR (100 MHz, $CDCl_3$) δ 148.23, 142.25, 127.36, 125.13, 34.31, 31.42, 29.37, 18.56, −1.78. HRMS (EI): calculated for $C_{15}H_{26}Si$ [M+]: 234.17983; found: 234.17988.

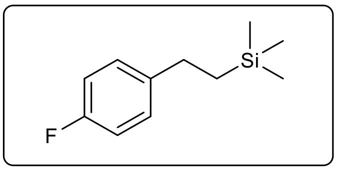

(4-Fluorophenethyl)trimethylsilane (3ak). ^{1}H NMR (400 MHz, $CDCl_3$) δ 7.14 (dd, *J* = 8.2 Hz, 5.6 Hz, 2H), 6.95 (t, *J*= 8.7 Hz, 2H), 2.62-2.58 (m, 2H), 0.87-0.82 (m, 2H), 0.01 (s, 1H). ^{13}C NMR (100 MHz, $CDCl_3$) δ 162.24, 159.83, 140.83, 140.80, 129.06, 128.98, 115.00, 114.79, 29.27, 18.83, −1.76. HRMS (EI): calculated for $C_{11}H_{17}FSi$ [M+]: 196.10781; found: 196.10767.

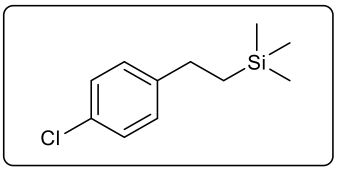

(4-Chlorophenethyl)trimethylsilane (3al). ^{1}H NMR (400 MHz, $CDCl_3$) δ 7.23 (dt, *J* = 8.0 Hz, 2.4 Hz, 2H), 7.12 (d, *J* = 8.5 Hz, 2H), 2.61-2.56 (m, 2H), 0.86-0.81 (m, 2H), 0.01 (s, 9H). ^{13}C NMR (100 MHz, $CDCl_3$) δ 143.72, 131.05, 129.12, 128.29, 29.46, 18.62, −1.77. HRMS (EI): calculated for $C_{11}H_{17}ClSi$ [M+]: 212.07826; found: 212.07851.

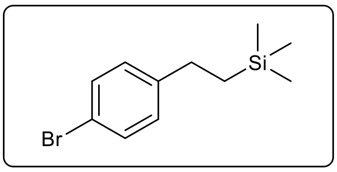

(4-Bromophenethyl)trimethylsilane (3am). ^{1}H NMR (400 MHz, $CDCl_3$) δ 7.38 (d, *J* = 8.3 Hz, 2H), 7.07 (d, *J* = 8.3 Hz, 2H), 2.59-2.55 (m, 2H), 0.85-0.81 (m, 2H), 0.01 (s, 9H). ^{13}C NMR (100 MHz, $CDCl_3$) δ 144.25, 131.25, 129.56, 119.05, 29.532, 18.58, −1.77. HRMS (EI): calculated for $C_{11}H_{17}BrSi$ [M+]: 258.02569; found: 258.02579.

Supplementary References:

1. Wu, J., Yang, X., He, Z., Mao, X., Hatton, T. A., & Jamison, T. F. Continuous Flow Synthesis of Ketones from Carbon Dioxide and Organolithium or Grignard Reagents. *Angew. Chem. Int. Ed*. **53**, 8416-8420 (2014).

2. Vila, C., Giannerini, M., Hornillos, V., Fañanás-Mastral, M. & Feringa, B. L. Palladium-catalysed direct cross-coupling of secondary alkyllithium reagents. *Chem. Sci.* **5**, 1361-1367 (2014).

3. Giannerini, M., Fañanás-Mastral, M. & Feringa, B. L. Direct catalytic cross-coupling of organolithium compounds. *Nat. Chem.* **5**, 667-672 (2013).

4. Kataoka, N., Shelby, Q., Stambuli, J. P. & Hartwig, J. F. Air Stable, Sterically Hindered Ferrocenyl Dialkylphosphines for Palladium-Catalyzed C−C, C−N, and C−O Bond-Forming Cross-Couplings. *J. Org. Chem.* **67**, 5553-5566 (2002).

5. Nakao, Y., Takeda, M., Matsumoto, T. & Hiyama, T. Cross-Coupling Reactions through the Intramolecular Activation of Alkyl(triorgano)silanes. *Angew. Chem. Int. Ed.* **49**, 4447-4450 (2010).

6. Selva, M., Perosa, A., Tundo, P. & Brunelli, D. Selective N,N-Dimethylation of Primary Aromatic Amines with Methyl Alkyl Carbonates in the Presence of Phosphonium Salts. *J. Org. Chem.* **71**, 5770-5773 (2006).

7. Yang, C.-T., Zhang, Z.-Q., Liu, Y.-C. & Liu, L. Copper-Catalyzed Cross-Coupling Reaction of Organoboron Compounds with Primary Alkyl Halides and Pseudohalides. *Angew. Chem. Int. Ed.* **50**, 3904-3907 (2011).

8. Movassaghi, M. & Ahmad, O. K. N-Isopropylidene-N’-2-nitrobenzenesulfonyl Hydrazine, a Reagent for Reduction of Alcohols via the Corresponding Monoalkyl Diazenes. *J. Org. Chem.* **72**, 1838-1841 (2007).

9. Satoh, T., Kondo, A. & Musashi, J. Generation of magnesium carbenoids from 1-chloroalkyl phenyl sulfoxides with a Grignard reagent and applications to alkylation and olefin synthesis. *Tetrahedron* **60**, 5453-5460 (2004).

10. Ackermann, L., Kapdi, A. R. & Schulzke, C. Air-Stable Secondary Phosphine Oxide or Chloride (Pre)Ligands for Cross-Couplings of Unactivated Alkyl Chlorides. *Org. Lett.* **12**, 2298-2301 (2010).
